# Self-assembly of metalla[3]catenanes, Borromean rings and ring-in-ring complexes using a simple π-donor unit

**DOI:** 10.1093/nsr/nwaa164

**Published:** 2020-07-15

**Authors:** Ye Lu, Dong Liu, Yue-Jian Lin, Zhen-Hua Li, Guo-Xin Jin

**Affiliations:** State Key Laboratory of Molecular Engineering of Polymers, Shanghai Key Laboratory of Molecular Catalysis and Innovative Materials, Department of Chemistry, Fudan University, Shanghai 200438, China; State Key Laboratory of Molecular Engineering of Polymers, Shanghai Key Laboratory of Molecular Catalysis and Innovative Materials, Department of Chemistry, Fudan University, Shanghai 200438, China; State Key Laboratory of Molecular Engineering of Polymers, Shanghai Key Laboratory of Molecular Catalysis and Innovative Materials, Department of Chemistry, Fudan University, Shanghai 200438, China; State Key Laboratory of Molecular Engineering of Polymers, Shanghai Key Laboratory of Molecular Catalysis and Innovative Materials, Department of Chemistry, Fudan University, Shanghai 200438, China; State Key Laboratory of Molecular Engineering of Polymers, Shanghai Key Laboratory of Molecular Catalysis and Innovative Materials, Department of Chemistry, Fudan University, Shanghai 200438, China

**Keywords:** coordination-driven self-assembly, supramolecular chemistry, interlocked structure, metalla[3]catenanes, molecular Borromean rings, ring-in-ring complex

## Abstract

Despite extensive research and several stunning breakthroughs in the synthesis of interlocked molecular species, [3]catenanes, Borromean rings and ring-in-ring complexes are exceedingly rare and their targeted synthesis remains a formidable challenge. Herein, a series of Cp^*^Rh-based homogeneous and heterogeneous interlocked structures have been prepared by coordination-driven self-assembly, not only including metalla[2]catenanes and molecular Borromean rings, but also linear metalla[3]catenanes and ring-in-ring complexes. The interlocked structures are all based on bithiophenyl groups. The bithiophenyl groups effectively enhance the strength of the inter-ring interactions and play a crucial role in the formation of these interlocked structures. By taking advantage of the strong interaction between π-donor (**D**) and π-acceptor (**A**) groups, the electron-deficient methylviologen cation was introduced into a cationic metallarectangle based on bithiophenyl groups. Taking inspiration from these results, a cationic metallarectangle based on **A** units was threaded into a metallarectangle based on **D** units, leading to a heterogeneous **D**–**A** ring-in-ring structure.

## INTRODUCTION

**Figure 1.**
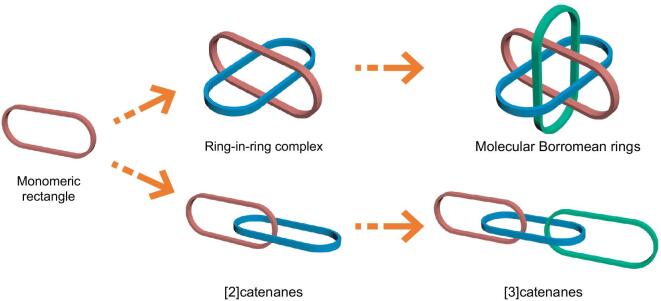
Various interlocked structures: monomeric rectangle; ring-in-ring complex; Borromean rings; [2]catenanes; linear [3]catenanes.

Interlocked molecular species have received considerable attention recently, not only because of their intriguing structures and topological importance [1–5], but also because of their important applications as molecular machines and nanoscale devices [6–10]. In addition to traditional organic macrocycles based on covalent bonds, organometallic macrocycles or rectangles based on coordination self-assembly are promising alternative building blocks for the construction of molecular interlocked structures [11–14]. Benefiting from the reversible coordination bond, some complicated interlocked structures could be realized by high-yield, one-step processes, such as a [2]catenane and Solomon knot [15–20]. Molecular Borromean rings (BRs) are [3]catenane topoisomers in which none of the component rings is linked, but also they cannot be separated without breaking one of the rings (Fig. 1) [21–24]. Linear [3]catenanes form another fascinating interlocked three-ring motif (Fig. 1) [25]. Several effective methods for the construction of organic linear [3]catenanes have been presented [26–29]. However, the feasible strategies for the synthesis of organometallic linear metalla[3]catenanes based on coordination-driven self-assembly are still very rare [30]. We reasoned that strong stacking interactions may be crucial for the formation of a self-assembled interlocked trimer structure. Due to the large electron cloud of the sulfur atom, S-containing heterocyclic compounds usually present stronger stacking interactions than polycyclic aromatic compounds under similar conditions [31]. In order to enhance the stacking interactions, we turned to bithiophenyl groups as building blocks to replace the widely used phenylene or polycyclic aromatic groups. In the meantime, bithiophenyl groups have received considerable attention as functional building blocks in the construction of supramolecular architectures [32–34]. We hoped to obtain more intricate interlocked trimer structures by enhance stacking interactions, such as BRs and linear metalla[3]catenanes (Fig. 1).

Beyond linear [3]catenanes, ring-in-ring complexes are also very rare structural motifs, which can be considered as substructures of BRs and key intermediates for the preparation of BRs (Fig. 1) [35–37]. Inspired by the extremely challenging goal of developing a targeted synthesis of ring-in-ring complexes, we considered whether a more direct and simple method might be possible by using bithiophenyl groups. Electrostatic interactions between electron-rich (π-donor, **D**) and electron-deficient (π-acceptor, **A**) aromatic groups are important driving forces in host–guest chemistry [38,39]. Metallarectangles or cages based on coordination self-assembly commonly bear several positive charges [40,41]. Due to Coulombic repulsion, this type of metallarectangle or cage is more suitable for combination with electroneutral or electron-rich guests than with electron-poor cations, and overcoming the Coulombic repulsion between a cationic guest and a cationic host is still a challenge [42–44]. Bithiophenyl groups are strong **D** units, thus their introduction into metallarectangles could lead to strong interactions between **D** units and **A** units, which is a promising strategy to overcome the Coulombic repulsion and potentially allow the introduction of a positively-charged cation inside a positively-charged cationic metallarectangle. Following this logic, if an electron-deficient cation could be introduced into a cationic metallarectangle by taking advantage of strong **D**–**A** interactions, it could also be possible to thread a cationic metallarectangle based on **A** units inside a metallarectangle based on **D** units, to obtain a heterogeneous **D**–**A** ring-in-ring complex.

Herein, we report a series of Cp^*^Rh-based (Cp^*^=pentamethylcyclopentadienyl) homogeneous metalla[2]catenanes, as well as linear metalla[3]catenanes and BRs structures through the use of building blocks based on bithiophenyl groups—a simple π-donor unit. Bithiophenyl groups play a crucial role in the formation of the homogeneous interlocked structures, namely enhancing the strength of the inter-ring interactions. By taking advantage of strong electrostatic interactions between **D** and **A** units, we further used the electron-deficient methylviologen cation as a guest molecule to realize reversible conversion between a [2]catenane and a monomeric rectangle (MR). Furthermore, a cationic metallarectangle based on **A** units was threaded inside a metallarectangle based on bithiophenyl groups, leading to a heterogeneous ring-in-ring complex. This method for forming a ring-in-ring complex was extended by the use of a metallarectangle based on a pyrenyl group.

## RESULTS AND DISCUSSION

**Scheme 1.**
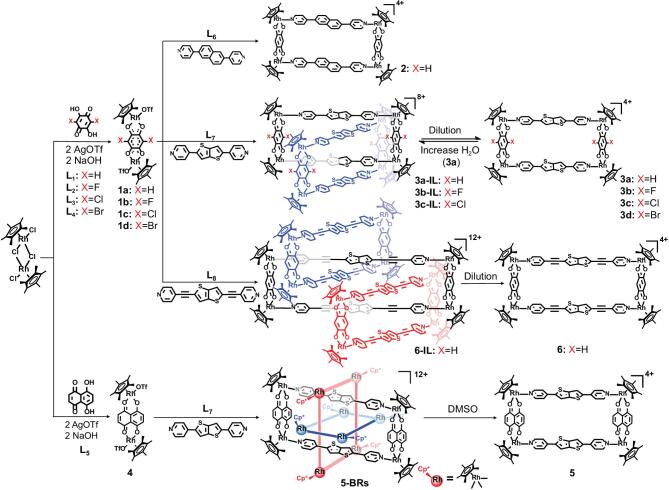
Synthesis and conversion of monomeric rectangle and homogeneous metalla[2]catenanes, metalla[3]catenanes and BRs. Two rings of BRs are simplified as two rectangles (blue and red) for clarity.

In the field of self-assembled interlocked structures, π–π stacking is one of the most common driving forces. The optimal distance for this interaction is ∼3.5 Å, hence we chose the precursor **1a** (based on 2,5-dihydroxy-1,4-benzoquinoneas (**L_1_**)) as the binuclear precursor in this work as its metal–metal distance is ∼7.9 Å, which is close to twice the conventional distance of π–π stacking (7 Å) [45]. Stirring of a 1 : 1 mixture (1.0 mM) of precursor **1a** and a pyridyl ligand based on the naphthyl group 2,6-di(pyridin-4-yl)naphthalene (**L_6_**) in CD_3_OD for 12 h at room temperature resulted in a clear brown solution. The nuclear magnetic resonance (NMR) spectra clearly indicated the quantitative self-assembly of molecular rectangle **2** (Scheme 1 and Supplementary Figs 1–4). Single-crystal X-ray crystallographic analysis further established the structure of **2** to be a discrete MR structure (Supplementary Fig. 51).

Then, we attempted to use bithiophenyl groups to augment the π–π stacking interactions. Thereby, 2,5-di(pyridin-4-yl)thieno[3,2-b]thiophene (**L_7_**) was used in place of **L_6_** in the metallarectangle construction. The two linear pyridyl ligands **L_6_** and **L_7_** differ only in their aromatic group, i.e. naphthyl and bithienyl, respectively. We first attempted to use the linear pyridyl ligand **L_7_** (0.2 mM) and precursor **1a** (0.2 mM) to build the metallarectangles under similar reaction conditions to those used above. The ^1^H NMR spectrum of the solution clearly indicated the quantitative self-assembly of molecular rectangle **3a** and displays the the typical signals of a MR structure (Supplementary Figs 5–7), similar to before. As the reaction concentration increased to 0.5 mM, new signals were observed in the ^1^H NMR spectrum (Supplementary Fig. 8), along with peaks from **3a**, indicating the formation of a new compound (**3a-IL**). By increasing the concentration of the reactant (from 0.5 to 5.0 mM), the proportion of new compound **3a-IL** in the methanol solution also increased gradually (Supplementary Fig. 8). In 5.0 mM methanol solution, the proportion of **3a-IL** was >50% (Supplementary Fig. 9). The proportion of **3a-IL** could be increased further to ∼65% by the addition of D_2_O to the CD_3_OD solution (Supplementary Fig. 10). That means the hydrophobic interaction also plays an important role in the formation of [2]catenanes. Addition of *d_6_*-DMSO converted **3a-IL** to its corresponding MR **3a** (Supplementary Fig. 11). DOSY and ^1^H-^1^H COSY NMR spectra further confirmed these findings (Supplementary Figs 12–14).

**Figure 2.**
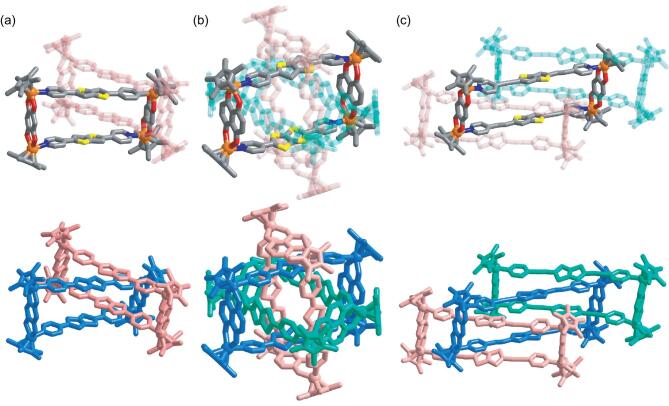
Molecular structures of one component ring of cationic **3a-IL** (a), **5-BRs** (b), **6-IL** (c) (up) and their respective full structures colored according to unit (down). Hydrogen atoms, anions, solvent molecules and disorder are omitted for clarity. N, blue; O, red; S, yellow; C, gray; Rh, orange.

Brown block crystals of **3a-IL** were isolated in 92.3% yield upon recrystallization by vapor diffusion. Single-crystal X-ray crystallographic analysis established the structure of **3a-IL** to be a discrete interlocked [2]catenane structure (Fig. 2a). The angle between the two metallarectangles is 50.3°. The four planes of the bithienyl groups are nearly parallel to each other and the distance of each plane is ∼3.5 Å, which is in accordance with the conventional distance of π–π interactions. These results demonstrate that a bithiophenyl group effectively enhances the π–π stacking and plays a crucial role in forming the metalla[2]catenanes. Density Functional Theory (DFT) binding energy calculations were performed to further prove this (Supplementary Table 1 and Supplementary Fig. 55).

The transformation between the metalla[2]catenane and the MR also could be observed via absorption spectra, as the strong π–π interaction leads to effective charge transformation and red shifts of absorption bands. As shown in Supplementary Fig. 58, the absorption of **3a** (0.01 mM, methanol solution) is mainly in the ultraviolet- and blue-light regions (<500 nm). Upon formation of the interlocked structure (**3a-IL**), the absorption in the visible-light region was significantly enhanced (500–700 nm).

Inspired by these results, we attempt to use the bithiophenyl group to construct more interesting interlocked structures. **L_7_** was chosen as the pyridyl ligand to construct BRs because of its narrow profile and electron-rich central unit. The N···N distance of **L_6_** is ∼12.4 Å, so, in order to meet our empirical rule for constructing BRs based on π–π stacking, we need to employ a longer binuclear clip than precursor **1a** [45]. Thus, complex **4** based on naphthazarine **L_5_** was selected as the binuclear precursor in view of its suitable length (8.3 Å). As expected, **5-BRs** was obtained by a high-yield (89.2%) reaction of precursor **4** with **L_7_** in the methanol solution. A single-crystal X-ray crystallographic analysis established **5-BRs** to have a discrete BRs structure (Fig. 2b), which is further confirmed by ESI-MS (Supplementary Figs 46 and 47). The distances between an S atom from **L_6_** and the plane of naphthazarine are ∼3.5 Å, which is in accordance with the conventional distance of π–π interactions. Presumably due to the suitable aspect ratio of the rectangles, **5-BRs** display high stability in methanol solution (Supplementary Figs 15–19). There is no equilibrium between BRs and corresponding MRs in methanol solutions of **5-BRs**. Subsequently, upon increasing the ratio of *d_6_*-DMSO to CD_3_OD, a clear signal attributable to the corresponding MR, **5**, was observed (Supplementary Figs 20 and 21), which was further confirmed by ^13^C, ^1^H-^1^H COSY and DOSY NMR spectroscopy (Supplementary Figs 22–24).

BRs and linear [3]catenane structures both comprise three rings, but their mode of interlocking is different. The use of metallarectangles with smaller aspect ratios is beneficial to the formation of BRs, thus we attempted to deliberately increase the aspect ratio to avoid the formation of BRs. Hereby, 2,5-bis(pyridin-4-ylethynyl)thieno[3,2-b]thiophene (**L_8_**) was employed as an analog of **L_7_** lengthened by two alkynyl groups (Scheme 1). The two linear pyridyl ligands **L_7_** and **L_8_** differ only in their length; the N···N distances of **L_7_** and **L_8_** are ∼12.4 and 18.2 Å, respectively. Thereby, stirring of a 1 : 1 mixture (1.0 mM) of precursor **1a** and **L_8_** in CD_3_OD for 12 h at room temperature resulted in a clear brown solution. NMR spectra clearly indicated the quantitative self-assembly of molecular rectangle **5** (Supplementary Figs 25–27). When the reaction concentration was increased to 3.0 mM, new peaks were observed in the ^1^H NMR spectrum, along with peaks from **5**, indicating the formation of a new compound: **6-IL** (Supplementary Figs 28 and 29). By increasing the concentration of the reactants (from 1.0 to 5.0 mM), the proportion of new compound **6-IL** in methanol solution also increased gradually and DOSY NMR spectroscopy further confirmed the presence of two complexes in the solution (Supplementary Fig. 30).

Gratifyingly, brown block crystals of **6a-IL** were isolated in 90.7% yield upon recrystallization by vapor diffusion. The single-crystal X-ray crystallographic analysis established that **6-IL** possessed an interlocked trimer structure, but did not consist of BRs. In **6-IL**, three rings are interlocked in a linear [3]catenane structure (Fig. 2c), which is further confirmed by ESI-MS (Supplementary Fig. 48). In this structure, the long pyridyl ligand **L_8_** allows two bithienyl groups from two metallarectangles to thread inside a further metallarectangle. Four bithienyl groups from the two side metallarectangles stack with the two alkynyl groups of the middle metallarectangle, respectively. The distances from the carbon atoms of alkynyl groups to the plane formed by the bithienyl unit are ∼3.5 Å, which is in accordance with the conventional distance of π–π interactions.

**Figure 3.**
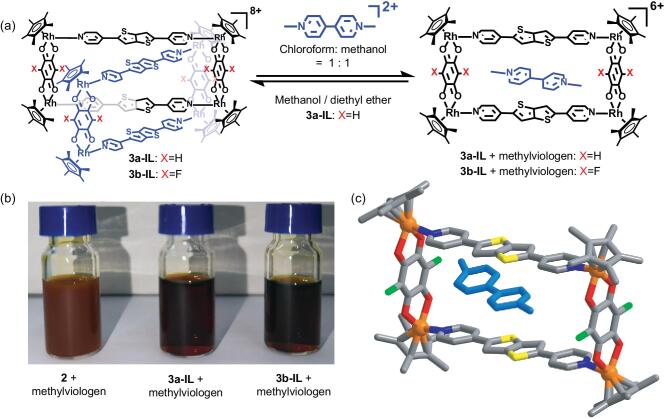
(a) Reversible conversion of metalla[2]catenanes and corresponding MR by using methylviologen as guest; (b) pictures of **2**, **3a-IL** and **3b-IL** added by methylviologen ditriflate respectively in mixed (3.0 mM) CHCl_3_/CH_3_OH solution (1 : 1 v/v); only the solution of MR **2** based on the naphthyl group is cloudy; (c) single-crystal X-ray structures of cationic **3b** with encapsulated methylviologen. N, blue; O, red; C, black; Rh, orange; F, green, S, yellow; hydrogen atoms and counter anions are omitted.

Encouraged by these results, we sought to further explore the application of the bithiophenyl group. Metallarectangles based on Cp^*^Rh/Ir corners commonly have four positive charges, so this kind of metallarectangle is more suitable for electroneutral and negatively-charged guests than for positively-charged guests, likely due to Coulombic repulsion [40]. Some cations feature strongly electron-accepting π-systems, such as paraquat or methylviologen (*N*, *N*^′^-dimethyl-4,4^′^-bipyridinium), the chloride salt of which is known as a highly toxic pesticide [46]. As the bithiophenyl group is a strong **D** unit, it is possible to apply the strong interaction between **D** units and **A** units to overcome the Coulombic repulsion. Along these lines, we added methylviologen ditriflate (1 equiv.) to a mixture of **3a-IL** (1 equiv. each) in a mixed (3.0 mM) CHCl_3_/CH_3_OH solution (1 : 1 v/v) (Fig. 3a). After roughly 2 h, we were delighted to observe that the methylviologen had dissolved, resulting in a clear red solution (Fig. 3b). Methylviologen is well known for its very poor solubility in most organic solvents. The observed dissolution of methylviologen in CHCl_3_ and CH_3_OH suggested that the methylviologen dication may have been encapsulated by the metallarectangle, greatly improving its solubility. For proving this, the same experiment was performed on MR **2** based on the naphthyl group, resulting in a cloudy solution (Fig. 3b). That means the bithiophenyl group played a very important role in improving the interaction between cationic metallarectangles and the methylviologen cation and enhancing the solubility of the methylviologen in organic solvent. NMR spectroscopy confirmed the encapsulation of methylviologen by **3a**; furthermore, the methylviologen induced the transformation of [2]catenane **3a-IL** to the corresponding MR **3a** (Supplementary Figs 31 and 32). The absorption band of **3a** was red shifted after encapsulating the methylviologen dication (Supplementary Fig. 59). This kind of red shift could be attributed to the charge transfer between metallarectangle **3a** and the methylviologen guest. Unfortunately, all attempts to obtain single crystals of **3a**-encapsulated methylviologen by diffusion of diethyl ether into the mixed CHCl_3_/CH_3_OH solution were unsuccessful, as, upon crystallization, the **3a**-encapsulated methylviologen reverted back to **3a-IL**, as indicated by analysis of the unit cell parameters of the crystals (Fig. 3a). This may be due to the interaction between two metallarectangles being stronger than the interaction between the metallarectangles and the methylviologen dication in the solid state, so that the methylviologen is squeezed out of the metallarectangle upon crystallization.

For finding out more information and the X-ray structure of the cationic metallarectangle-encapsulated methylviologen cation, we need to find out a method to weaken the interaction between two metallarectangles but keep the strong π-donor unit intact. Analysis of the structure of **3a-IL** showed that the hydrogen atom on the short-arm linker is very close to a hydrogen atom on a pyridyl ligand of the other metallarectangle; the distance between the two hydrogen atoms is ∼2.4 Å (Supplementary Fig. 52). We reasoned that replacement of one of the two hydrogen atoms by a larger atom, e.g. a halide atom, may weaken the interaction between the two metallarectangles. Thus, **L_1_** was replaced by the fluoranilic acid (**L_2_**), chloranilic acid (**L_3_**) and bromoanilic acid (**L_4_**) under identical reaction conditions (Scheme 1). As a result, we found that the introduction of halides on the short-arm linker successfully reduced the proportion of metalla[2]catenanes in the methanol solution under the same concentration and weakened the inter-ring interaction (for details, see Supplementary Data). Based on these results, we attempted the same experiment using **3b-IL** (based on fluoranilic acid) under similar conditions (Supplementary Figs 36–38). As expected, single-crystal X-ray crystallographic analysis unequivocally demonstrated that the methylviologen dication was present inside the metallarectangle **3b** as a guest molecule (Fig. 3c).

**Figure 4.**
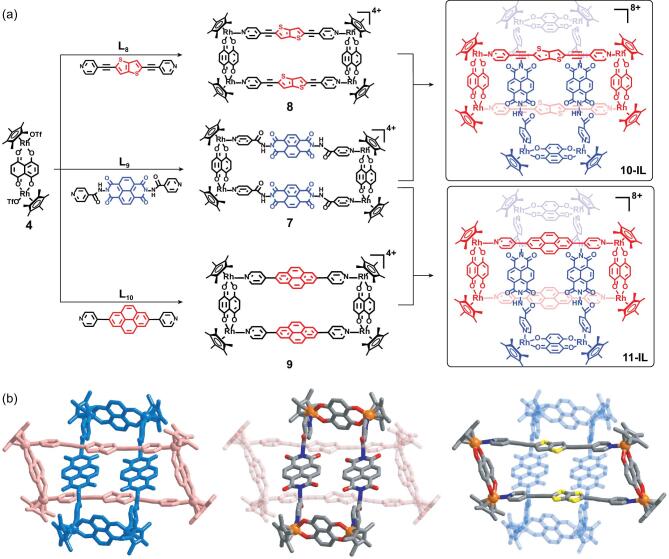
(a) Synthesis heterogeneous ring-in-ring complex; (b) single-crystal X-ray structures of cationic **10-IL**. N, blue; O, red; S, yellow; C, gray; Rh, orange; hydrogen atoms and counter anions are omitted.

Given the successful encapsulation of a cationic guest inside a cationic metallarectangle by taking advantage of strong interactions between **D** and **A** units, we next sought to thread another cationic metallarectangle based on **A** units into a metallarectangle based on **D** units using similar interactions. Hence, we prepared a pyridyl ligand with an electron-deficient naphthalenediimide (NDI) unit, **L_9_** (*N*,*N*^′^-(1,3,6,8-tetraoxo-1,3,6,8-tetrahydrobenzo [*lmn*][3,8] phenanthroline-2,7-diyl)diisonicotinamide), which led to construction of **A** metallarectangle **7** by combination with precursor **4** (Fig. 4a and Supplementary Figs 39 and 40). In order to construct a MR capable of accommodating two NDI units in its central cavity, the longer pyridyl ligand based on the bithienyl group (**L_8_**) was selected to react with precursor **4**, leading to the π-donor metallarectangle **8** (Fig. 4a and Supplementary Figs 41 and 42). The reason for using precursor **4** is to avoid the formation of [3]catenane. Stirring of a 1 : 1 mixture (5.0 mM) of bithienyl metallarectangle **8** and NDI metallarectangle **7** in CD_3_OD and *d_6_*-DMSO (CD_3_OD : *d_6_*-DMSO = 5 : 1) resulted in a clear green solution. The NMR spectrum of the solution clearly indicated the formation of new compound **10-IL** (Supplementary Fig. 43), which was further confirmed by DOSY NMR (Supplementary Fig. 44). According to the relative intensity of the Cp^*^ signal, the proportion of **10-IL** was ∼70% in a 5.0 mM methanol solution. ESI-MS further indicated that the new compound **10-IL** is a dimer formed by one **D** metallarectangle **8** and one **A** metallarectangle **7** (Supplementary Fig. 49).

Gratifyingly, we were able to grow single crystals of **10-IL**. Single-crystal X-ray crystallographic analysis confirmed **10-IL** to possess a heterogeneous **D–A** ring-in-ring structure in which the **A** metallarectangle **7** was threaded inside the **D** metallarectangle **8** (Fig. 4b). Two NDI units were accommodated inside metallarectangle **7**, further (covalently) linked to each other by two units of **4**, leading to the ring-in-ring structures. The planes of the bithiophenyl groups are nearly parallel to those of the NDI units and the distances between the planes are ∼3.4 Å, which is in accordance with the conventional distance of π–π interactions. There is no direct evidence indicating the formation of a heterogeneous [2]catenane during the reaction. This may be due to the fact that the ring-in-ring complex provides optimal space occupancy in this case. DFT-binding-energy calculations were performed to further confirm this (Supplementary Table 1 and Supplementary Figs 56 and 57). The similar ring-in-ring complex (**11-IL**) also could be realized by using pyridyl ligand (**L_10_**) as the **D** unit (Fig. 4a), which is confirmed by ESI-MS and single-crystal X-ray crystallographic analysis (Supplementary Figs 45, 50 and 54).

## CONCLUSION

In this work, the bithienyl group, a simple π-donor unit, is used as a building block to construct a series of homogeneous metalla[2]catenanes, linear metalla[3]catenanes and BRs. Bithienyl groups play a crucial role in the formation of these homogeneous interlocked structures, by effectively enhancing the strength of the inter-ring interactions. Due to strong donor–acceptor interaction, the electron-deficient methylviologen dication could be used as a guest molecule, overcoming the Columbic repulsion, and effected reversible conversion between metalla[2]catenanes and MR structures. Extending this principle into two dimensions, heterogeneous ring-in-ring structures were obtained by incorporation of a cationic metallarectangle based on NDI units inside a metallarectangle based on bithienyl or pyrenyl groups. We hope that our findings will help to further the field of advanced self-assembly.

## METHODS

### Crystallographic details

Crystallographic data for complex **2** was collected at 173 K using a CCD-Bruker APEX DUO system (Mo*_K__α_*, λ = 0.71073 Å). Those of **3a-IL**, **3d**, **5-BRs**, **6-IL**, **3b** with encapsulated methylviologen, **10-IL** and **11-IL** were collected at 173 or 203 K using a CCD-Bruker SMART APEX system (Ga*_K__α_*, λ = 1.34138 Å). The single-crystal X-ray diffraction data of **2**, **3a-IL**, **3d**, **5-BRs**, **6-IL**, **3b** with encapsulated methylviologen, **10-IL** and **11-IL** have been deposited in the Cambridge Crystallographic Data Centre under accession number CCDC: 1 882 402 (**2**), 1 882 426 (**3a-IL**), 1 882 425 (**5-BRs**), 1 882 431 (**6-IL**), 1 882 433 (**3d**), 1 882 432 (**3b** with encapsulated methylviologen), 1 882 436 (**10-IL**) and 1 882 435 (**11-IL**).

## Supplementary Material

nwaa164_Supplementary_files
