# Supplementary material for: Self-assembly of metalla[3]catenanes, Borromean rings and ring-in-ring complexes using a simple π-donor unit
Source: Natl Sci Rev. 2020 Jul 15;7(10):1548–56. doi: 10.1093/nsr/nwaa164 (PMC8290965; doi:10.1093/nsr/nwaa164)
Supplement: nwaa164_Supplementary_files [file nwaa164_supplementary_files.docx]

**Self-assembly of Metalla[3]catenanes, Borromean rings and Ring-in-ring Complex using a simple π-donor unit**

Ye Lu, Dong Liu, Yue-Jian Lin, Zhen-Hua Li and Guo-Xin Jin*

State Key Laboratory of Molecular Engineering of Polymers, Shanghai Key Laboratory of Molecular Catalysis and Innovative Materials, Department of Chemistry, Fudan University

2005 Songhu road, Shanghai, 200438 (P. R. China)

E-mail: [gxjin@fudan.edu.cn](mailto:gxjin@fudan.edu.cn)

**Contents**

[Experimental Procedures 3](#_Toc38616795)

[Results and Discussion 7](#_Toc38616796)

[NMR Spectrum 8](#_Toc38616797)

[ESI-MS 31](#_Toc38616798)

[Single-crystal X-ray structures 33](#_Toc38616799)

[DFT Calculation 35](#_Toc38616800)

[UV-VIS absorption spectra 37](#_Toc38616801)

[X-ray crystal data 38](#_Toc38616802)

[Reference 46](#_Toc38616803)

Experimental Procedures

**General Procedures.** All reagents and solvents were purchased from commercial sources and used as supplied without additional purification unless otherwise mentioned. The starting materials [Cp*RhCl_2_]_2_ and 2,6-di(pyridin-4-yl)naphthalene (**L_6_**) were prepared by literature method [S1]. NMR spectra (^1^H, ^13^C, ^1^H-^1^H COSY and DOSY) were recorded on Bruker AVANCE I 400 or Bruker AVANCE III 400 spectrometers. Chemical shifts (δ) are expressed in ppm downfield from tetramethylsilane using the residual protonated solvent as an internal standard. Complex multiplets are noted as “m” and broad resonances as “br”. Elemental analyses were performed on an Elementar Vario EL III analyzer. IR spectra of the solid samples (KBr tablets) in the range 400-4000 cm^-1^ were recorded on a Nicolet AVATAR-360IR spectrometer. Mass spectra were obtained with Micro TOF II mass spectrometer.

**Preparation of 2,5-di(pyridin-4-yl)thieno[3,2-b]thiophene (L_7_).** A flask was charged with 1.48 g (5.2 mmol) of 2,5-dibromothieno[3,2-b]thiophene, 2.5 g (18 mmol) of pyridine-4-boronic acid, 300 mg (0.33 mmol) of Pd_2_(DBA)_3_, and 225 mg (0.8 mmol) of P(Cy)_3_ and purged with N_2_. The mixture was suspended in 40 mL of deoxygenated 1,4-dioxane. A solution of 4 g (19 mmol) of K_3_PO_4_ in 20 mL of degassed water was added by syringe through a septum. The reaction was heated at reflux under N_2_ overnight. Upon being cooled to room temperature, the mixture was poured into a separatory funnel and the lower aqueous phase removed and discarded. The dioxane layer was collected and filtered and the dioxane removed under reduced pressure. The residue was dissolved in chloroform and washed twice with 5 g of Na_2_CO_3_ in 25 mL of water. The chloroform solution was dried with anhydrous magnesium sulfate and the solvent removed under reduced pressure. The solid was recrystallized by ethyl acetate. 2,5-di(pyridin-4-yl)thieno[3,2-b]thiophene (**L_7_**) was obtained as a brown powder in a 50.8 % yield (0.77 g). ^1^H NMR (400 MHz, CDCl_3_, ppm): δ = 8.66 (d, *J* =6 Hz, 4H, Py-H), 7.73 (s, 2H, bithiophenyl-H), 7.53 (d, *J* = 6 Hz, 2H, Py-H); Anal. calcd for C_16_H_10_N_2_: C 65.28, H 3.42, N 9.52, found: C 65.25, H 3.46, N 9.48.

**Preparation of 2,5-bis(pyridin-4-ylethynyl)thieno[3,2-b]thiophene (L_8_).** A flask was charged with 232 mg (0.78 mmol) of 2,5-dibromothieno[3,2-b]thiophene, 160 mg (1.56 mmol) of 4-ethynylpyridine, 30 mg (0.16 mmol) of CuI, 56 mg (0.08 mmol) of bis(triphenylphosphine)palladium(II) chloride and purged with N_2_. The mixture was suspended in 30 mL of deoxygenated DMF and triethylamine. The ratio of DMF and triethylamine is about 2:1. The reaction was heated at reflux under N_2_ three days. Upon being cooled to room temperature, all of the solvent removed under reduced pressure. The residue was dissolved in chloroform and filtered and the filtrate washed twice with 5 g of Na_2_CO_3_ in 50 mL of water. The chloroform solution was dried with anhydrous magnesium sulfate and the solvent removed under reduced pressure. The raw product was further purified by flash column chromatography, eluant is chloroform. 2,5-bis(pyridin-4-ylethynyl)thieno[3,2-b]thiophene (**L_8_**) was obtained as a yellow powder in a 32.5 % yield (87 mg). ^1^H NMR (400 MHz, CDCl_3_, ppm): δ = 8.64 (d, *J* =4.8 Hz, 4H, Py-H), 7.47 (s, 2H, bithiophenyl-H), 7.39 (d, *J* =4.8 Hz, 4H, Py-H); Anal. calcd for C_20_H_10_N_2_S_2_: C 70.15, H 2.94, N 8.18, found: C 70.19, H 2.99, N 8.11.

**Preparation of** ***N*,*N*'-(1,3,6,8-tetraoxo-1,3,6,8-tetrahydrobenzo[*lmn*][3,8]phenanthroline-2,7-diyl)diisonicotinamide (L_9_).** A flask was charged with 1,4,5,8-naphthalenetetracarboxylic dianhydride (2.68 g, 10 mmol) and isoniazid (2.74 g, 20 mmol). The mixture was suspended in 40 mL of dry DMF and refluxed overnight. Upon being cooled to room temperature, the solid was collected by filtration and washed by methanol. The solid was recrystallized by DMF. The product was obtained as pink powder in a 40.3 % yield (2.04 g). ^1^H NMR (400 MHz, *d_6_*-DMSO, ppm): δ = 11.94 (s, 2H, NH-H), 8.87 (d, *J* = 4.8 Hz 4H, Py-H), 8.84 (s, 4H, naphthyl-H), 7.93 (d, *J* = 4.8 Hz 4H, Py-H); Anal. calcd for C_26_H_14_N_6_O_6_: C 61.66.15, H 2.79, N 16.59, found: C 61.59, H 2.71, N 16.50.

**Preparation of 2,7-di(pyridin-4-yl)pyrene (L_10_).** A flask was charged with 1.87 g (5.2 mmol) of 2,7-dibromopyrene, 2.5 g (18 mmol) of pyridine-4-boronic acid, 300 mg (0.33 mmol) of Pd_2_(DBA)_3_, and 225 mg (0.8 mmol) of P(Cy)_3_ and purged with N_2_. The mixture was suspended in 40 mL of deoxygenated 1,4-dioxane. A solution of 4 g (19 mmol) of K_3_PO_4_ in 20 mL of degassed water was added by syringe through a septum. The reaction was heated at reflux under N_2_ overnight. Upon being cooled to room temperature, the mixture was poured into a separatory funnel and the lower aqueous phase removed and discarded. The dioxane layer was collected and filtered and the dioxane removed under reduced pressure. The residue was dissolved in chloroform and washed twice with 5 g of Na_2_CO_3_ in 25 mL of water. The chloroform solution was dried with anhydrous magnesium sulfate and the solvent removed under reduced pressure. The solid was recrystallized by ethyl acetate. 2,7-di(pyridin-4-yl)pyrene (**L_9_**) was obtained as a brown powder in a 54.1 % yield (1.01 g). ^1^H NMR (400 MHz, CDCl, ppm): δ = 8.82 (d, *J* =6.4 Hz, 4H, Py-H), 8.48 (s, 4H, pyrene-H), 8.22 (s, 4H, pyrene-H), 7.84 (d, *J* = 6.4 Hz, 4H, Py-H); Anal. calcd for C_26_H_16_N_2_: C 87.62, H 4.52, N 7.86, found: C 87.61, H 4.50, N 7.88.

**Preparation of 2.** A CH_3_OH solution of [Cp*RhCl_2_]_2_ (124 mg, 0.2 mmol) was added to a solution of 2,5-dihydroxycyclohexa-2,5-diene-1,4-dione (**L_1_**) (28 mg, 0.2 mmol) and NaOH (16 mg, 0.4 mmol) in CH_3_OH (40 mL), and the suspension was stirred at room temperature for 6 h. AgOTf (102.8 mg, 0.4 mmol) was added to the mixture and stirred for 3 h, followed by filtration to remove insoluble compounds (AgCl and NaCl). 2,6-di(pyridin-4-yl)naphthalene (**L_6_**) (56.4 mg, 0.2 mmol) was then added to the filtrate. After the solution was stirred at room temperature for 12 h, the reaction mixture was concentrated to a volume of 3 mL under reduced pressure, filtered through Celite and recrystallized by slow diffusion of diethyl ether into the filtrate (including several drops of DMSO). A brown crystalline solid was obtained in 90.4% yield (216.0 mg); ^1^H NMR (400 MHz, CD_3_OD, ppm): δ = 8.38 (d, *J* = 6.4 Hz, 8H, Py-H), 8.25 (s, 4H, naphthaline-H), 7.97 (d, *J* = 8.8 Hz, 4H, naphthaline-H), 7.95 (d, *J* = 6.4 Hz, 8H, Py-H), 7.81 (d, *J* = 8.8 Hz, 4H, naphthaline-H), 5.72 (s, 4H, benzoquinone-H), 1.71 (s, 60H, Cp*); Anal. calcd for C_96_H_92_F_12_N_4_O_20_Rh_4_S_4_: C 48.25, H 3.88, N 2.34, found: C 48.31, H 3.80, N 2.23; IR (KBr disk, cm^−1^) *v* = 1607, 1529, 1424, 1375, 1257, 1224, 1159, 1031, 820, 639, 573, 517, 497.

**Preparation of 3a-IL.** The synthesis of **3a-IL** was carried out similarly to that of **2** with the use of 2,5-di(pyridin-4-yl)thieno[3,2-b]thiophene **(L_7_)** (58.8 mg, 0.2 mmol) instead of 2,6-di(pyridin-4-yl)naphthalene (**L_6_**). **3a-IL** was obtained as a brown solid in a 92.3% yield (246.1 mg); ^1^H NMR (400 MHz, CD_3_OD, ppm): δ = 8.27 (m, 8H, Py-H), 8.18 (d, *J* = 5.2 Hz, 8H, Py-H), 7.59 (s, 4H, bithiophenyl-H), 7.53 (d, *J* = 5.2 Hz, 8H, Py-H), 7.33 (d, *J* = 5.2 Hz, 8H, Py-H), 7.08 (br, 4H, bithiophenyl-H), 5.89 (s, 8H, benzoquinone-H), 1.80 (s, 60H, Cp*), 1.68 (s, 60H, Cp*); Anal. calcd for C_176_H_168_F_24_N_8_O_40_Rh_8_S_16_: C 43.79, H 3.51, N 2.32, found: C 43.70, H 3.57, N 2.41; IR (KBr disk, cm^−1^) *v* = 1604, 1528, 1419, 1376, 1258, 1224, 1164, 1031, 820, 639, 574, 517, 497.

**Preparation of 5-BRs.** The synthesis of **5-BRs** was carried out similarly to that of **3a-IL** with the use of naphthazarine **L_5_** (38.0 mg, 0.2 mmol) instead of 2,5-dihydroxycyclohexa-2,5-diene-1,4-dione (**L_1_**). **5-BRs** was obtained as a green solid in a 89.2% yield (224.1 mg); ^1^H NMR (400 MHz, CD_3_OD, ppm): δ = 9.10 (d, *J* = 6.0 Hz, 24H, Py-H), δ = 7.94 (d, *J* = 6.0 Hz, 24H, Py-H), 6.57 (s, 24H, naphthazarine-H), 5.68 (s, 12H, bithiophene -H), 1.67 (s, 180H, Cp*); Anal. calcd for C_288_H_264_F_36_N_12_O_60_Rh_12_S_24_: C 45.87, H 3.53, N 2.23, found: C 45.82, H 3.59, N 2.33; ESI-MS *m*/*z*: [**5-BRs**-3OTf]^3+^ Calcd. 2364.3615, found 2364.3524; [**5-BRs**-4OTf]^4+^ Calcd. 1736.0331, found 1736.0817;IR (KBr disk, cm^−1^) *v* = 1601, 1532, 1414, 1270, 1223, 1157, 1065, 1030, 962, 638, 574, 517, 443.

**Preparation of 6-IL.** The synthesis of **6-IL** was carried out similarly to that of **3a-IL** with the use of 2,5-bis(pyridin-4-ylethynyl)thieno[3,2-b]thiophene **(L_8_)** (68.4 mg, 0.2 mmol) instead of 2,5-di(pyridin-4-yl)thieno[3,2-b]thiophene **(L_7_)**. **6-IL** was obtained as a brown solid in a 90.7% yield (227.5 mg); ^1^H NMR (400 MHz, CD_3_OD, ppm): δ = 8.52 (m), 8.18 (m), 7.98 (br), 7.28 (br), 6.85 (br), 6.52 (br), 6.06 (s), 5.83 (s), 5.80 (br), 1.82 (br), 1.72 (s), 1.65 (s); Anal. calcd for C_288_H_252_F_36_N_12_O_60_Rh_12_S_24_: C 45.94, H 3.37, N 2.23, found: C 45.99, H 3.32, N 2.15; ESI-MS *m*/*z*: [**6-IL**-4OTf]^4+^ Calcd. 1733.0096, found 1733.1495; IR (KBr disk, cm^−1^) *v* = 1601, 1525, 1432, 1374, 1251, 1234, 1169, 1011, 822, 658, 548, 517, 434.

**Preparation of 3b.** The synthesis of **3b** was carried out similarly to that of **3a-IL** with the use of fluoranilic acid **(L_2_)** (35.2 mg, 0.2 mmol) instead of 2, 5-dihydroxycyclohexa-2,5-diene-1,4-dione (**L_1_**). **3b** was obtained as a brown solid in a 91.3% yield (226.9 mg); ^1^H NMR (400 MHz, CD_3_OD, ppm): δ = 8.30 (d, *J* = 6.4 Hz, 8H, Py-H), 8.04 (s, 4H, bithiophene-H), δ = 7.78 (d, *J* = 6.4 Hz, 8H, Py-H), 1.72 (s, 60H, Cp*); Anal. calcd for C_88_H_80_F_16_N_4_O_20_Rh_4_S_8_: C 42.52, H 3.24, N 2.25, found: C 42.48, H 3.29, N 2.29; IR (KBr disk, cm^−1^) *v* = 1604, 1512, 1421, 1360, 1259, 1222, 1161, 1031, 824, 574, 517, 499.

**Preparation of 3c.** The synthesis of **3c** was carried out similarly to that of **3a-IL** with the use of chloranilic acid **(L_3_)** (41.6 mg, 0.2 mmol) instead of 2,5-dihydroxycyclohexa-2,5-diene-1,4-dione (**L_1_**). **3c** was obtained as a brown solid in a 89.3% yield (227.7 mg); ^1^H NMR (400 MHz, CD_3_OD, ppm): δ = 8.30 (d, *J* = 6.4 Hz, 8H, Py-H), 8.05 (s, 4H, bithiophene-H), δ = 7.77 (d, *J* = 6.4 Hz, 8H, Py-H), 1.72 (s, 60H, Cp*); Anal. calcd for C_88_H_80_Cl_4_F_12_N_4_O_20_Rh_4_S_8_: C 41.43, H 3.16, N 2.20, found: C 41.35, H 3.23, N 2.28; IR (KBr disk, cm^−1^) *v* = 1604, 1514, 1421, 1371, 1259, 1222, 1160, 1031, 840, 638, 5474, 517.

**Preparation of 3d.** The synthesis of **3d** was carried out similarly to that of **3a-IL** with the use of bromianilic acid **(L_4_)** (59.4 mg, 0.2 mmol) instead of 2,5-dihydroxycyclohexa-2,5-diene-1,4-dione (**L_1_**). **3d** was obtained as a brown solid in a 90.8% yield (247.6 mg); ^1^H NMR (400 MHz, CD_3_OD, ppm): δ = 8.32 (d, *J* = 6.4 Hz, 8H, Py-H), 8.09 (s, 4H, bithiophene-H), δ = 7.81 (d, *J* = 6.4 Hz, 8H, Py-H), 1.73 (s, 60H, Cp*); Anal. calcd for C_88_H_80_Br_4_F_12_N_4_O_20_Rh_4_S_8_: C 38.73, H 2.95, N 2.05, found: C 38.67, H 2.99, N 2.12; IR (KBr disk, cm^−1^) *v* = 1604, 1537, 1434, 1359, 1258, 1222, 1168, 1031, 825, 640, 573, 518.

**Preparation of 7.** A CH_3_OH solution of [Cp*RhCl_2_]_2_ (124 mg, 0.2 mmol) was added to a solution of naphthazarine (38.0 mg, 0.2 mmol) and NaOH (16 mg, 0.4 mmol) in CH_3_OH (40 mL), and the suspension was stirred at room temperature for 6 h. AgOTf (102.8 mg, 0.4 mmol) was added to the mixture and stirred for 3 h, followed by filtration to remove insoluble compounds (AgCl and NaCl). *N*,*N*'-(1,3,6,8-tetraoxo-1,3,6,8-tetrahydrobenzo[*lmn*][3,8]phenanthroline-2,7-diyl)diisonicotinamide **(L_9_)** (101.2 mg, 0.2 mmol) was then added to the filtrate. After the solution was stirred at room temperature for 12 h, the reaction mixture was concentrated to a volume of 3 mL under reduced pressure, filtered through Celite and recrystallized by slow diffusion of diethyl ether into the filtrate (including several drops of DMSO). A brown crystalline solid was obtained in a 88.2% yield (259.1 mg); ^1^H NMR (400 MHz, CD_3_OD and *d_6_*-DMSO, ppm): δ = 8.82 (d, *J* = 4.8 Hz, 8H, Py-H), δ = 8.80 (s, 8H, naphthaline-H), δ = 8.15 (d, *J* = 4.8 Hz, 8H, Py-H), 7.34 (s, 8H, naphthazarine-H), 1.71 (s, 60H, Cp*); Anal. calcd for C_116_H_96_F_12_N_12_O_32_Rh_4_S_4_: C 47.42, H 3.29, N 5.72, found: C 47.35, H 3.23, N 5.79; *v* = 1697, 1660, 1582, 1488, 1446, 1415, 1352, 1253, 1160, 1031, 826, 759, 639, 574, 517.

**Preparation of 8.** The synthesis of **8** was carried out similarly to that of **7** with the use of 2,5-bis(pyridin-4-ylethynyl)thieno[3,2-b]thiophene **(L_8_)** (68.4 mg, 0.2 mmol) instead of *N*,*N*'-(1,3,6,8-tetraoxo-1,3,6,8-tetrahydrobenzo[*lmn*][3,8]phenanthroline-2,7-diyl)diisonicotinamide **(L_9_)**. **8** was obtained as a green solid in a 89.7% yield (234.1 mg); ^1^H NMR (400 MHz, CD_3_OD, ppm): δ = 8.44 (d, *J* = 5.6 Hz, 8H, Py-H), δ = 7.59 (s, 4H, bithiophene-H), δ = 7.54 (d, *J* = 5.6 Hz, 8H, Py-H), 7.24 (s, 8H, naphthazarine-H), 1.64 (s, 60H, Cp*); Anal. calcd for C_104_H_88_F_12_N_4_O_20_Rh_4_S_8_: C 47.86, H 3.40, N 2.15, found: C 47.80, H 3.47, N 2.10; *v* = 1601, 1552, 1411, 1394, 1247, 1222, 1152, 1031, 826, 618, 528, 517, 494.

**Preparation of 9.** The synthesis of **9** was carried out similarly to that of **8** with the use of 2,7-di(pyridin-4-yl)pyrene **(L_10_)** (71.2 mg, 0.2 mmol) instead of 2,5-bis(pyridin-4-ylethynyl)thieno[3,2-b]thiophene **(L_8_)**. **9** was obtained as a green solid in a 89.9% yield (237.1 mg); ^1^H NMR (400 MHz, *d_6_*-DMSO, ppm): δ = 8.58 (s, 8H, pyrenyl-H), δ = 8.53 (d, *J* = 6 Hz, 8H, Py-H), δ = 8.13 (s, 8H, pyrenyl-H), δ = 8.10 (d, *J* = 6 Hz, 8H, Py-H), 7.30 (s, 8H, naphthazarine-H), 1.59 (s, 60H, Cp*); Anal. calcd for C_116_H_100_F_12_N_4_O_20_Rh_4_S_4_: C 52.82, H 3.82, N 2.12, found: C 52.91, H 3.88 N 2.19; *v* = 1610, 1534, 1416, 1384, 1269, 1224, 1159, 1031, 842, 638, 548, 517.

**Preparation of 10-IL.** Stirring of a 1:1 mixture of bithienyl metallarectangle **8** (260.9 mg, 0.1 mmol) and NDI metallarectangle **7** (293.8 mg, 0.1 mmol) in CH_3_OH (including several drops of DMSO) for 12 h at room temperature resulted in a clear green solution. The reaction mixture was concentrated to a volume of 3 mL under reduced pressure, filtered through Celite and recrystallized by slow diffusion of diethyl ether into the filtrate. A brown crystalline solid was obtained in 91.2% yield (505.9 mg) Anal. calcd for C_220_H_184_F_24_N_16_O_52_Rh_8_S_12_: C 47.63, H 3.34, N 4.04, found: C 47.71, H 3.25 N 4.09; ESI-MS *m*/*z*: [**10-IL**-3OTf]^3+^ Calcd. 1700.0813, found 1700.0836; *v* = 1606, 1533, 1441, 1374, 1255, 1225, 1161, 1031, 854, 759, 639, 547, 517.

**Preparation of 11-IL.** Stirring of a 1:1 mixture of metallarectangle **9** (263.7 mg, 0.1 mmol) and NDI metallarectangle **7** (293.8 mg, 0.1 mmol) in CH_3_OH (including several drops of DMSO) for 12 h at room temperature resulted in a clear green solution. The reaction mixture was concentrated to a volume of 3 mL under reduced pressure, filtered through Celite and recrystallized by slow diffusion of diethyl ether into the filtrate. A brown crystalline solid was obtained in 91.5% yield (510.1mg) Anal. calcd for C_232_H_196_F_24_N_16_O_52_Rh_8_S_8_: C 49.98, H 3.54, N 4.02, found: C 49.90, H 3.62 N 4.10; ESI-MS *m*/*z*: [**11-IL**-3OTf]^3+^ Calcd. 1709.4836, found 1708.4873; *v* = 1610, 1534, 1450, 1256, 1159, 1031,963, 853, 759, 639, 575, 517.

**Crystallographic Details.** Crystallographic data for complexes **2** was collected at 173 K using a CCD-Bruker APEX DUO system (Mo_Kα_, λ = 0.71073 Å). Those of **3a-IL**, **3d**, **5-BRs**, **6-IL**, **3b** with encapsulated methylviologen, **10-IL** and **11-IL** were collected at 173 K or 203 K using a CCD-Bruker SMART APEX system (Ga_Kα_, λ = 1.34138 Å). Indexing was performed using APEX 2 (difference vectors method). Data integration and reduction were performed using SaintPlus 6.01. Absorption correction was performed by the multiscan method implemented in SADABS. The structures were solved and refined using SHELXTL-97. The single-crystal X-ray diffraction data of **2**, **3a-IL**, **3d**, **5-BRs**, **6-IL**, **3b** with encapsulated methylviologen, **10-IL** and **11-IL** have been deposited in the Cambridge Crystallographic Data Centre under accession number CCDC: 1882402 (**2**), 1882426 (**3a-IL**), 1882425 (**5-BRs**), 1882431 (**6-IL**), 1882433 (**3d**), 1882432 (**3b** with encapsulated methylviologen), 1882436 (**10-IL**) and 1882435 (**10-IL**). The CIF files of **2**, **3a-IL**, **3d**, **5-BRs**, **6-IL**, **3b** with encapsulated methylviologen, **10-IL** and **11-IL** are available at supplementary data.

Results and Discussion

**Introduction of halides on the short-arm linker**

**L_1_** was replaced with the fluoranilic acid (**L_2_**), chloranilic acid (**L_3_**), and bromoanilic acid (**L_4_**) under identical reaction conditions, resulting in the formation of new dirhodium precursors **1b** (based on fluoranilic acid), **1c** (based on chloranilic acid) and **1d** (based on bromanilic acid) (Scheme 1). The four proligands differ only in their aromatic substituents, i.e., H, F, Cl and Br, respectively. The precursors **1b**, **1c** and **1d** reacted, in turn, with **L_7_**, providing MRs **3b** and **3c**, respectively, at low concentrations (Scheme 1 and supplementary Figs S33 and S34). At high concentrations, interlocked chains **3b-IL** and **3c-IL** were formed, but with a key difference: the proportion of interlocked structures in the solution is gradually decreased with increasing atomic radius of the aromatic substituent. According to integration of the ^1^H NMR spectra, the proportions of **3a-IL** (based on L_1_), **3b-IL** (based on fluoranilic acid) and **3c-IL** (based on chloranilic acid) were ca. 36%, 23% and 11% in the 3.0 mM methanol solutions, respectively (Supplementary Figs S8, S33 and S34). Furthermore, for bromanilic acid, no interlocked complex was observed even in the 3.0 mM methanol solution (Supplementary Fig. S35), allowing us to successfully grow single crystals of MR **3d**. A single-crystal X-ray crystallographic analysis unequivocally confirmed the MR structure of **3d** (Supplementary Fig. S53). Thus, the introduction of halides on the short-arm linker successfully weakened the inter-ring interaction and kept the strong π-donor unit intact.

**Reversible conversion between 3a-IL and 3a with methylviologen cation**

Methylviologen ditriflate (1 equiv.) was added into a mixture of **3a-IL** (1 equiv. each) in a mixed (3.0 mM) CHCl_3_ / CH_3_OH solution (1:1 v/v). After roughly 2 h, the methylviologen had dissolved and resulting in a clear red solution. NMR spectroscopy confirmed the encapsulation of methylviologen by **3a**, furthermore the methylviologen induced the transformation of [2]catenanes **3a-IL** to the corresponding MR **3a** (Supplementary Figs S31 and S32). However, the reaction mixture was concentrated to a volume of 1 mL under reduced pressure, filtered through Celite and recrystallized by slow diffusion of diethyl ether into the filtrate. The **3a**-encapsulated methylviologen reverted back to **3a-IL** during recrystallization, as indicated by analysis of the unit cell parameters of the crystals (Fig. 3a).

NMR Spectrum


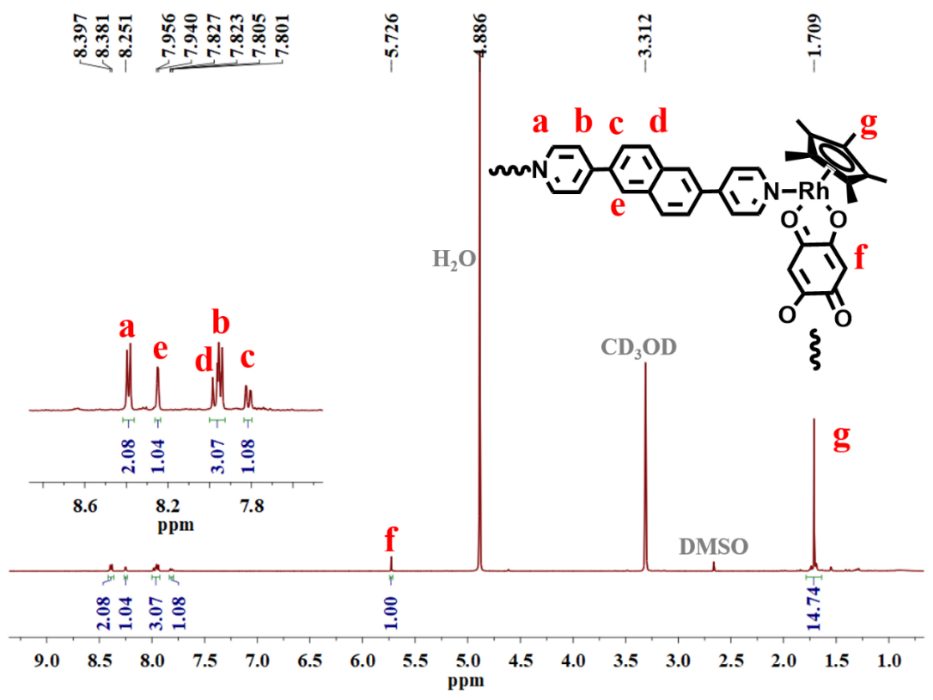


**Figure S1**. ^1^H NMR spectrum of **2** (CD_3_OD, [1.0mM], 298K, 400 MHz)


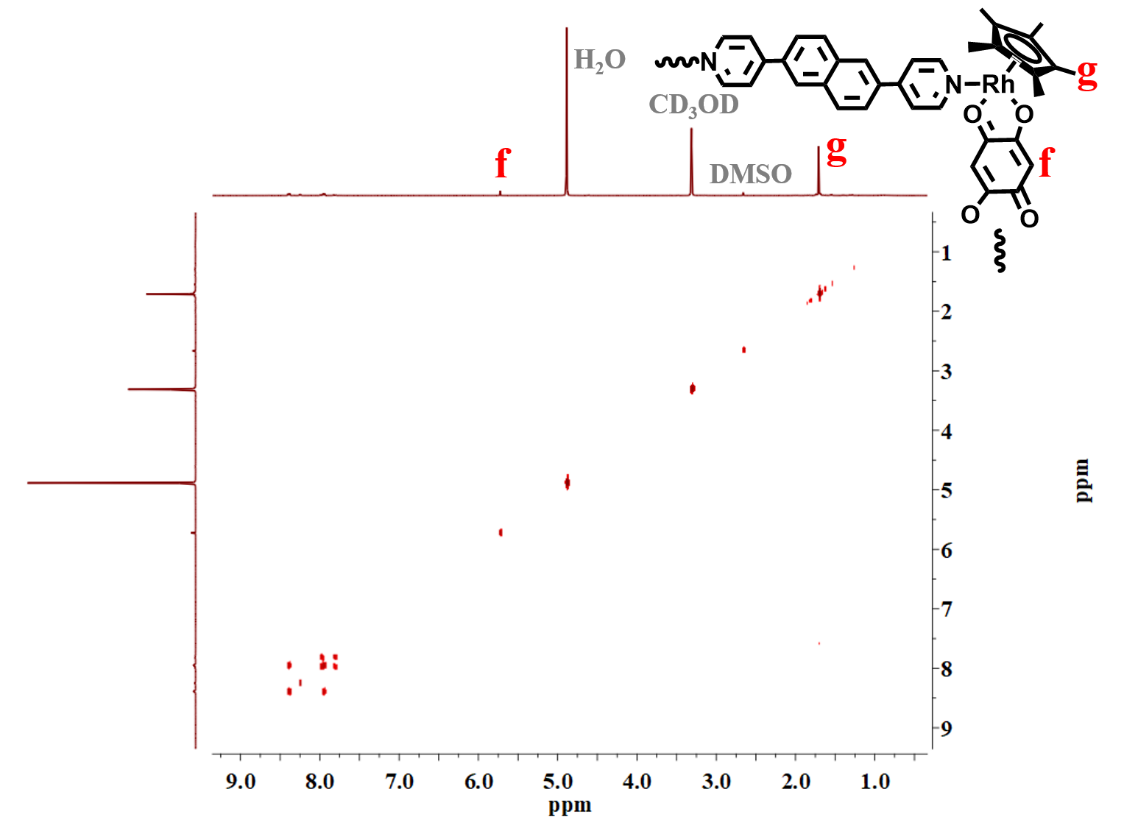


**Figure S2**. ^1^H-^1^H COSY NMR spectrum of **2** (CD_3_OD, [1.0mM], 298K, 400 MHz)


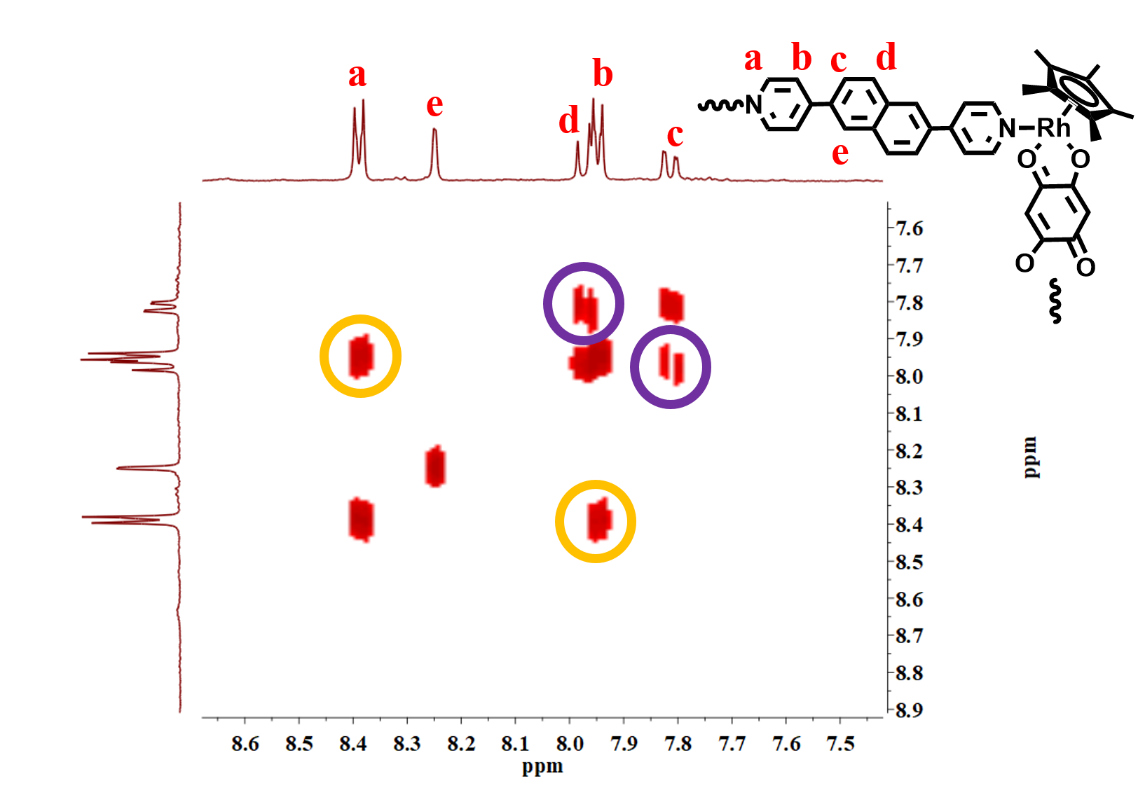


**Figure S3**. Partial ^1^H-^1^H COSY NMR spectrum of **2** (CD_3_OD, [1.0mM], 298K, 400 MHz)


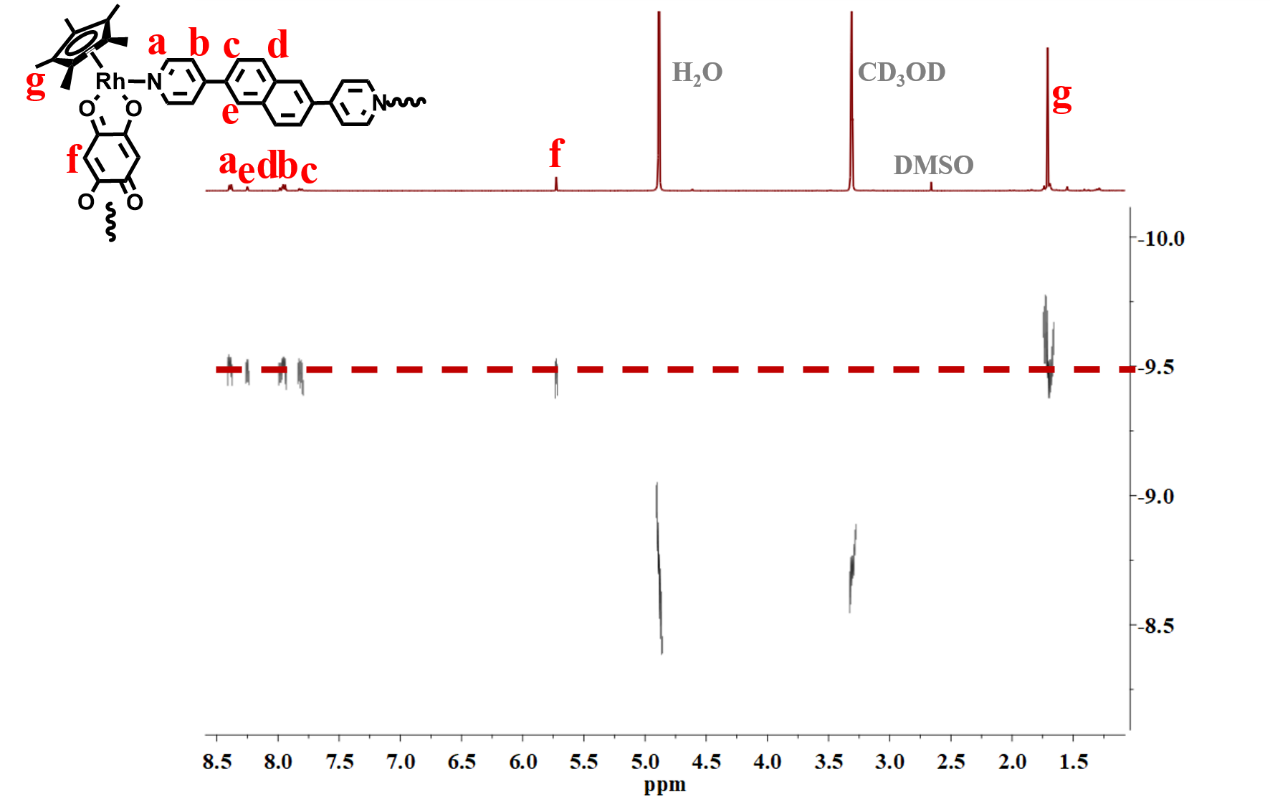


**Figure S4**. ^1^H DOSY NMR spectrum of **2** (CD_3_OD, [1.0 mM], 298 K, 400 MHz)


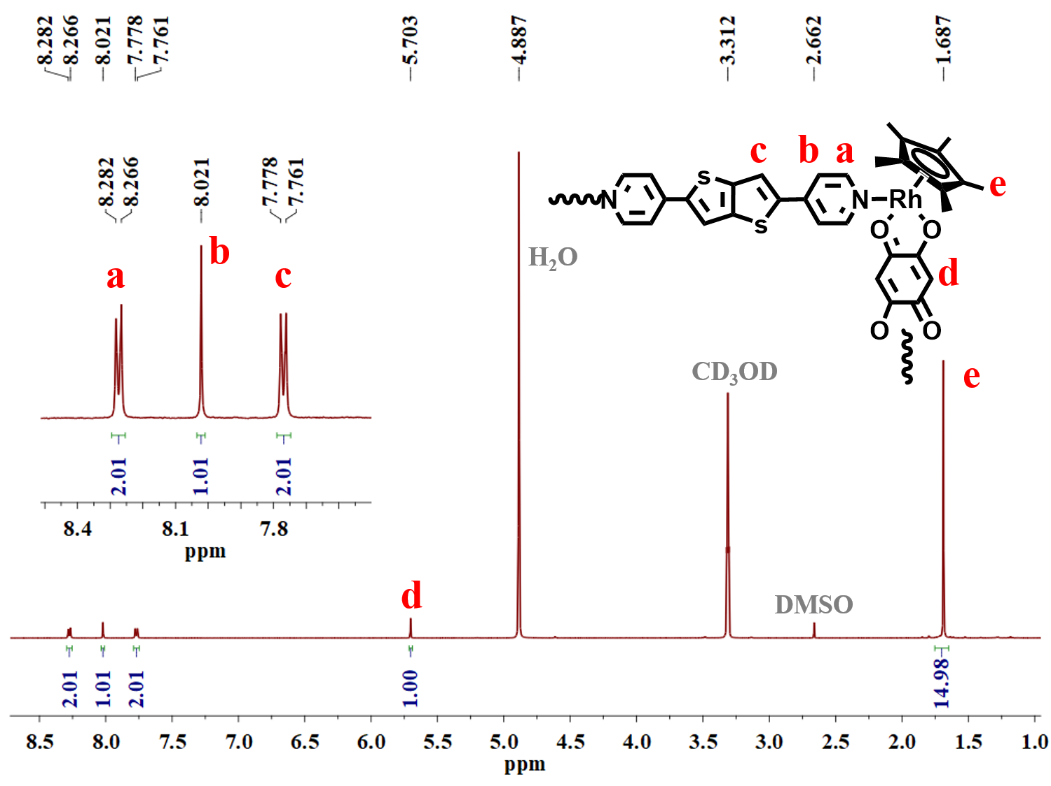


**Figure S5**. ^1^H NMR spectrum of **3a** (CD_3_OD, [0.2 mM], 298K, 400 MHz)


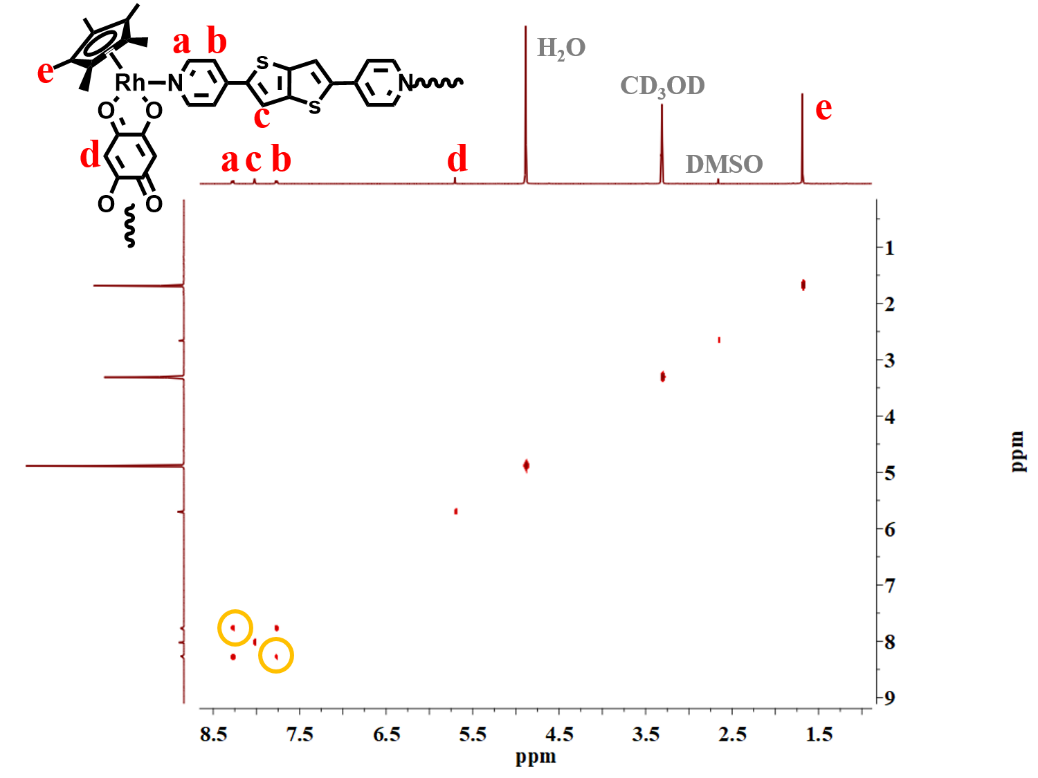


**Figure S6**. ^1^H-^1^H COSY NMR spectrum of **3a** (CD_3_OD [0.2mM], 298K, 400 MHz)


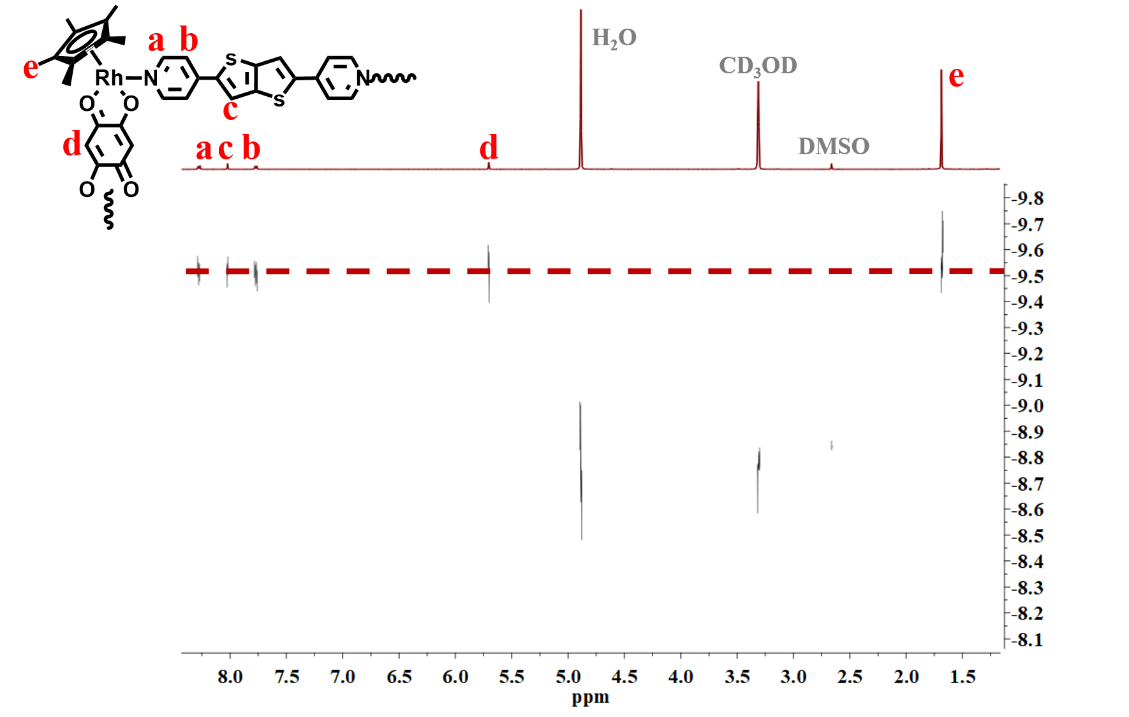


**Figure S7**. ^1^H DOSY NMR spectrum of **3a** (CD_3_OD, [0.2 mM], 298K, 400 MHz).


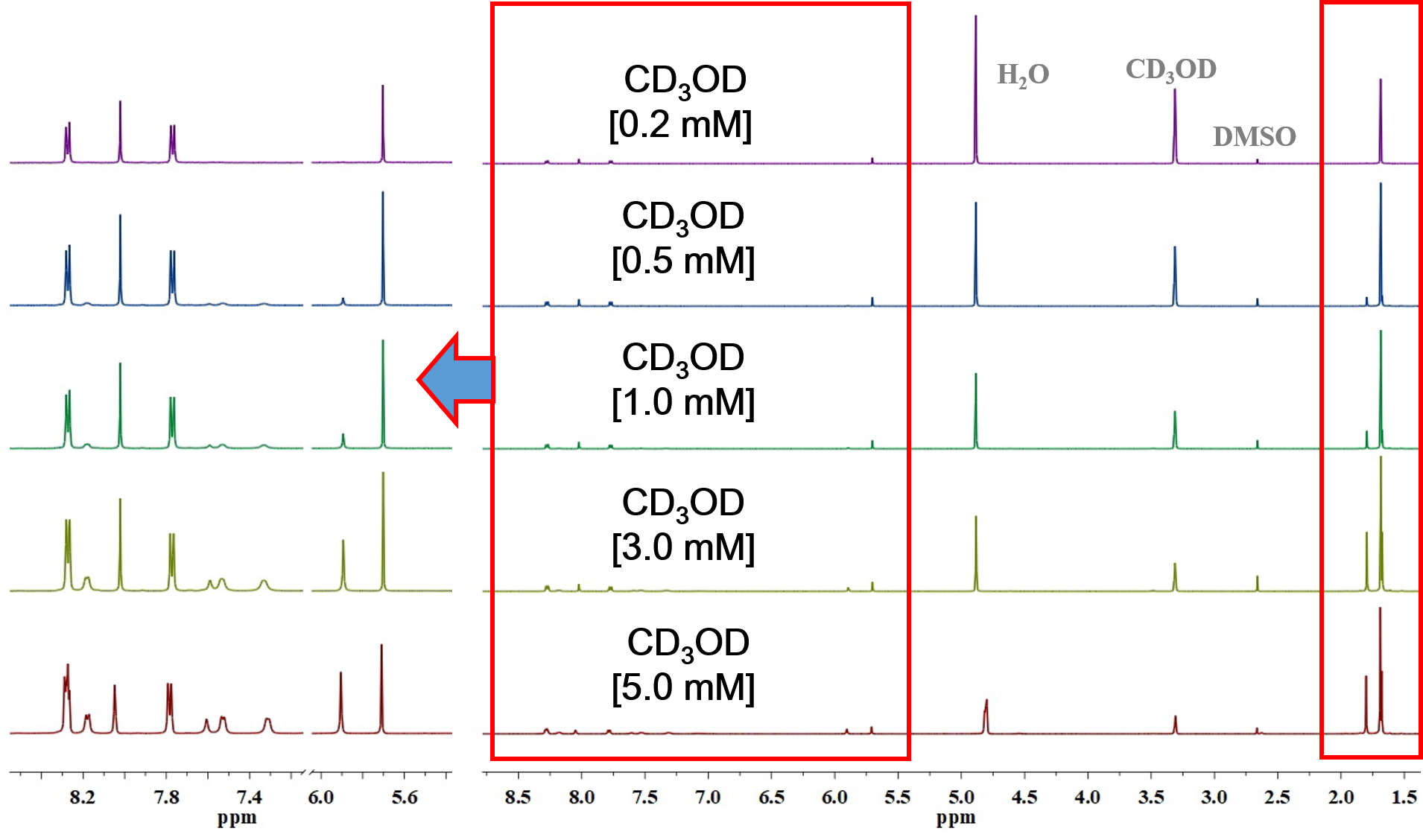


**Figure S8**. ^1^H NMR spectrum of showing transformation from **3a** to [**3a** + **3a-IL**] up increasing the concentration from 0.2 mM to 5.0 mM ([0.2 mM], [0.5 mM], [1.0 mM], [3.0 mM], [5.0 mM], 298K, 400 MHz).


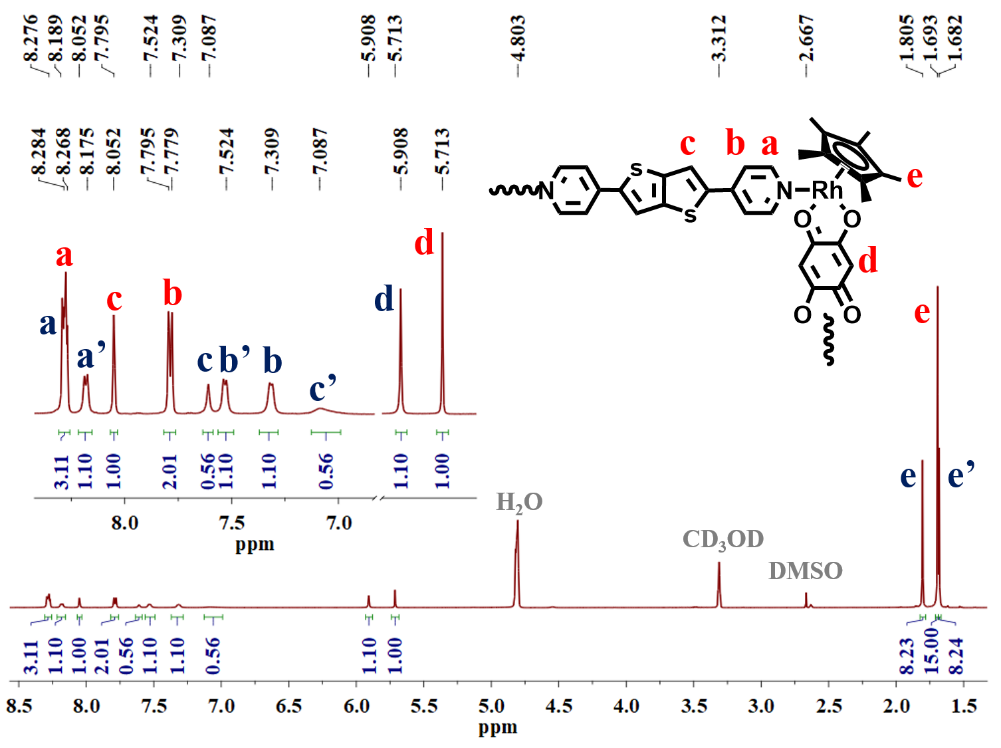


**Figure S9**. ^1^H NMR spectrum of **3a** + **3a-IL** (CD_3_OD, [5.0mM], 298K, 400 MHz).


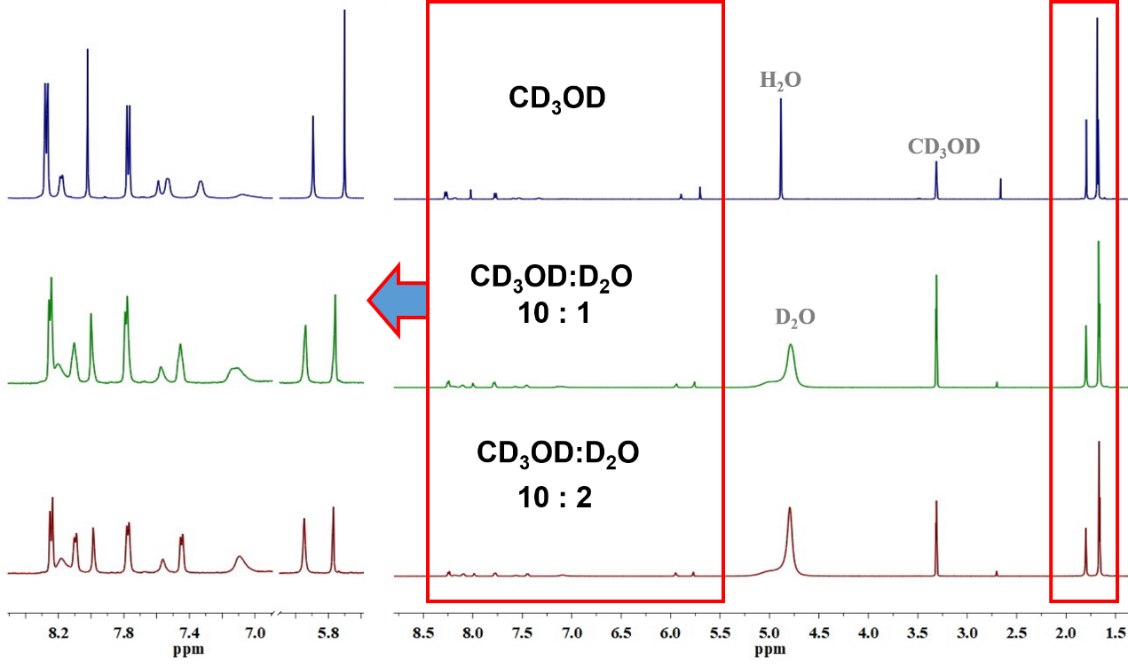


**Figure S10**. ^1^H NMR spectra of mixture of **3a** + **3a-IL** in CD_3_OD [5.0 mM], **3a** +**3a-IL** in CD_3_OD and D_2_O [5.0 mM] (CD_3_OD:D_2_O = 10:1), **3a** + **3a-IL** in CD_3_OD and D_2_O [5.0 mM] (CD_3_OD:D_2_O = 10:2). Limited by the solubility, the proportion of D_2_O can’t be further increased, D_2_O has caused extensive precipitation in the mixture solution of CD_3_OD and D_2_O (CD_3_OD:D_2_O = 10:2).


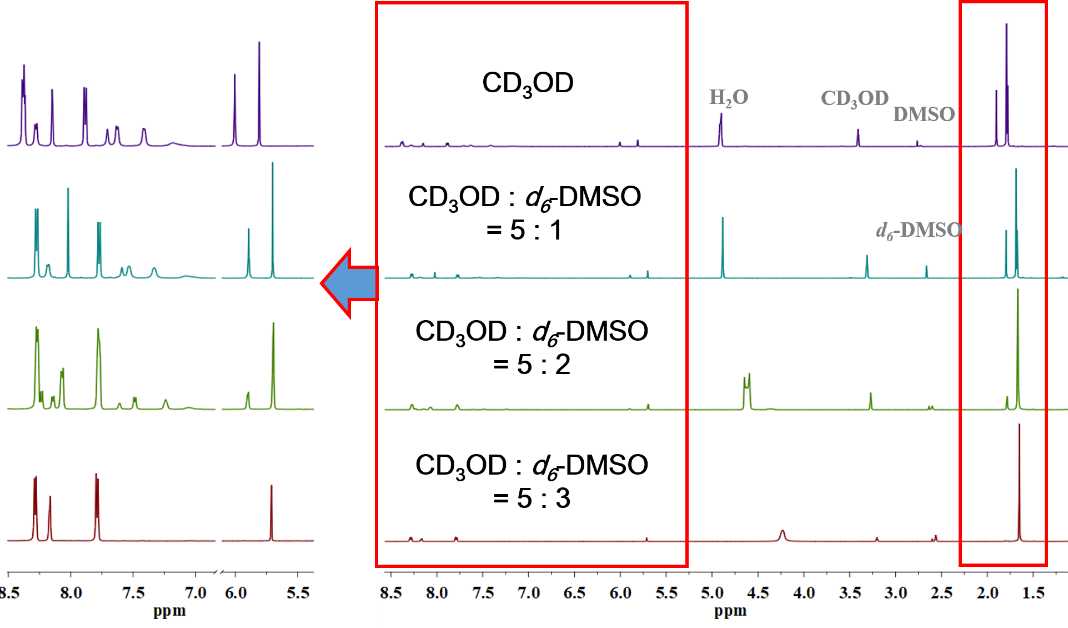


**Figure S11**. ^1^H NMR spectrum of **3a** + **3a-IL** in CD_3_OD ([5.0 mM]), **3a** + **3a-IL** in CD_3_OD and *d*_6_-DMSO (CD_3_OD: *d*_6_-DMSO = 5:1 [5.0mM]), **3a** + **3a-IL** in CD_3_OD and *d*_6_-DMSO (CD_3_OD: *d*_6_-DMSO = 5:2 [5.0mM]), **3a** in CD_3_OD and *d*_6_-DMSO (CD_3_OD: *d*_6_-DMSO = 5:3 [5.0mM]).


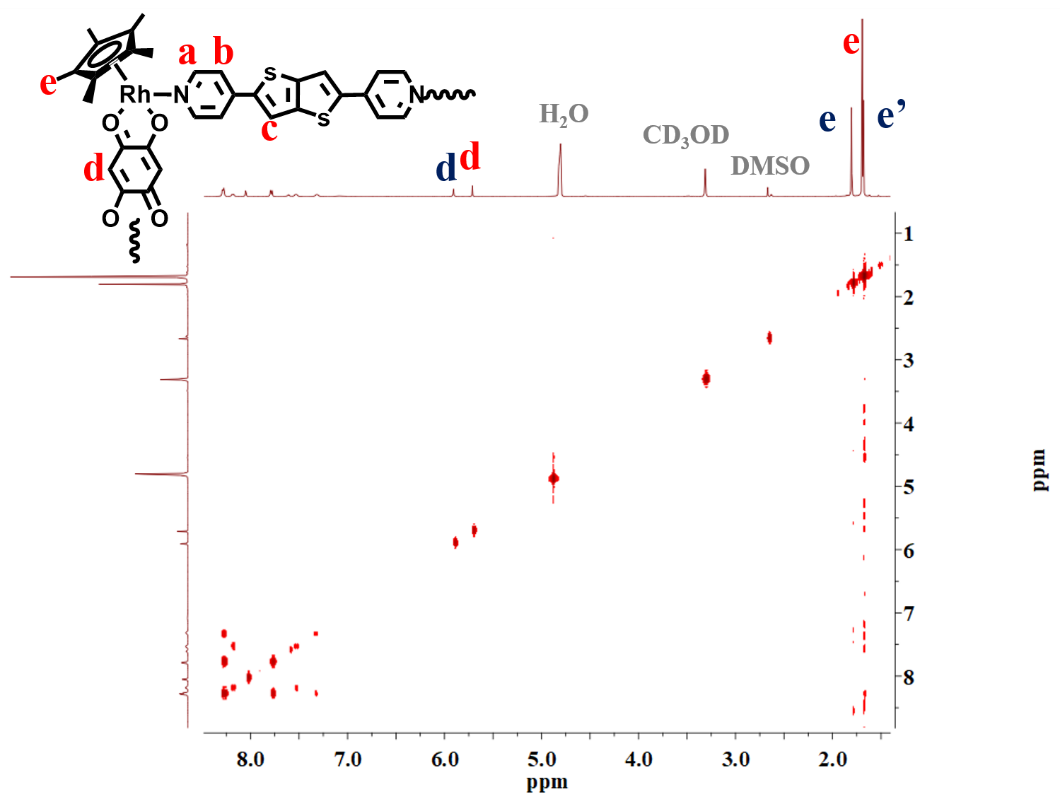


**Figure S12**. ^1^H-^1^H COSY NMR spectrum of **3a** + **3a-IL** (CD_3_OD [5.0mM], 298K, 400 MHz).


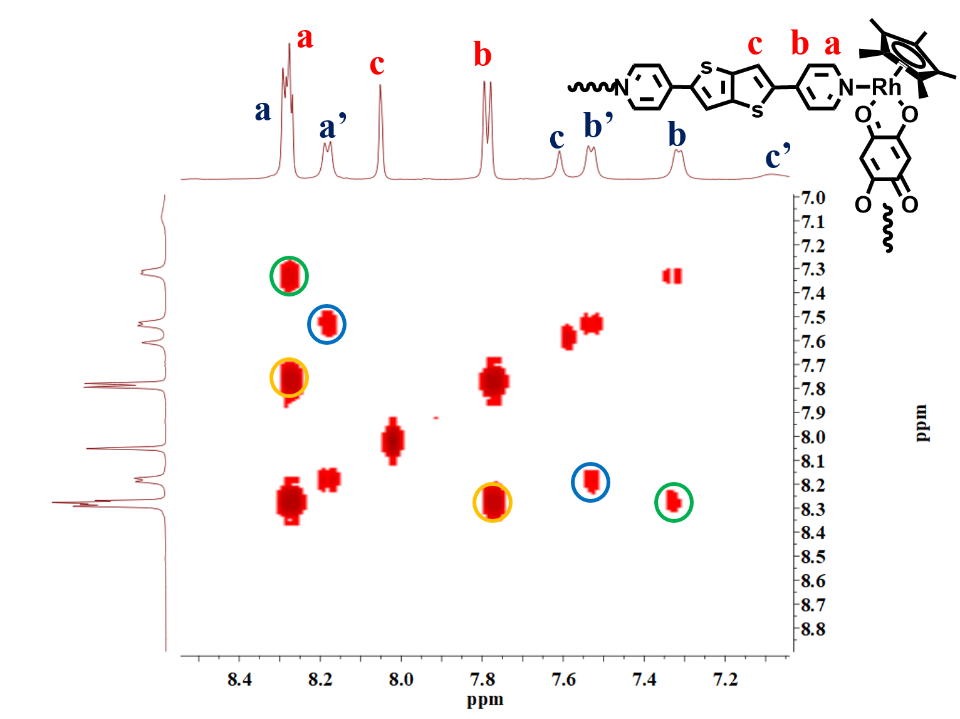


**Figure S13**. Partial ^1^H-^1^H COSY NMR spectrum of **3a** + **3a-IL** (CD_3_OD [5.0mM], 298K, 400 MHz).


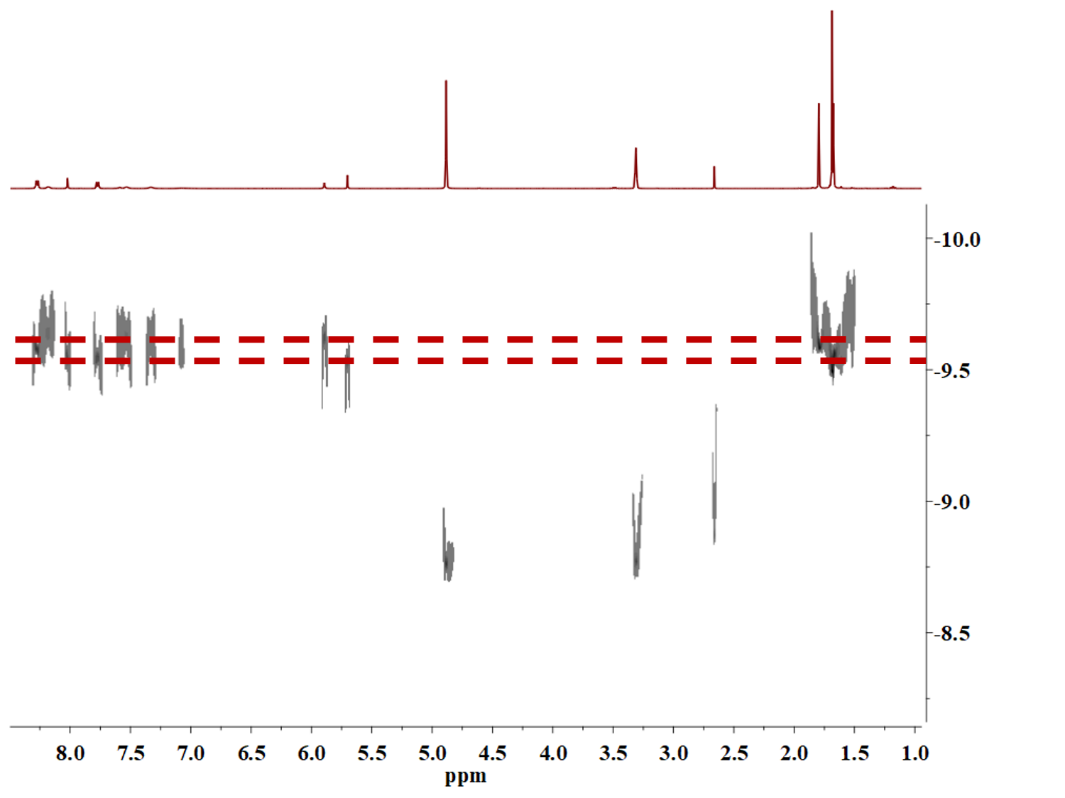


**Figure S14**. ^1^H DOSY NMR spectrum of **3a** + **3a-IL** (CD_3_OD [5.0mM], 298K, 400 MHz).


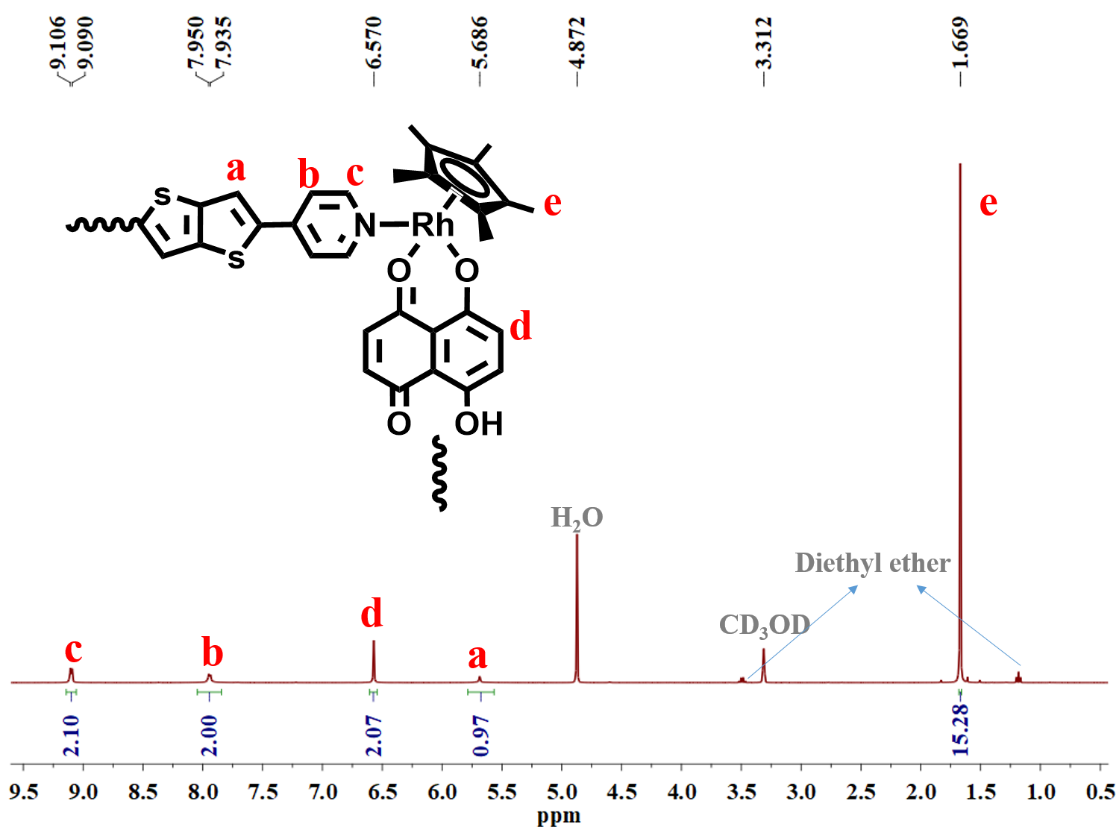


**Figure S15**. ^1^H NMR spectrum of **5-BRs** (CD_3_OD, [5.0mM], 298K, 400 MHz).


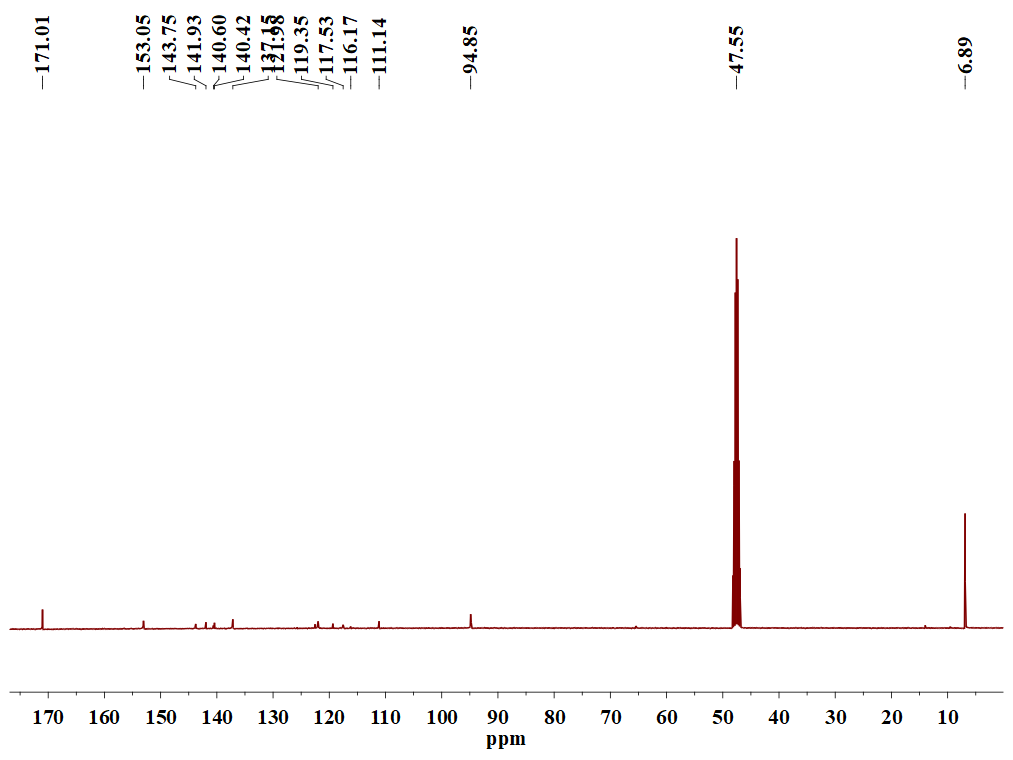


**Figure S16**. ^13^C NMR spectrum of **5-BRs** (CD_3_OD, [5.0mM], 298K, 400 MHz).


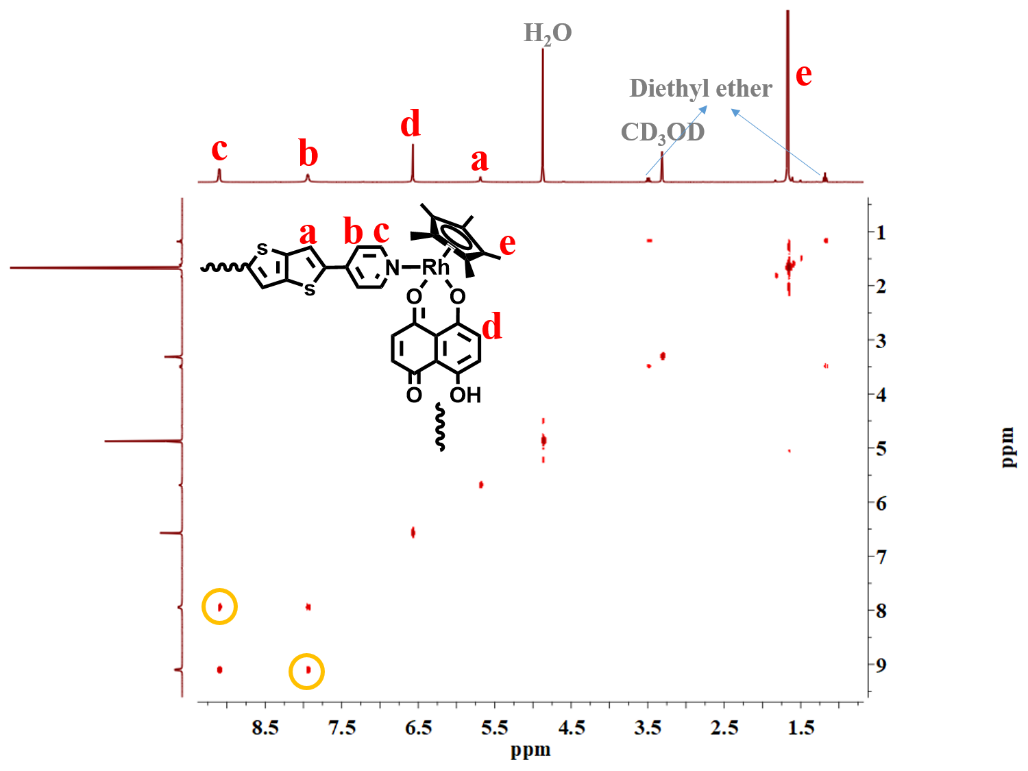


**Figure S17**. ^1^H-^1^H COSY NMR spectrum of **5-BRs** (CD_3_OD [5.0mM], 298K, 400 MHz).


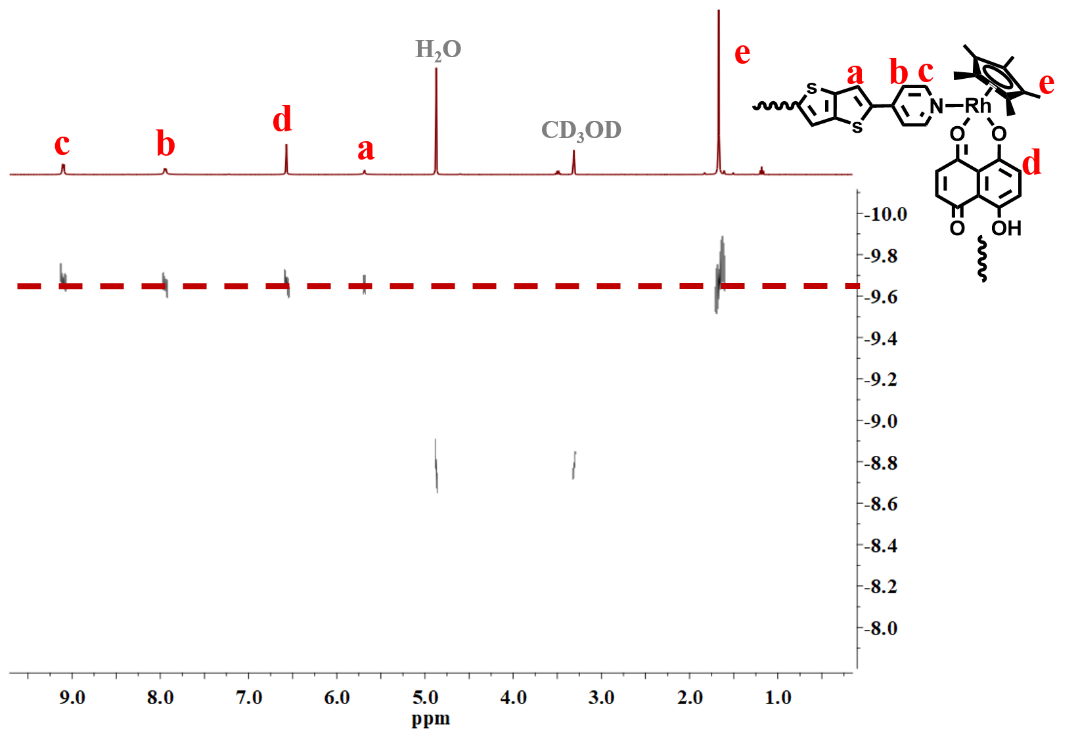


**Figure S18**. ^1^H DOSY NMR spectrum of **5-BRs** (CD_3_OD [5.0mM], 298K, 400 MHz).


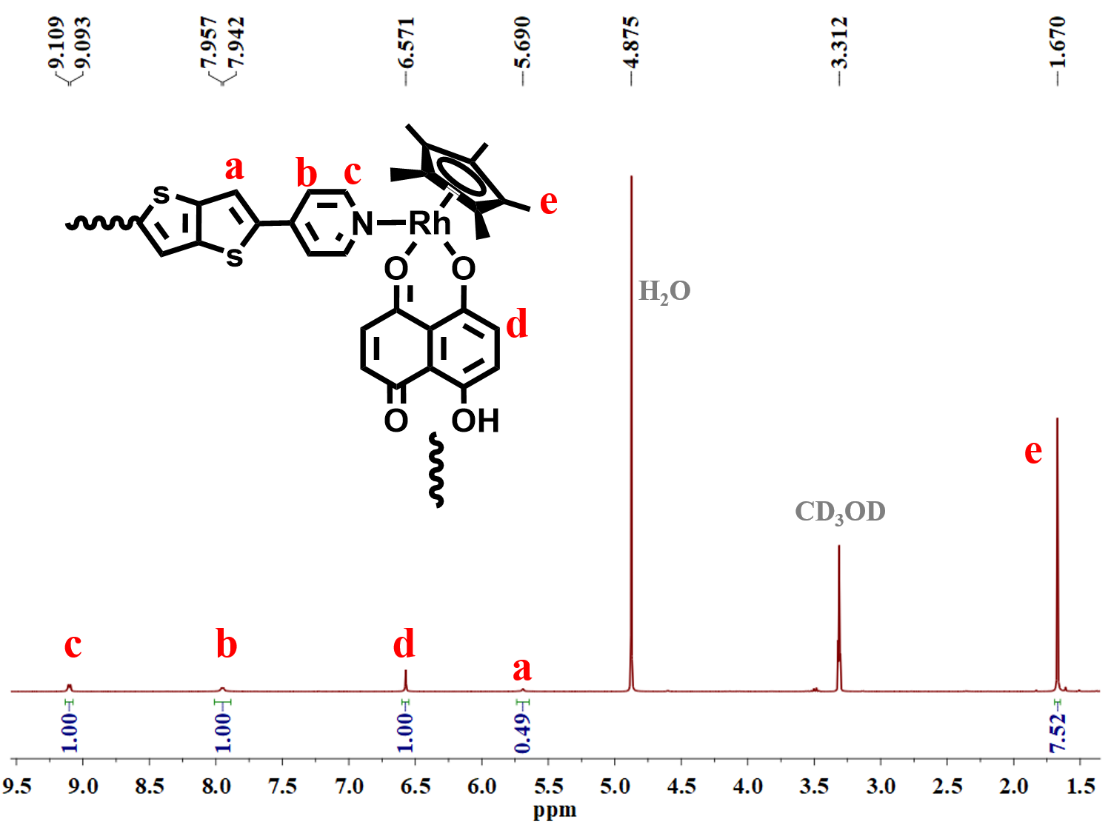


**Figure S19**. ^1^H NMR spectrum of **5-BRs** (CD_3_OD, [0.5mM], 298K, 400 MHz).


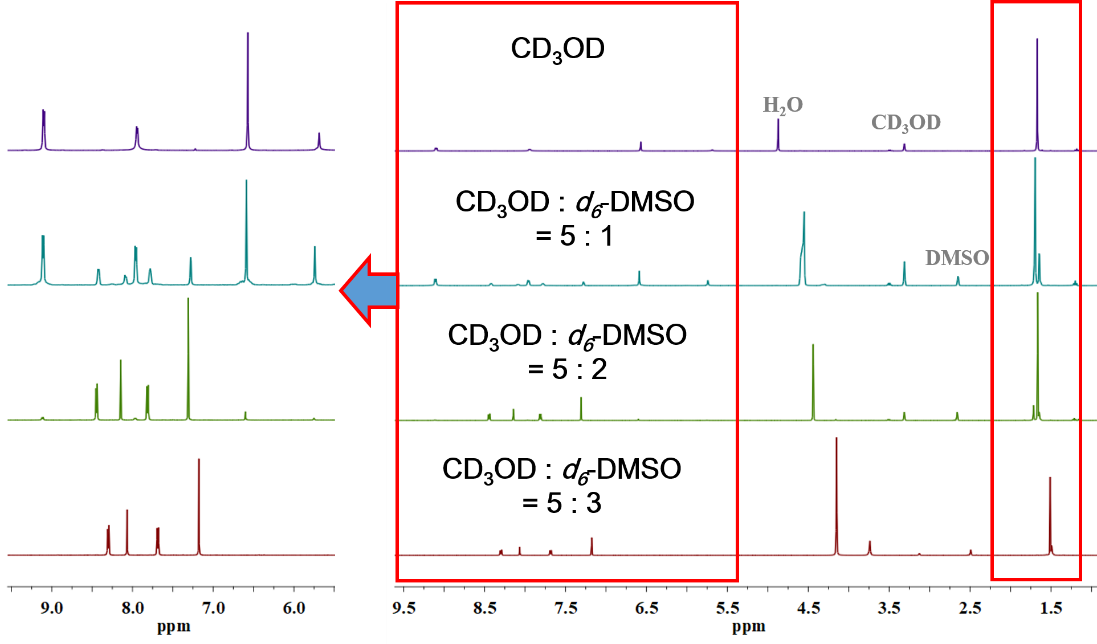


**Figure S20**. ^1^H NMR spectrum of **5-BRs** in CD_3_OD ([5.0 mM], 298K, 400 MHz), **5** + **5-BRs** in CD_3_OD and *d*_6_-DMSO (CD_3_OD: *d*_6_-DMSO = 5:1 [5.0mM], 298K, 400 MHz), **5** + **5-BRs** in CD_3_OD and *d*_6_-DMSO (CD_3_OD: *d*_6_-DMSO = 5:2 [5.0mM], 298K, 400 MHz), **5** in CD_3_OD and *d*_6_-DMSO (CD_3_OD: *d*_6_-DMSO = 5:3 [5.0mM], 298K, 400 MHz).


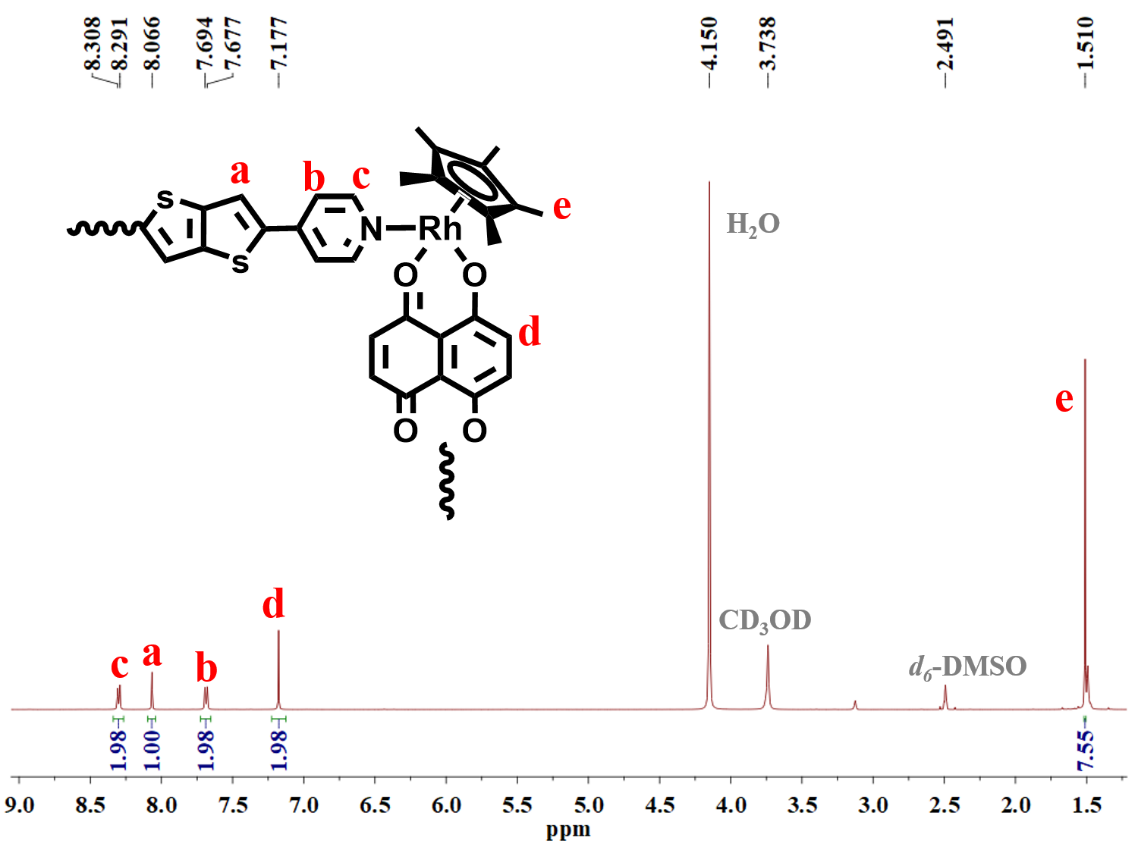


**Figure S21**. ^1^H NMR spectrum of **5** in CD_3_OD and *d*_6_-DMSO (CD_3_OD: *d*_6_-DMSO = 5:3 [5.0mM], 298K, 400 MHz).


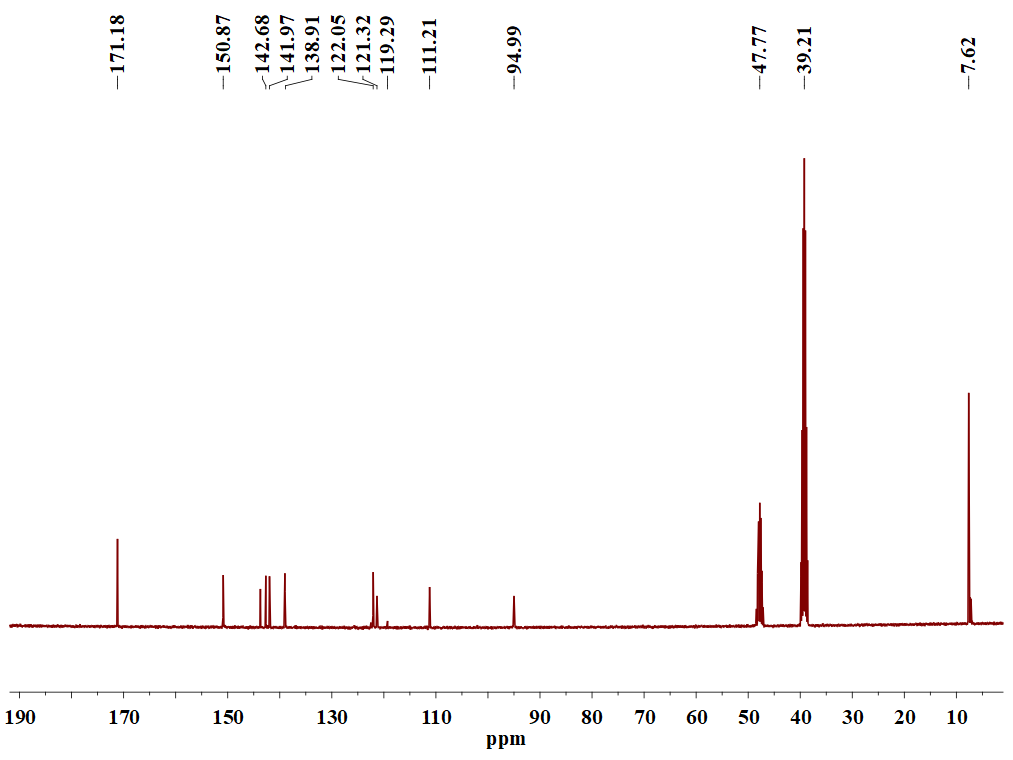


**Figure S22**. ^13^C NMR spectrum of **5** in CD_3_OD and *d*_6_-DMSO (CD_3_OD: *d*_6_-DMSO = 5:3 [5.0mM], 298K, 400 MHz).


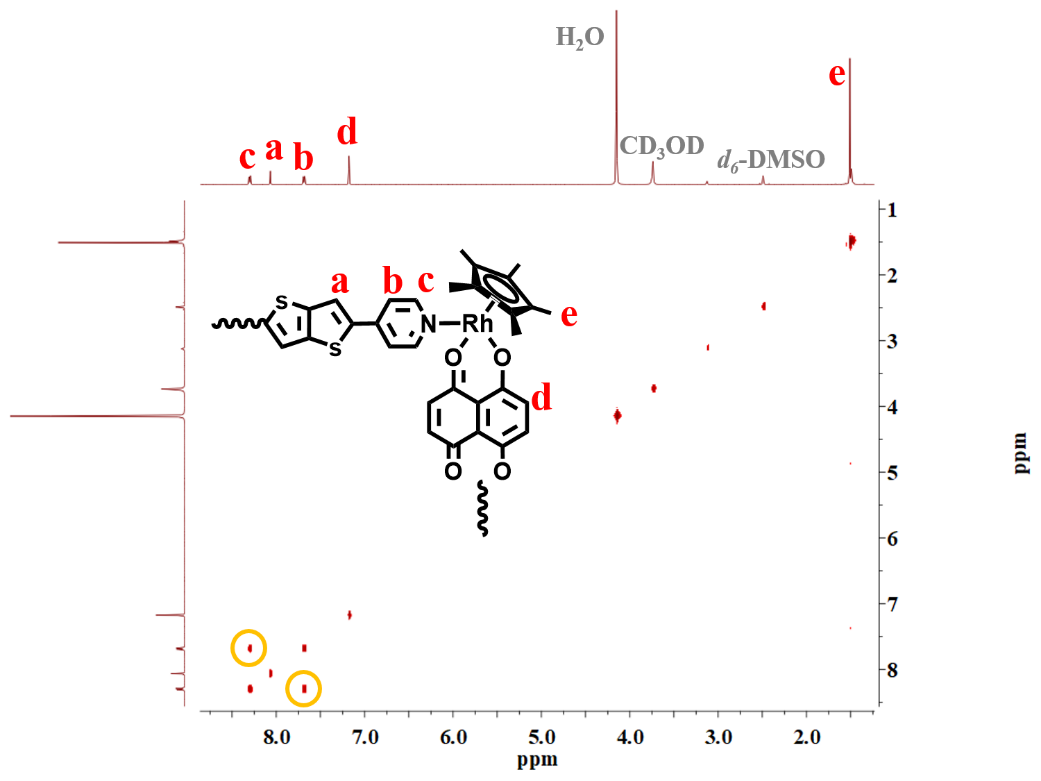


**Figure S23**. ^1^H-^1^H COSY NMR spectrum of **5** in CD_3_OD and *d*_6_-DMSO (CD_3_OD: *d*_6_-DMSO = 5:3 [5.0mM], 298K, 400 MHz).


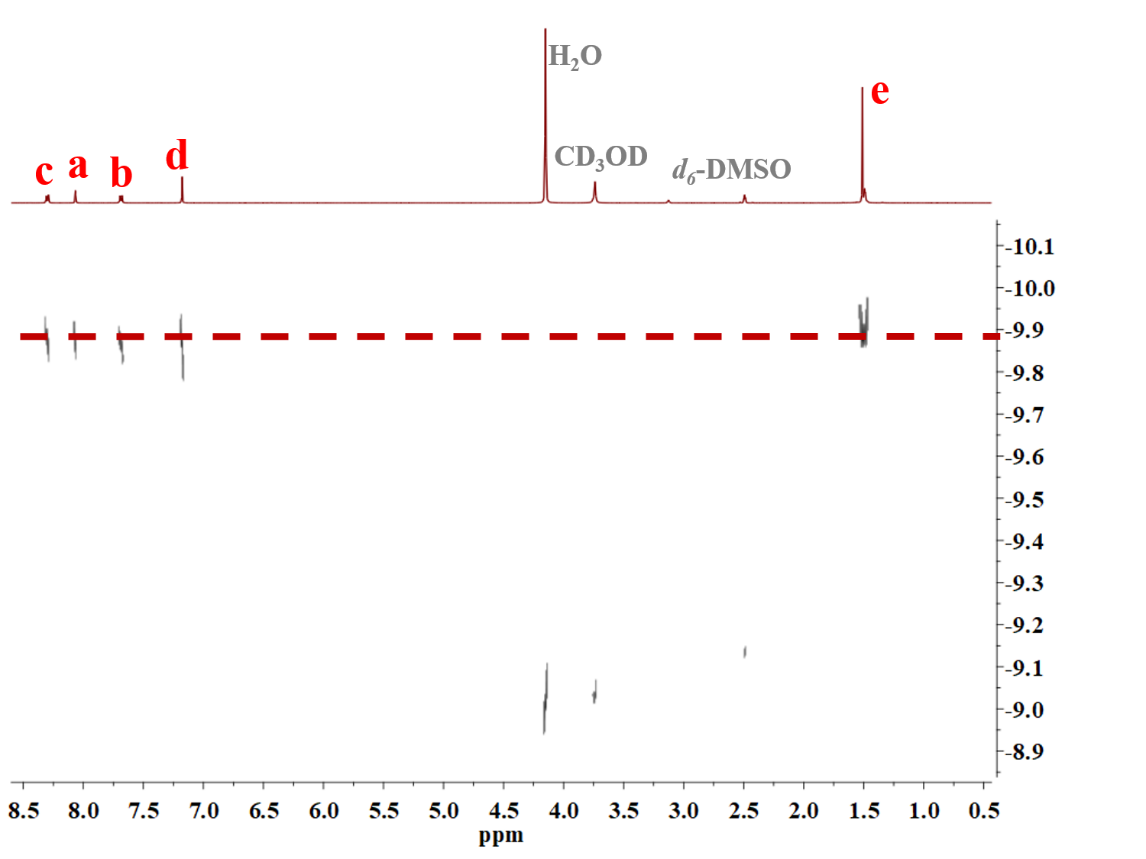


**Figure S24**. ^1^H DOSY NMR spectrum of **5** in CD_3_OD and *d*_6_-DMSO (CD_3_OD: *d*_6_-DMSO = 5:3 [5.0mM], 298K, 400 MHz).


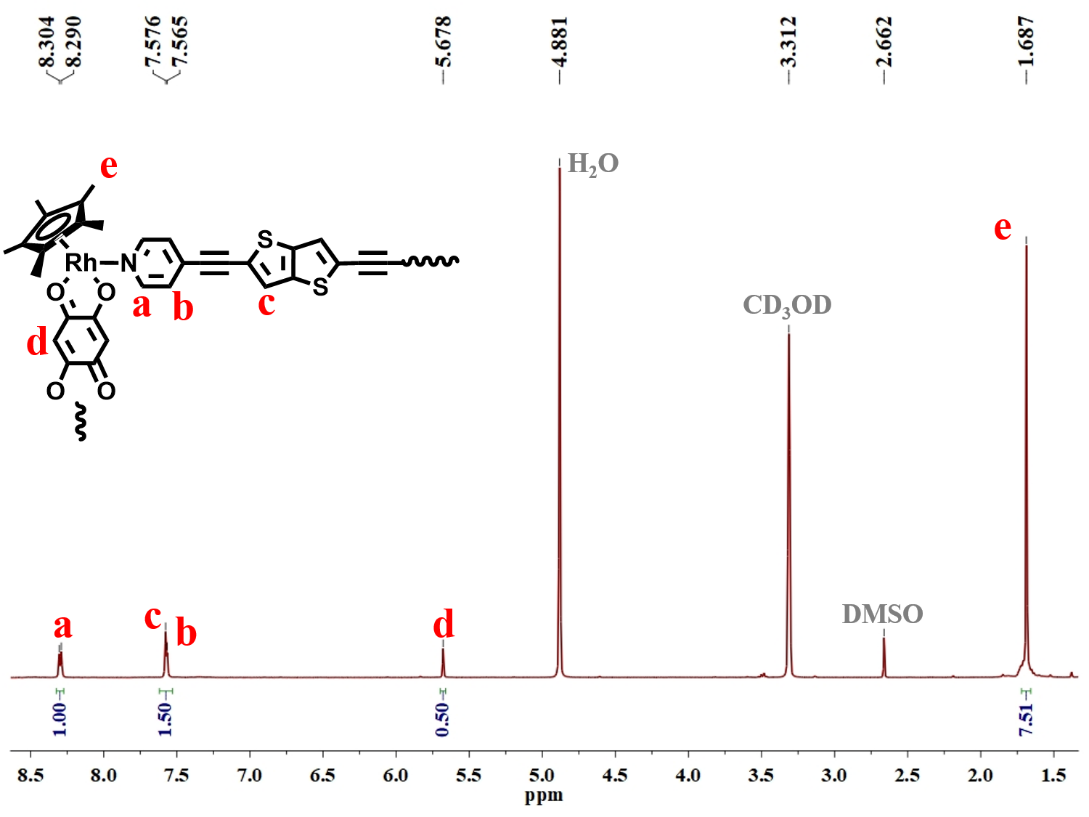


**Figure S25**. ^1^H NMR spectrum of **6** (CD_3_OD, [1.0mM], 298K, 400 MHz).


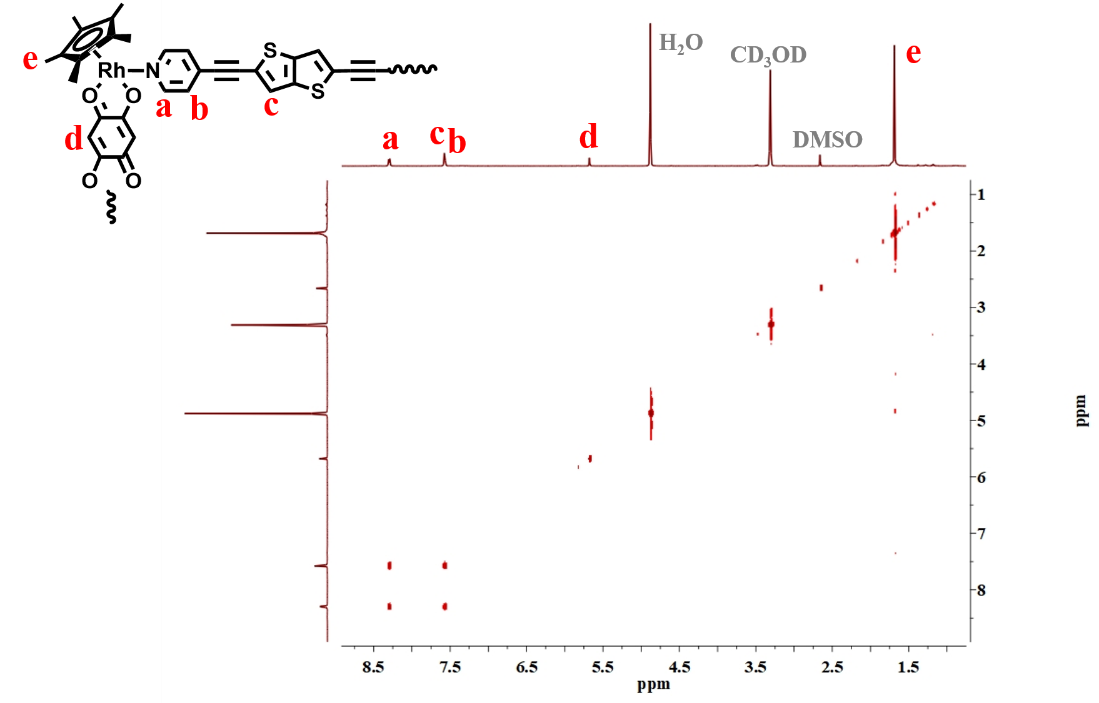


**Figure S26**. ^1^H-^1^H COSY NMR spectrum of **6** (CD_3_OD, [1.0mM], 298K, 400 MHz).


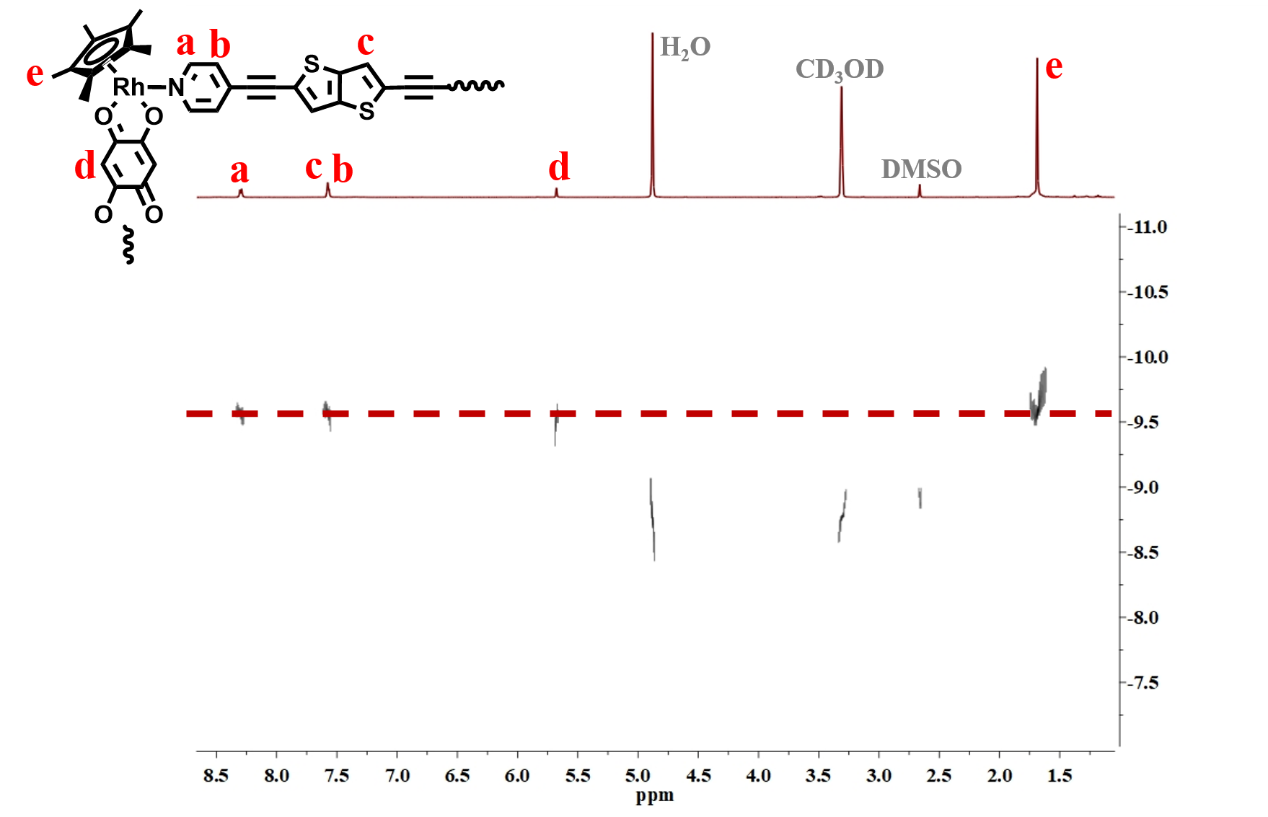


**Figure S27**. ^1^H DOSY NMR spectrum of **6** in (CD_3_OD, [1.0mM], 298K, 400 MHz).


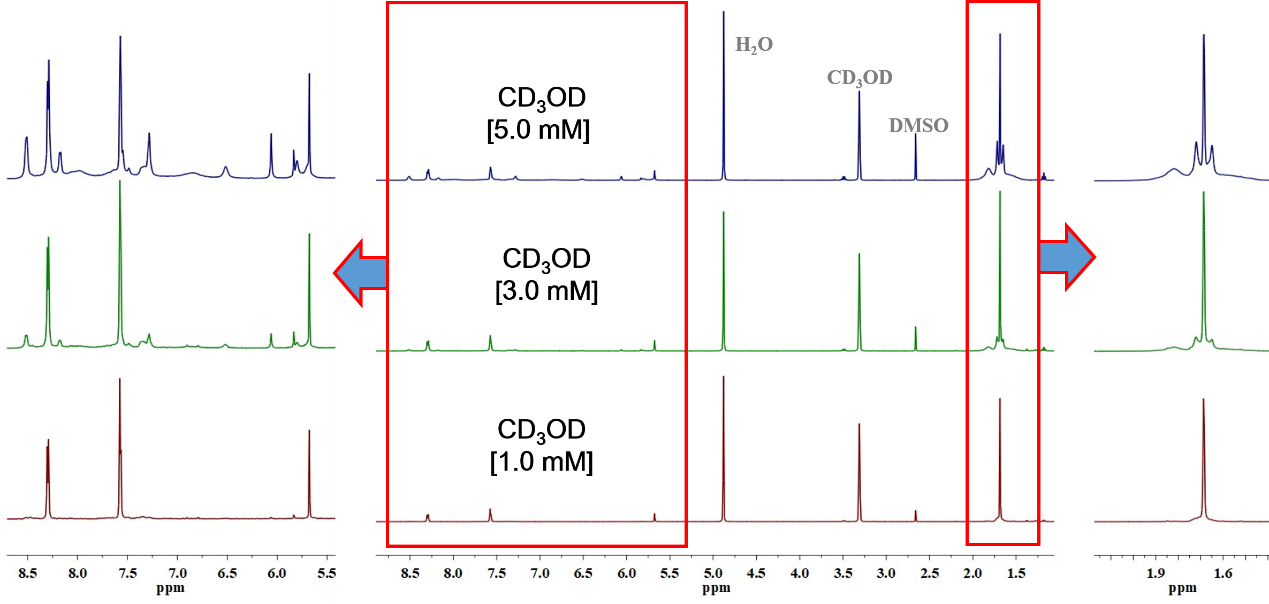


**Figure S28**. ^1^H NMR spectrum of showing transformation from **6** to [**6**+ **6-IL**] up increasing the concentration from 1.0 mM to 5.0 mM (CD_3_OD, [1.0 mM], [3.0 mM], [5.0 mM], 298K, 400 MHz).


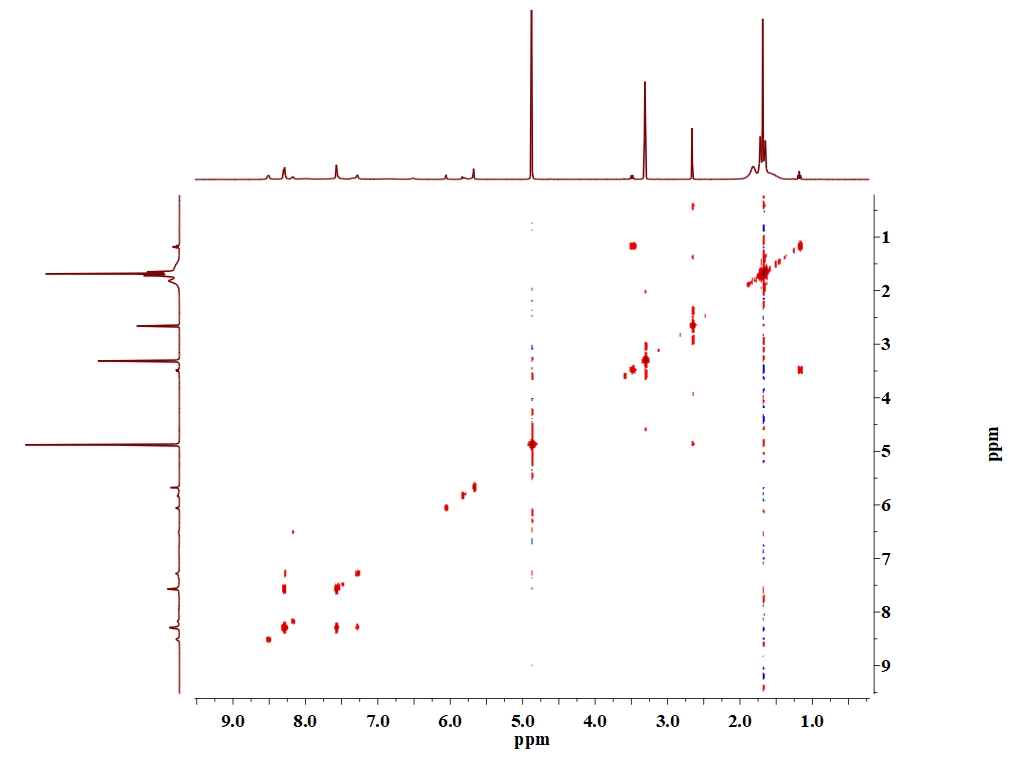


**Figure S29**. ^1^H-^1^H COSY NMR spectrum of **6** + **6-IL** (CD_3_OD, [5.0mM], 298K, 400 MHz).


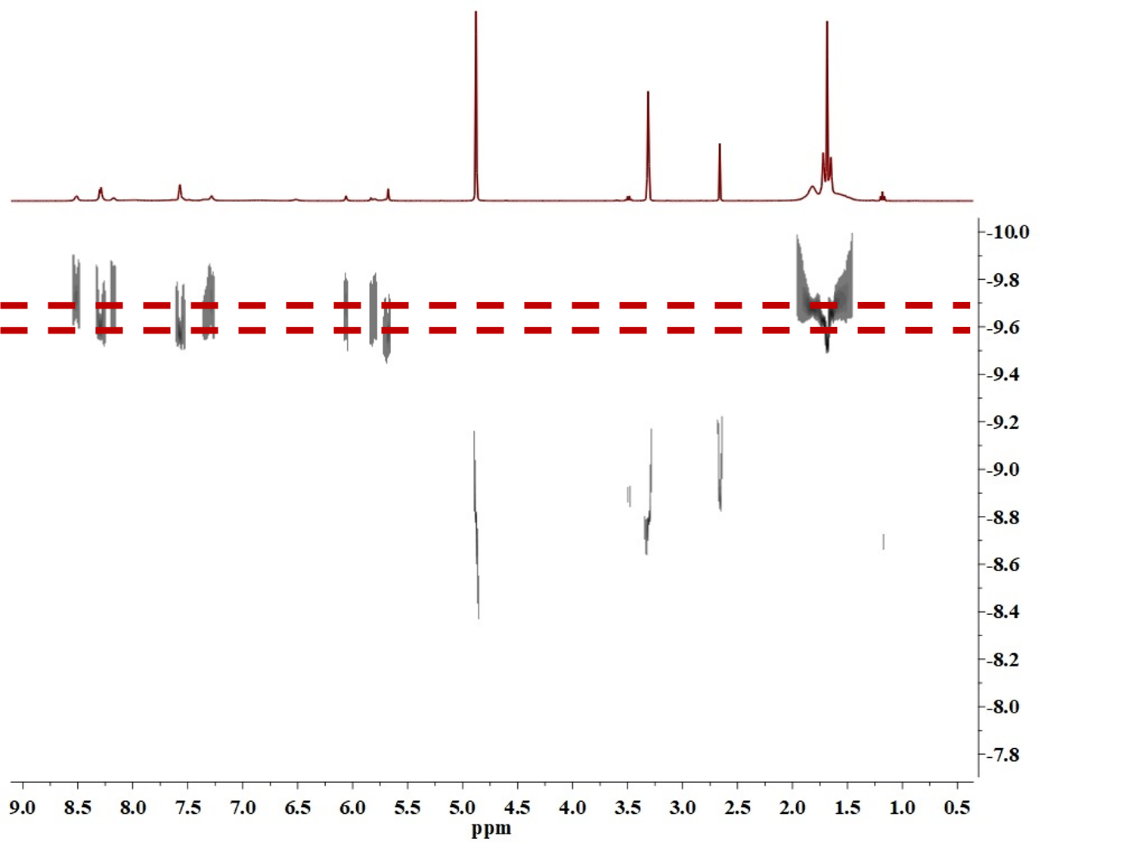


**Figure S30**. ^1^H DOSY NMR spectrum of **6** and **6-IL** (CD_3_OD, [5.0mM], 298K, 400 MHz).


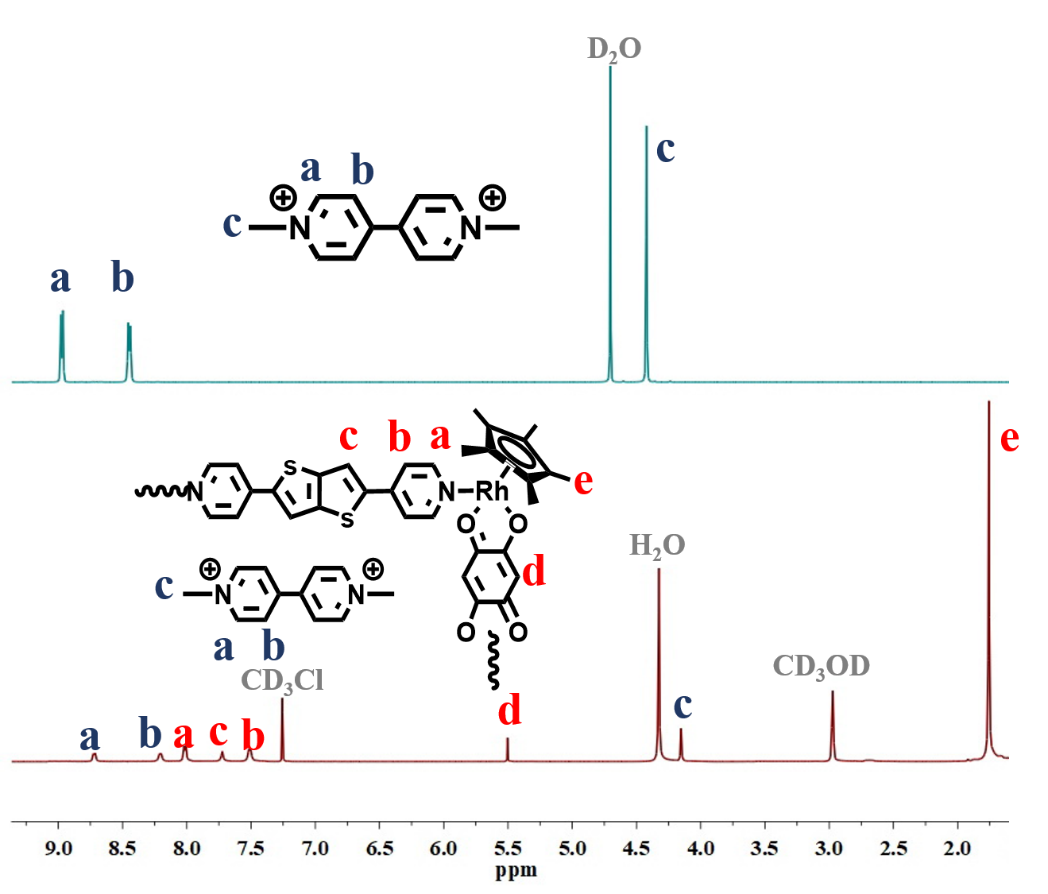


**Figure S31**. ^1^H NMR spectrum of methylviologen ditriflate in D_2_O ([8.0mM], 298K, 400 MHz) and **3a** encapsulated methylviologen ditriflate in CD_3_OD and CD_3_Cl (CD_3_OD: CD_3_Cl = 1:1 [3.0mM], 298K, 400 MHz).


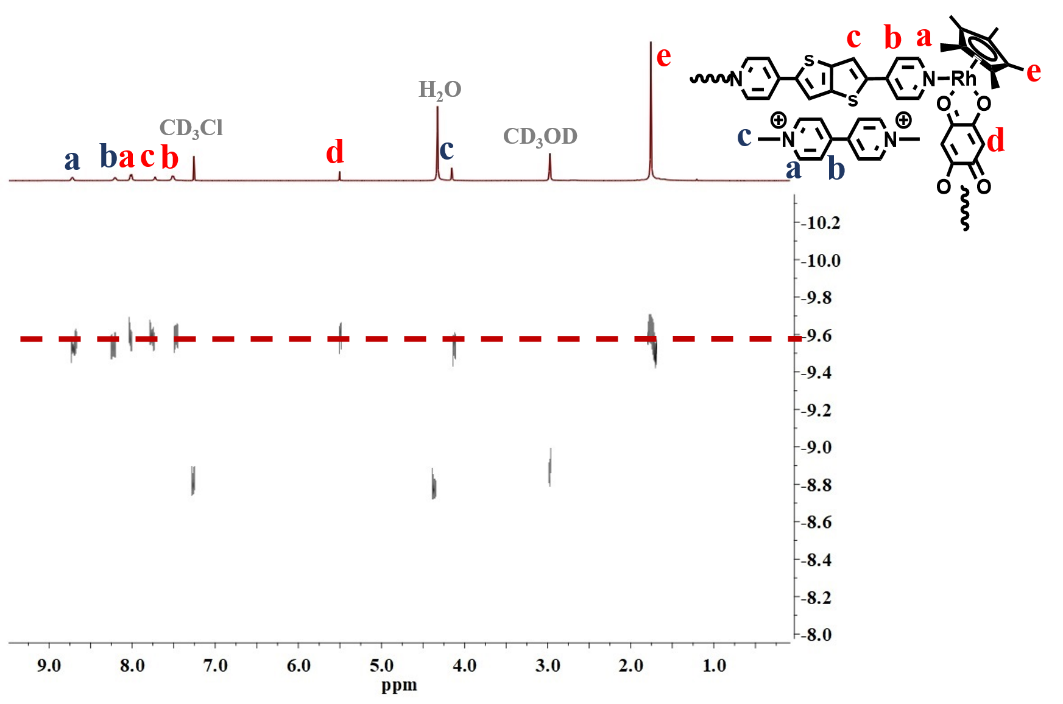


**Figure S32**. ^1^H DOSY NMR spectrum of **3a** encapsulated methylviologen ditriflate in CD_3_OD and CD_3_Cl (CD_3_OD: CD_3_Cl = 1:1 [3.0mM], 298K, 400 MHz).


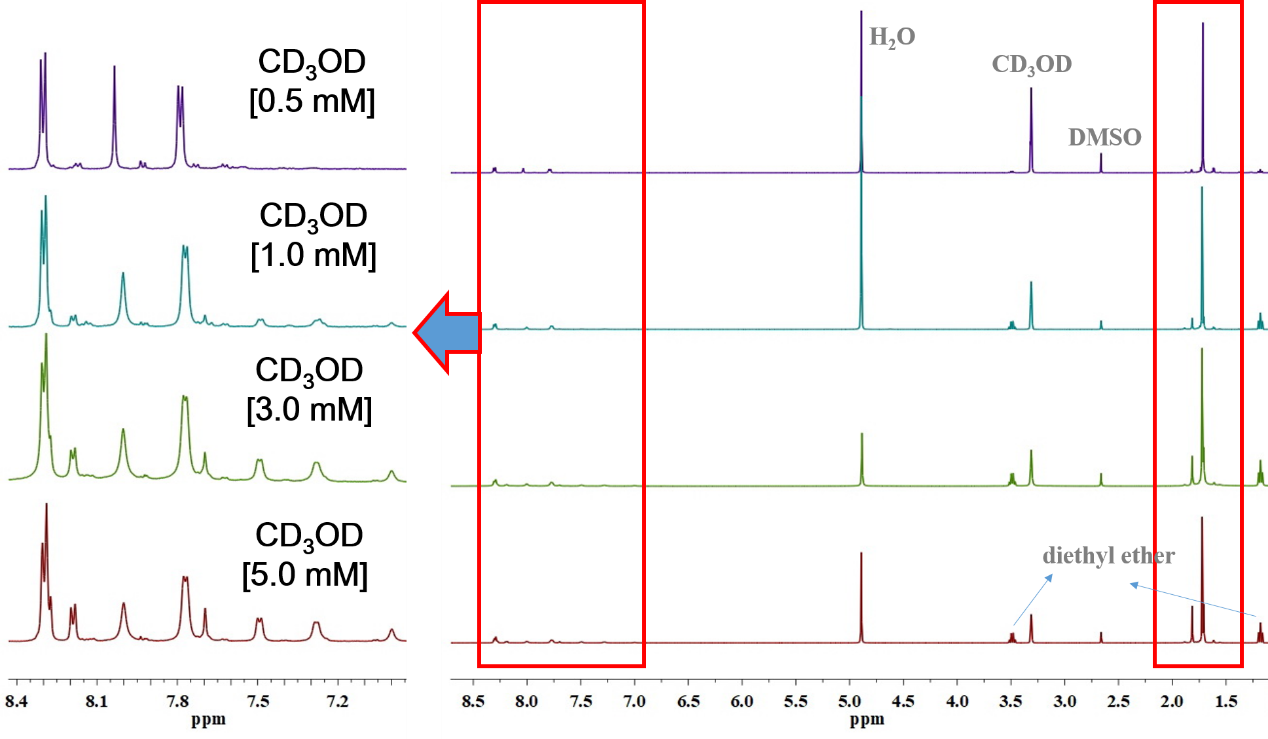


**Figure S33**. ^1^H NMR spectrum of showing transformation from **3b** to [**3b** + **3b-IL**] up increasing the concentration from 0.5 mM to 5.0 mM ([0.5 mM], [1.0 mM], [3.0 mM], [5.0 mM], 298K, 400 MHz).


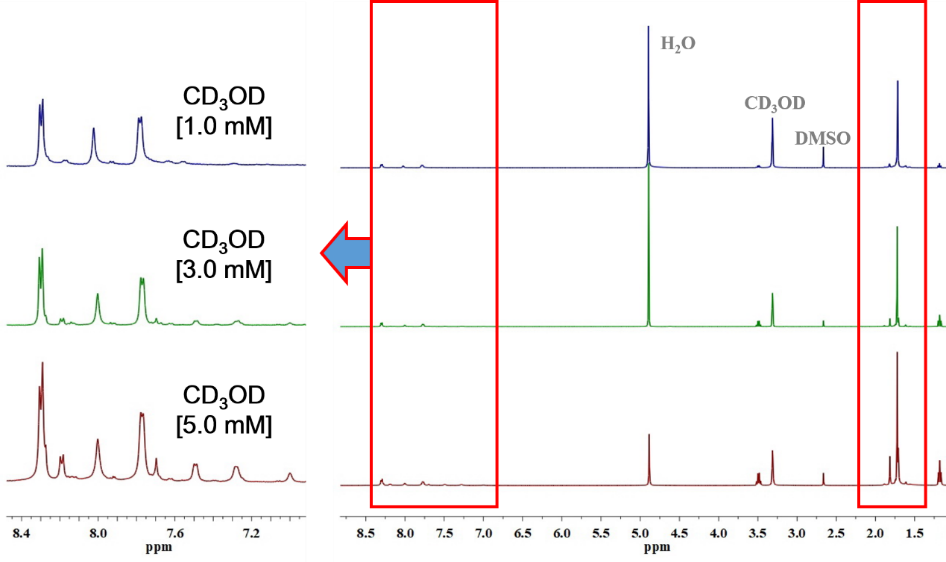


**Figure S34**. ^1^H NMR spectrum of showing transformation from **3c** to [**3c** + **3c-IL**] up increasing the concentration from 1.0 mM to 5.0 mM ([1.0 mM], [3.0 mM], [5.0 mM], 298K, 400 MHz).


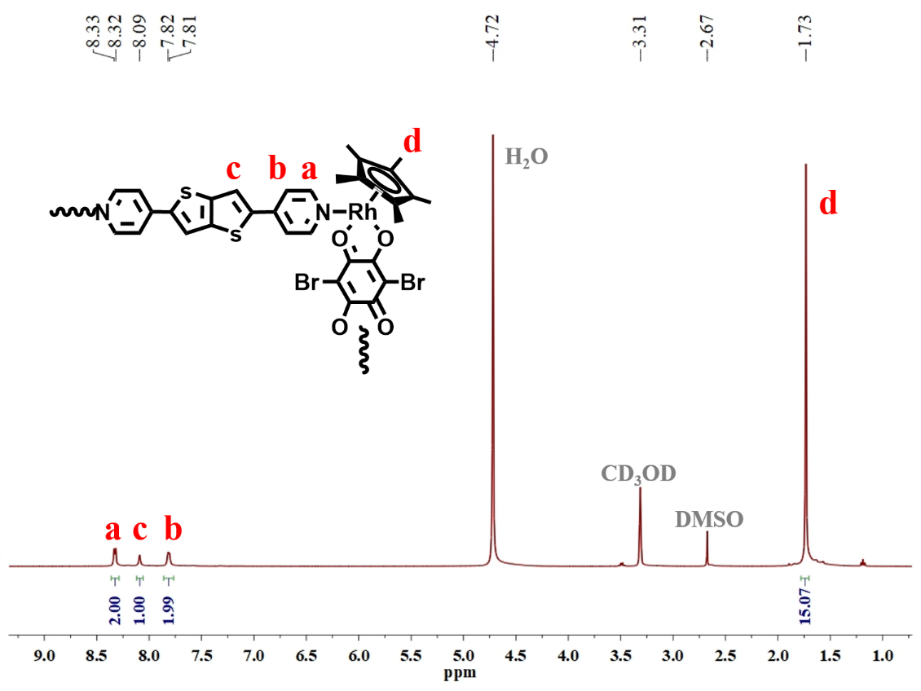


**Figure S35**. ^1^H NMR spectrum of **3d** in CD_3_OD (3.0 mM, 298K, 400 MHz).

**
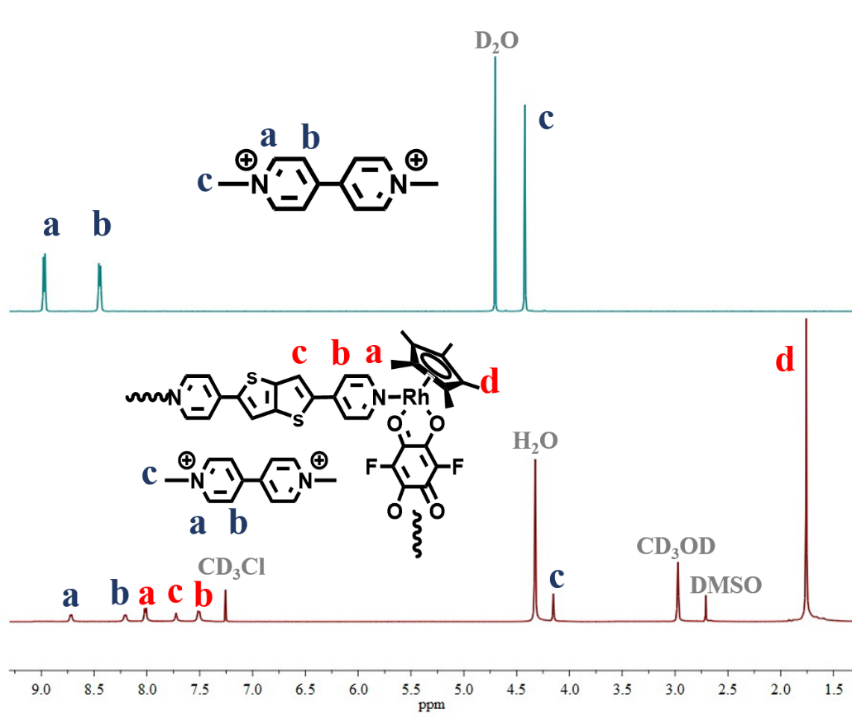
**

**Figure S36**. ^1^H NMR spectrum of methylviologen ditriflate in D_2_O ([8.0mM], 298K, 400 MHz) and **3b** encapsulated methylviologen ditriflate in CD_3_OD and CD_3_Cl (CD_3_OD: CD_3_Cl = 1:1 [3.0mM], 298K, 400 MHz).


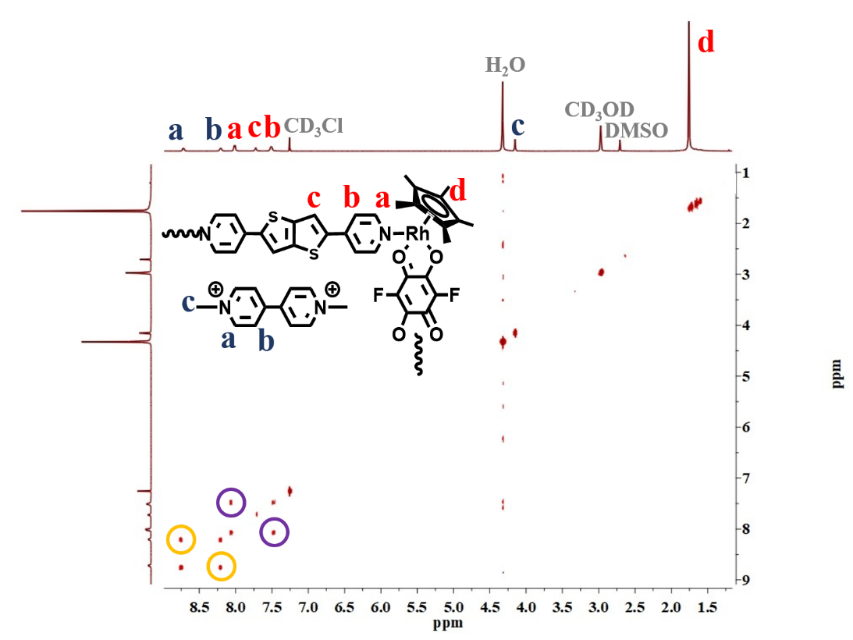


**Figure S37**. ^1^H-^1^H COSY NMR spectrum of **3b** encapsulated methylviologen ditriflate in CD_3_OD and CD_3_Cl (CD_3_OD: CD_3_Cl = 1:1 [3.0mM] , 298K, 400 MHz).


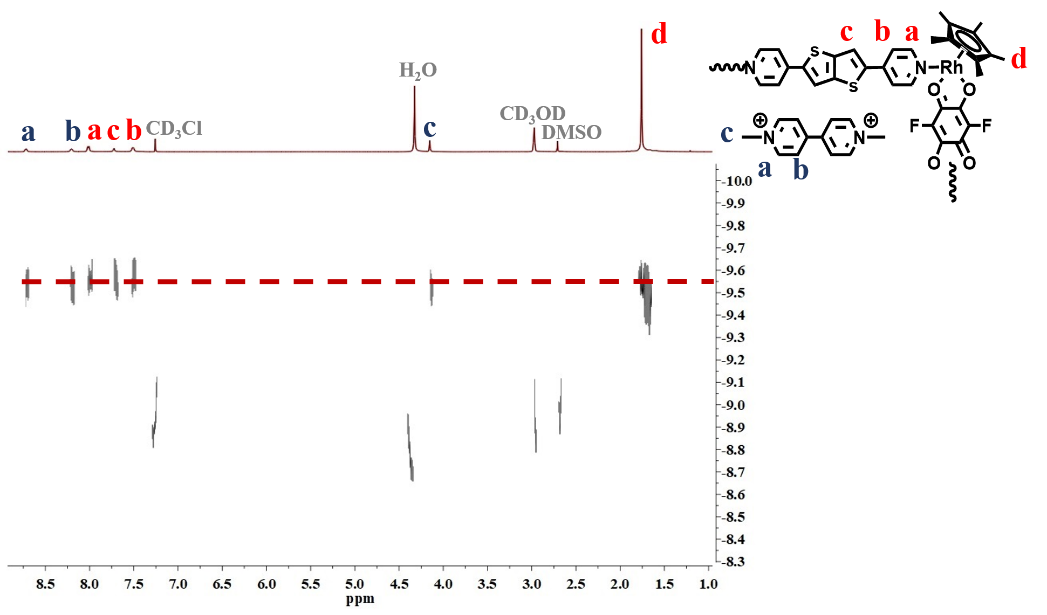


**Figure S38**. ^1^H DOSY NMR spectrum of **3b** encapsulated methylviologen ditriflate in CD_3_OD and CD_3_Cl (CD_3_OD: CD_3_Cl = 1:1 [3.0mM], 298K, 400 MHz).


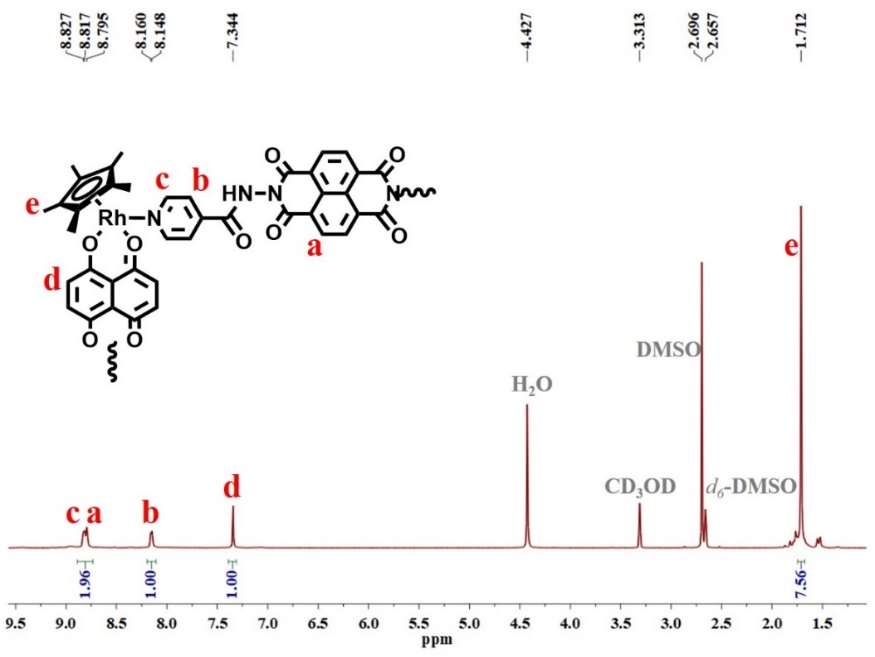


**Figure S39**. ^1^H NMR spectrum of **7** in CD_3_OD and *d_6_*-DMSO (CD_3_OD: *d_6_*-DMSO = 5:1 [5.0mM], 298K, 400 MHz).

**
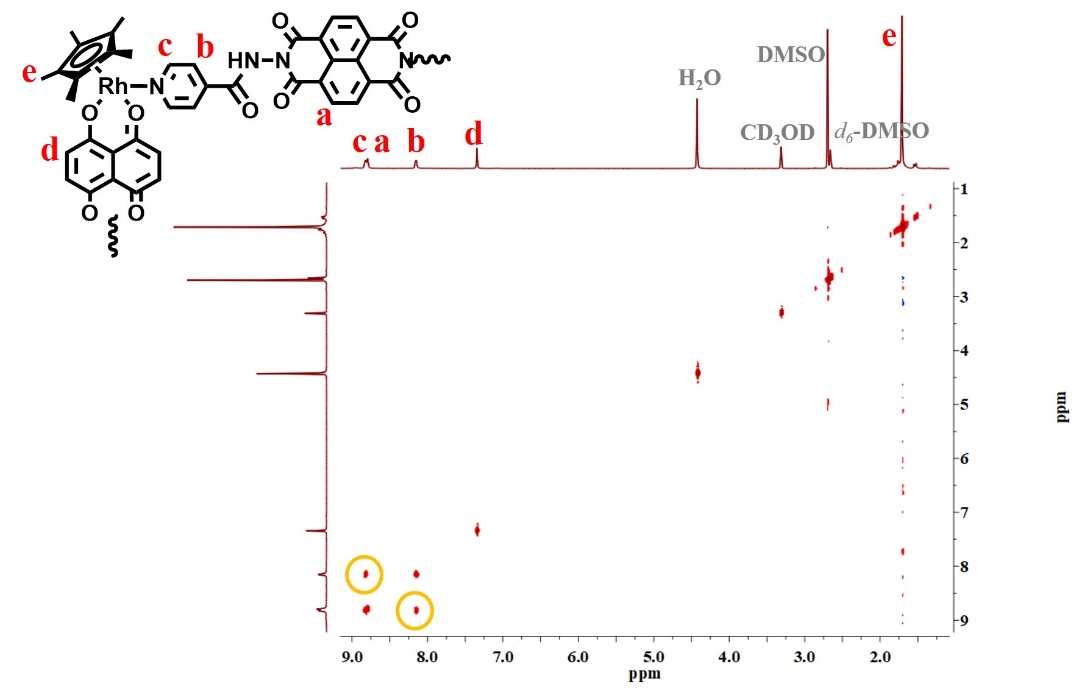
**

**Figure S40**. ^1^H-^1^H COSY NMR spectrum of **7** in CD_3_OD and *d_6_*-DMSO (CD_3_OD: *d_6_*-DMSO = 5:1 [5.0mM], 298K, 400 MHz).


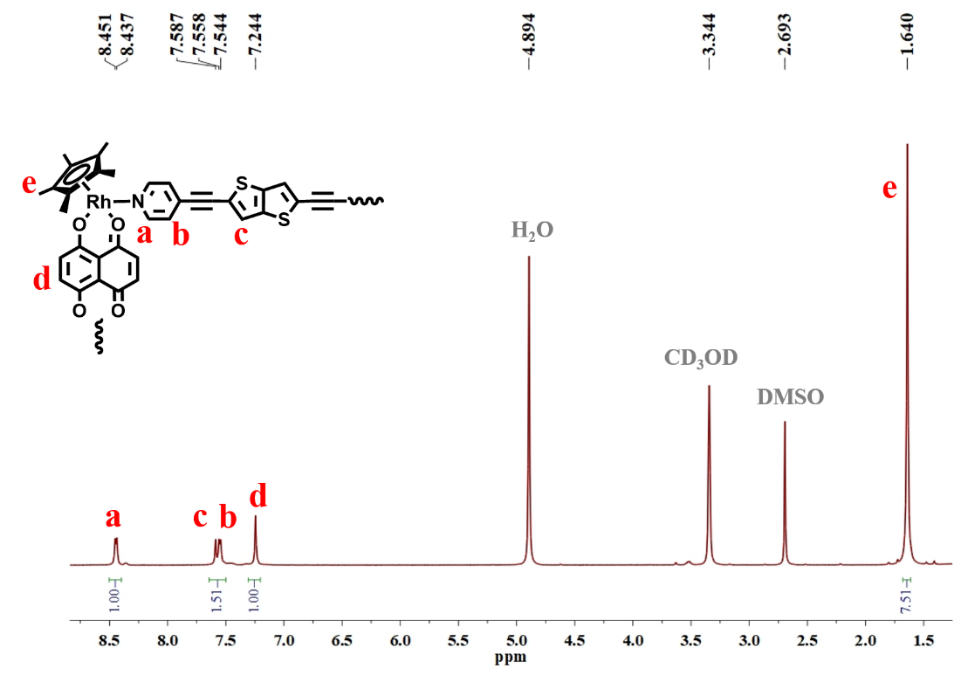


**Figure S41**. ^1^H NMR spectrum of **8** in supersaturated solution of CD_3_OD (5.0 mM, 298K, 400 MHz).

**
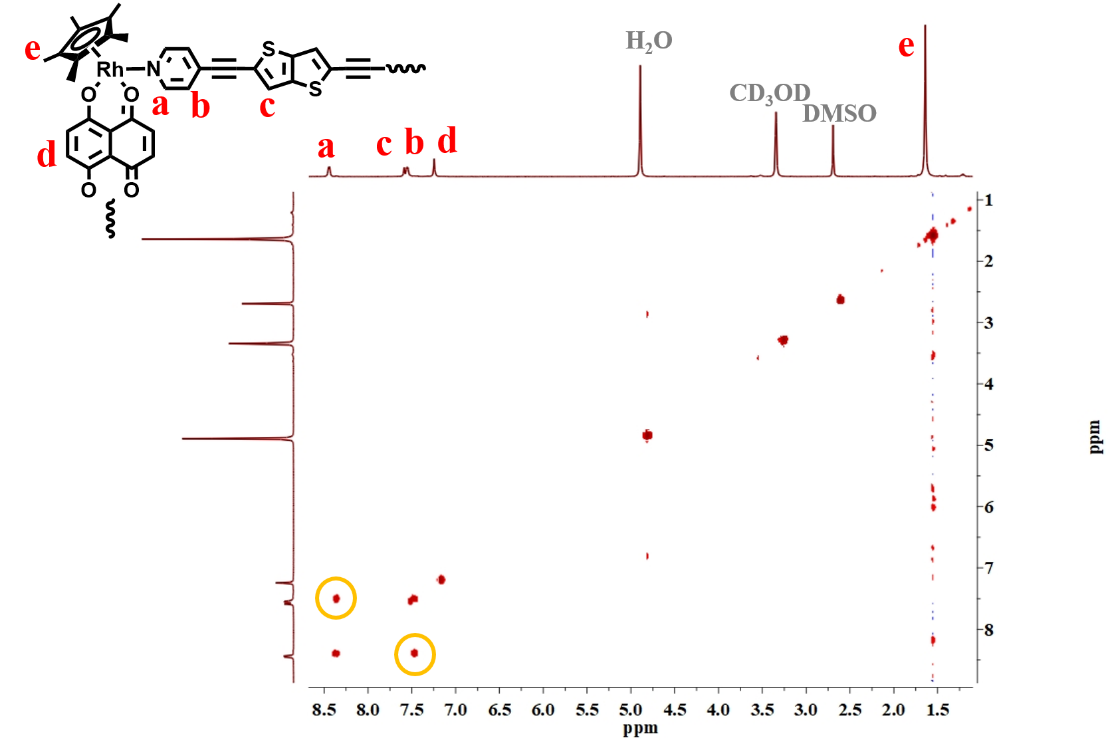
**

**Figure S42**. ^1^H-^1^H COSY NMR spectrum of **8** in supersaturated solution of CD_3_OD (5.0 mM, 298K, 400 MHz).

**
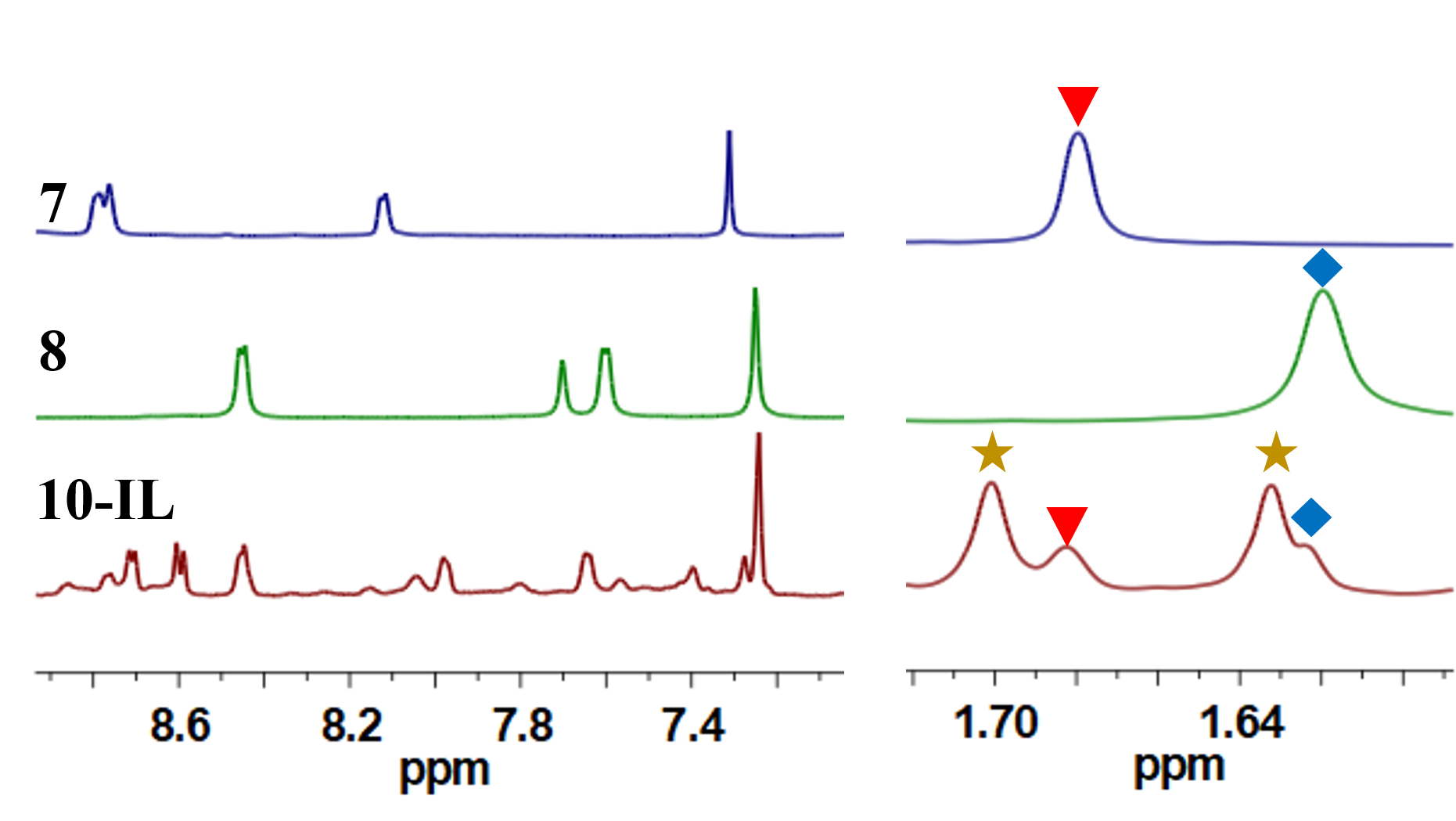
**

**Figure S43**. ^1^H NMR spectrum of **8** (
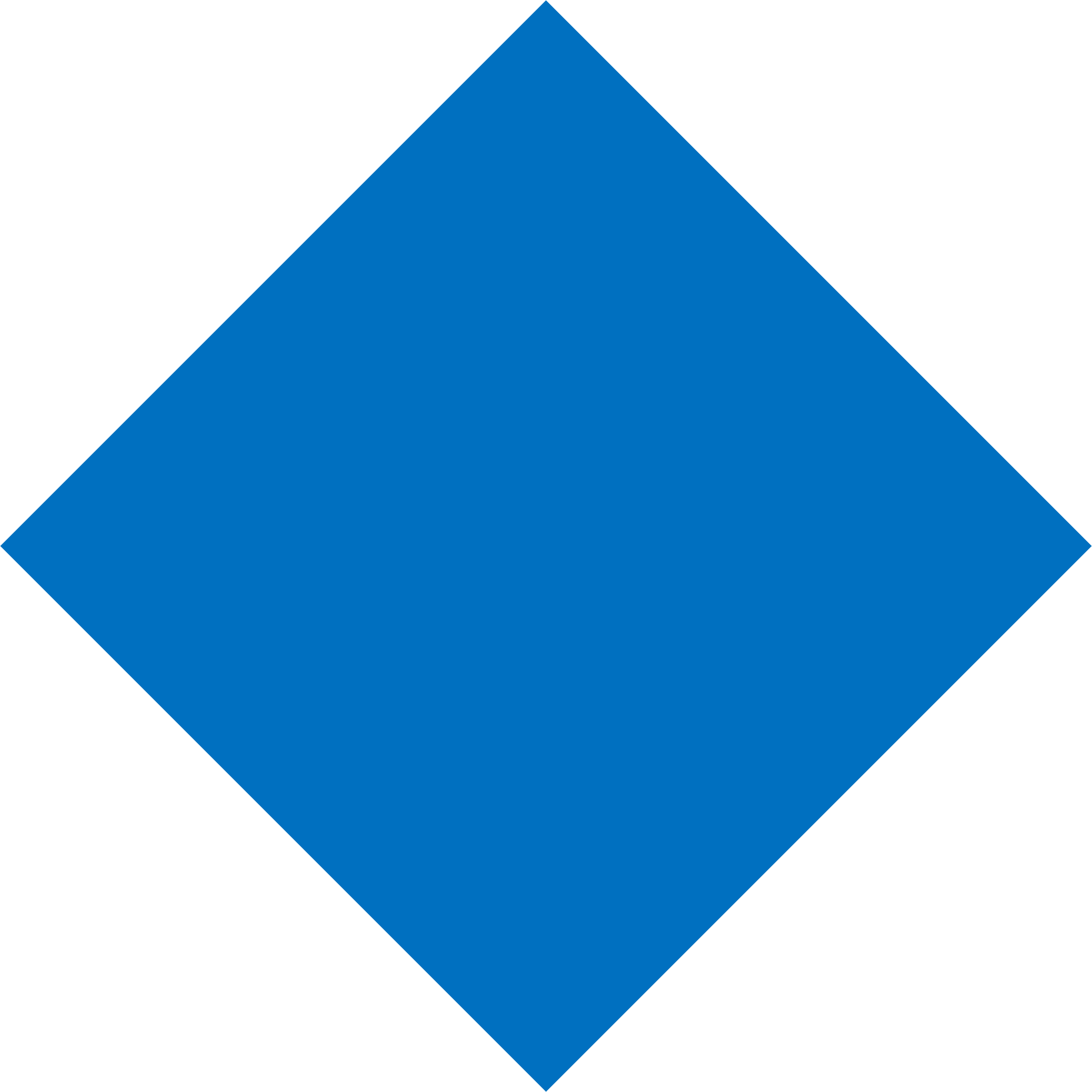
), **7** (
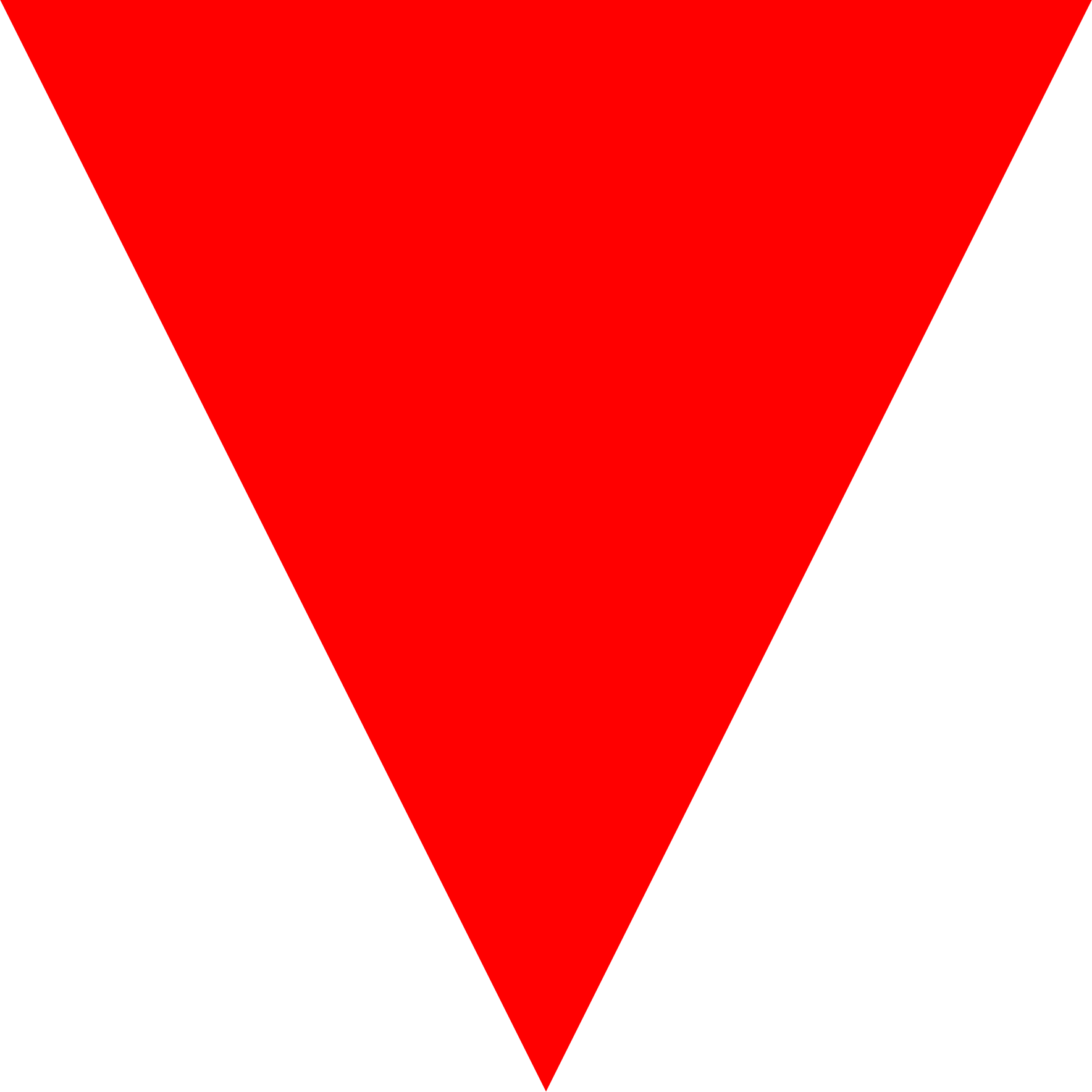
) and **10-IL** (
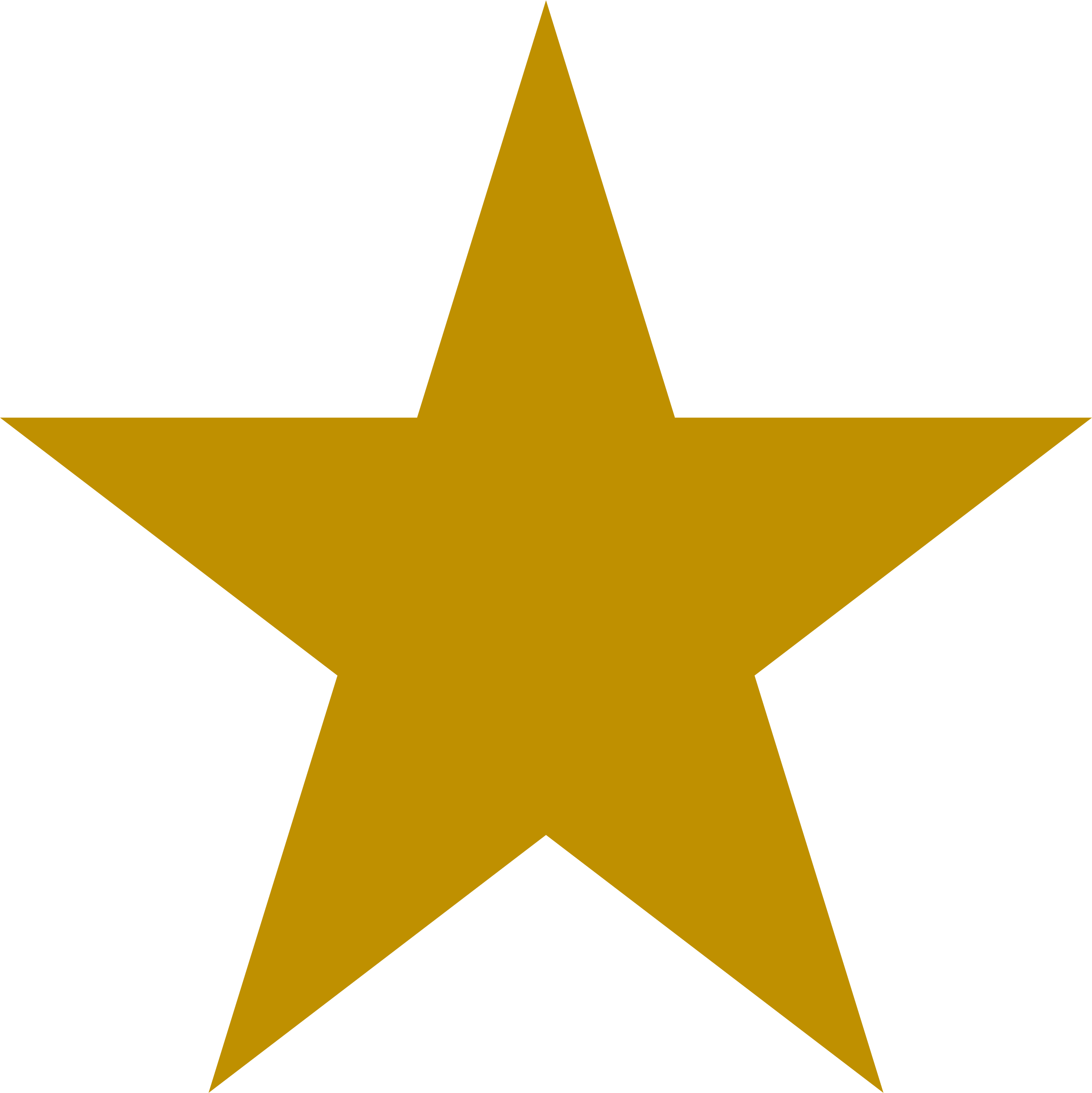
) in a CD_3_OD / *d_6_*-DMSO mixture (CD_3_OD: *d_6_*-DMSO = 5:1 [5.0 mM], 400 MHz);


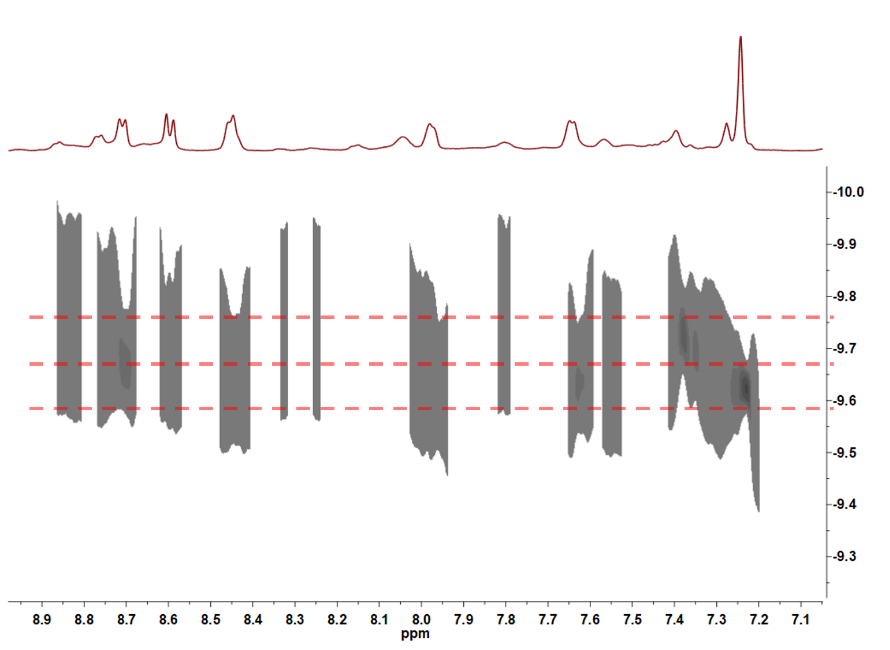


**Figure S44**. ^1^H DOSY spectrum of **8** + **7** + **10-IL** in CD_3_OD and *d_6_*-DMSO (CD_3_OD: *d_6_*-DMSO = 5:1 [5.0mM], 298K, 400 MHz).


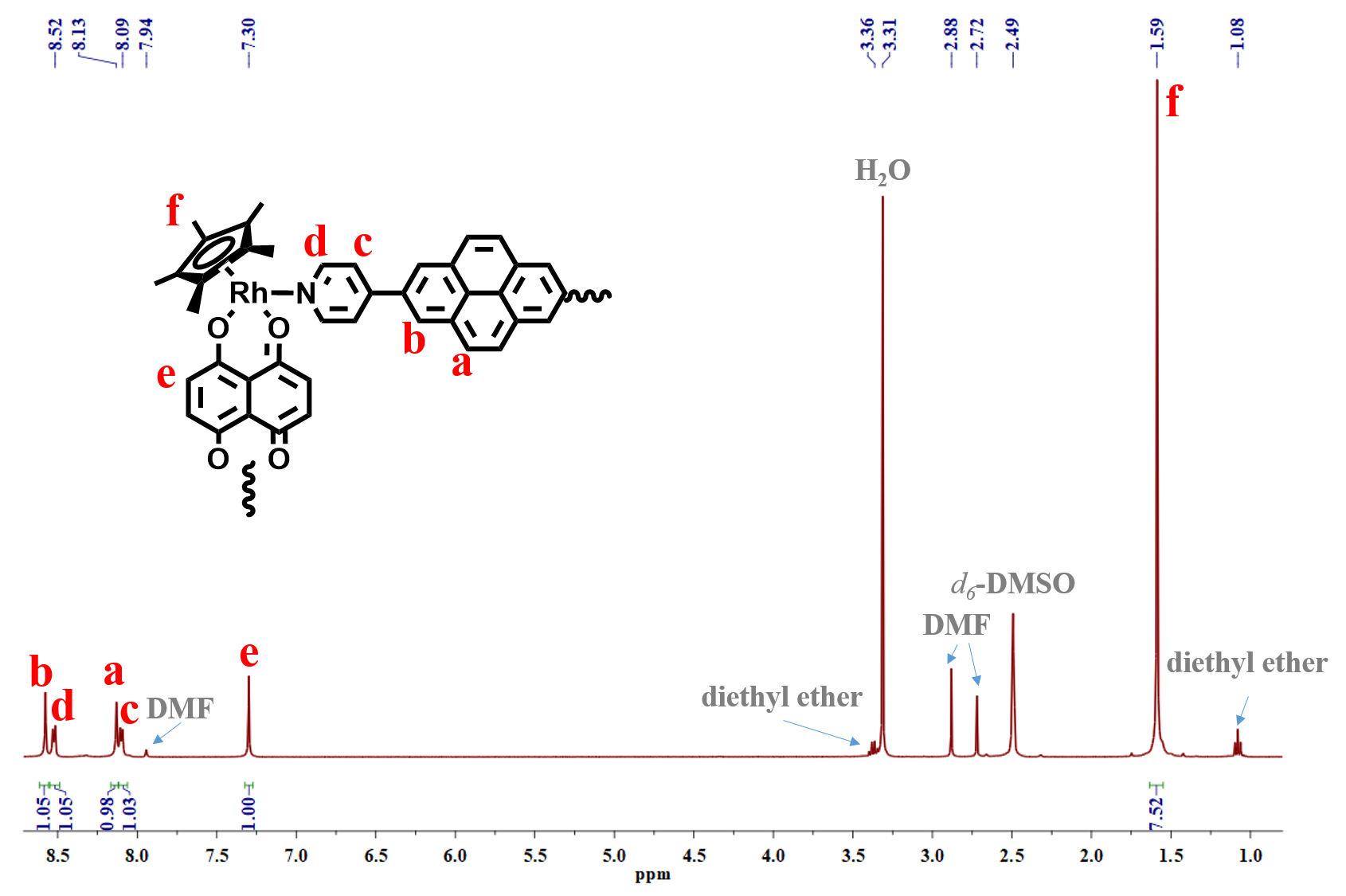


**Figure S45**. ^1^H NMR spectrum of **9** (*d_6_*-DMSO, [5.0 mM], 400 MHz). The solubility of **9** in methanol is very low, so ^1^H NMR spectrum of **9** is recorded by DMSO solution.

ESI-MS


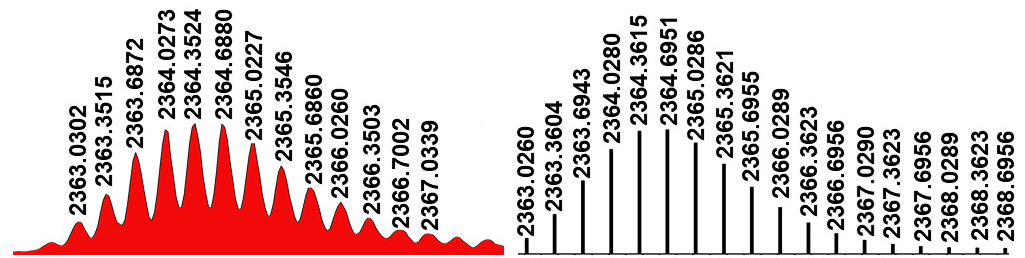


Figure S46. Experimental (right) and calculated (left) ESI-MS spectra of [5-BRs-3OTf]^3+^

^
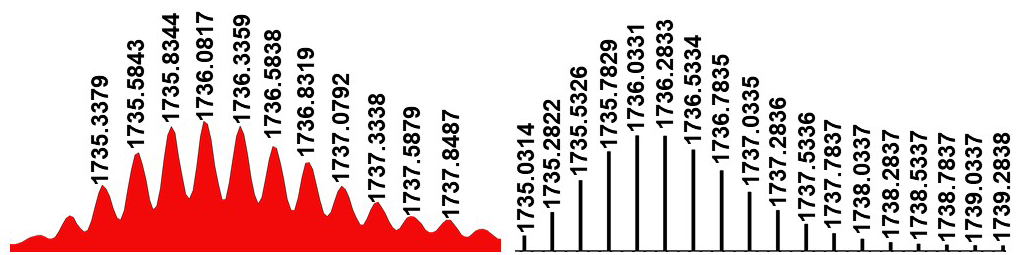
^

Figure S47. Experimental (right) and calculated (left) ESI-MS spectra of [5-BRs-4OTf]^4+^

^
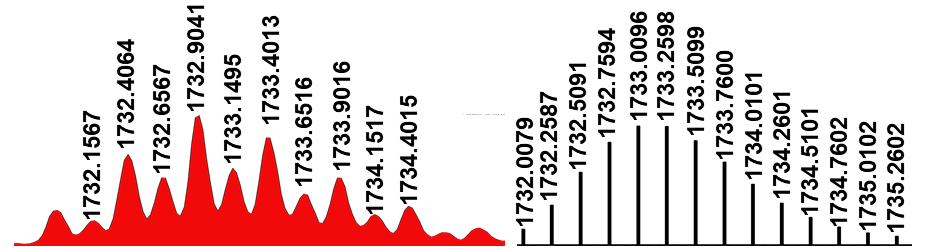
^

Figure S48. Experimental (right) and calculated (left) ESI-MS spectra of [6-IL-4OTf]^4+^


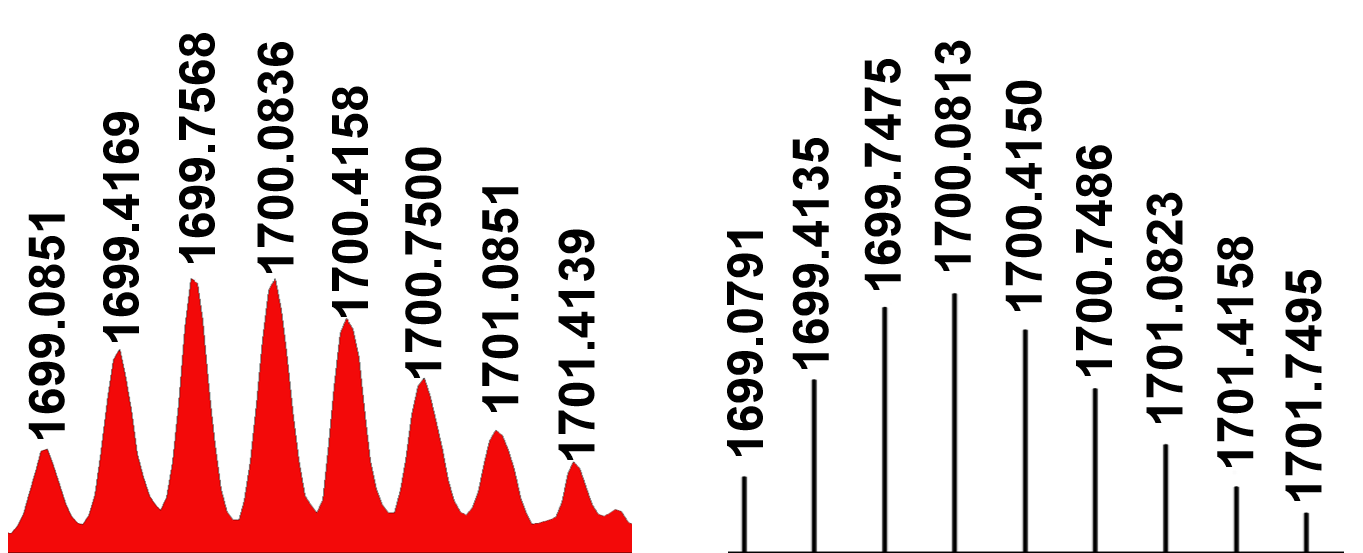


Figure S49. Experimental (right) and calculated (left) ESI-MS spectra of [10-IL-3OTf]^3+^


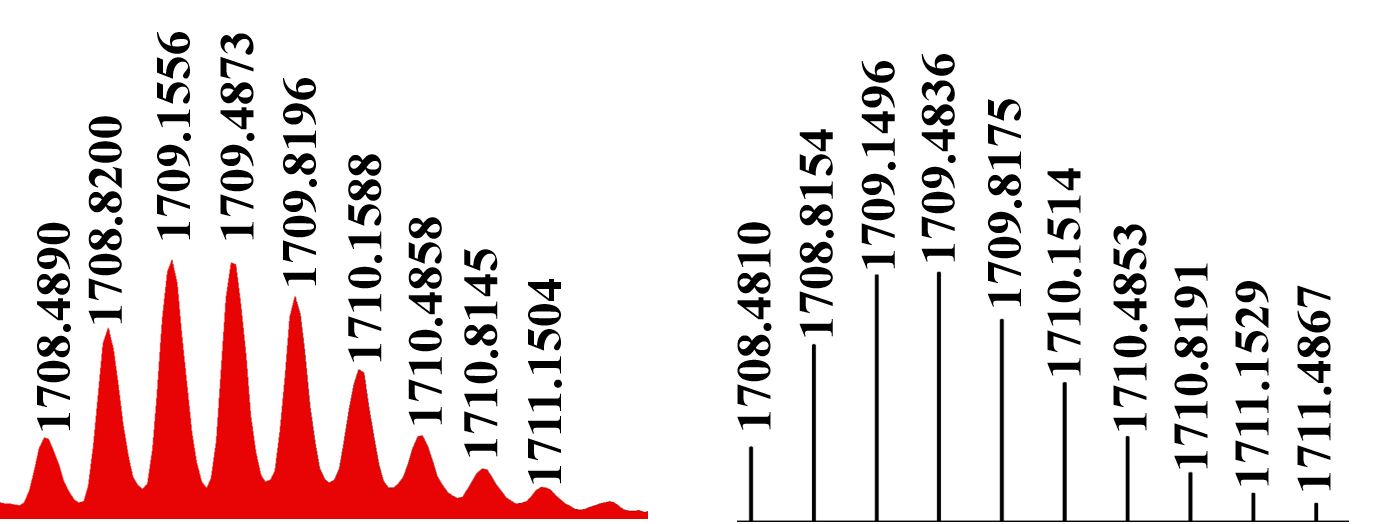


Figure S50. Experimental (right) and calculated (left) ESI-MS spectra of [11-IL-3OTf]^3+^

Single-crystal X-ray structures


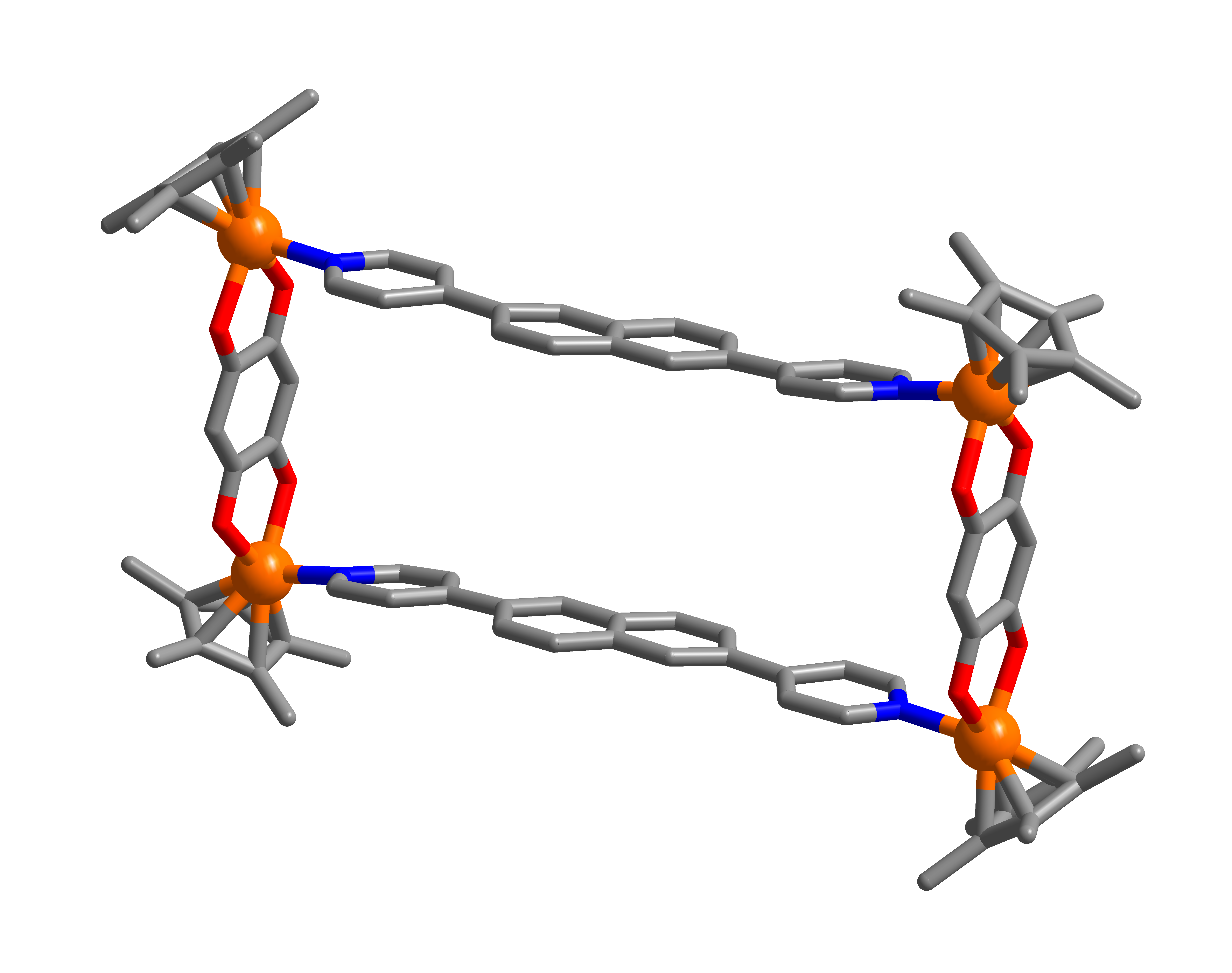


**Figure S51**. Single-crystal X-ray structures of cationic **2** (N, blue; O, red; C, black; Rh, orange), hydrogen atoms and counter anions are omitted.


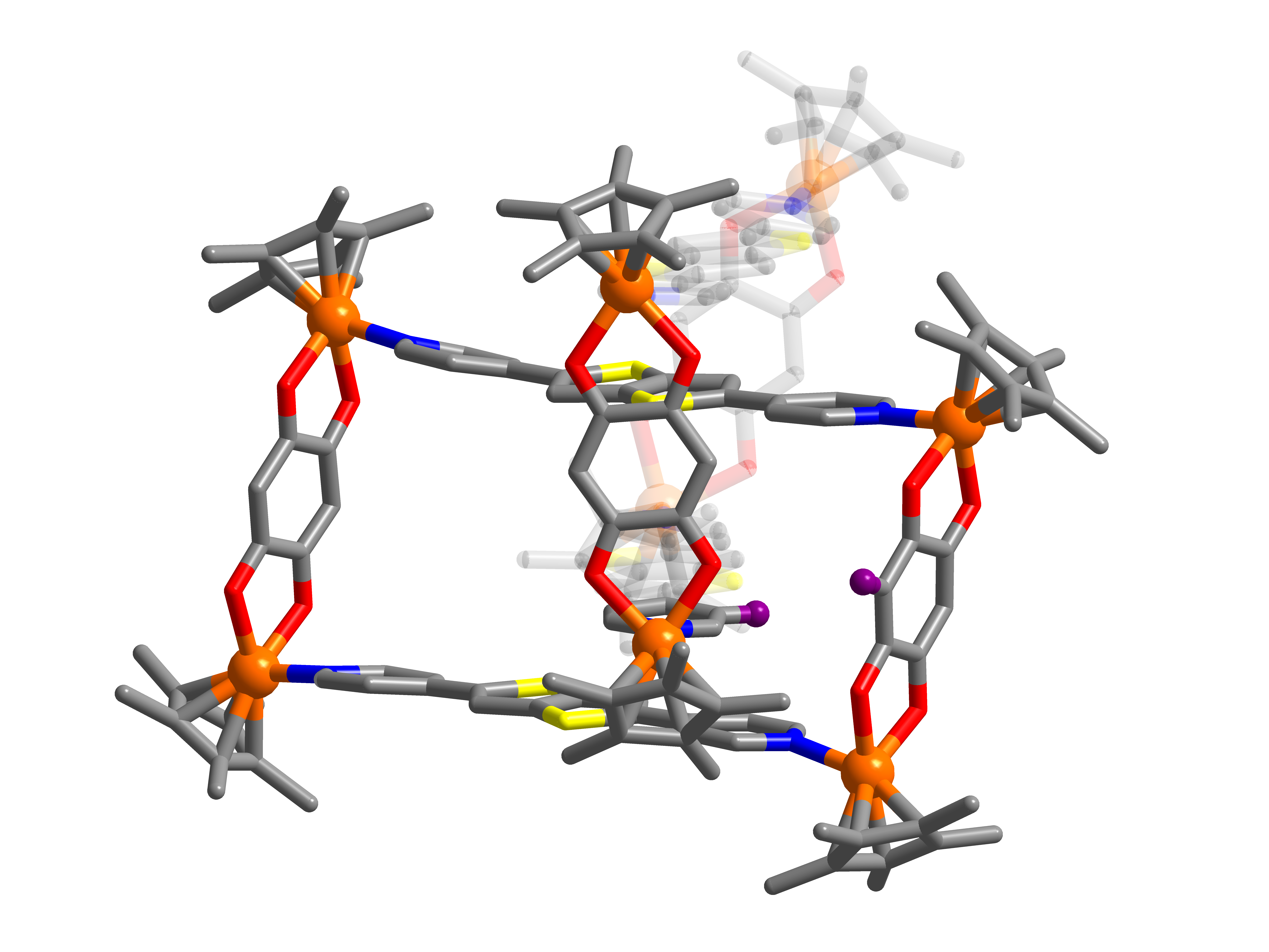


**Figure S52**. Single-crystal X-ray structures of cationic **3a-IL** (N, blue; O, red; S, yellow; C, black; Rh, orange), violet is two closely hydrogen atoms, other hydrogen atoms and counter anions are omitted.


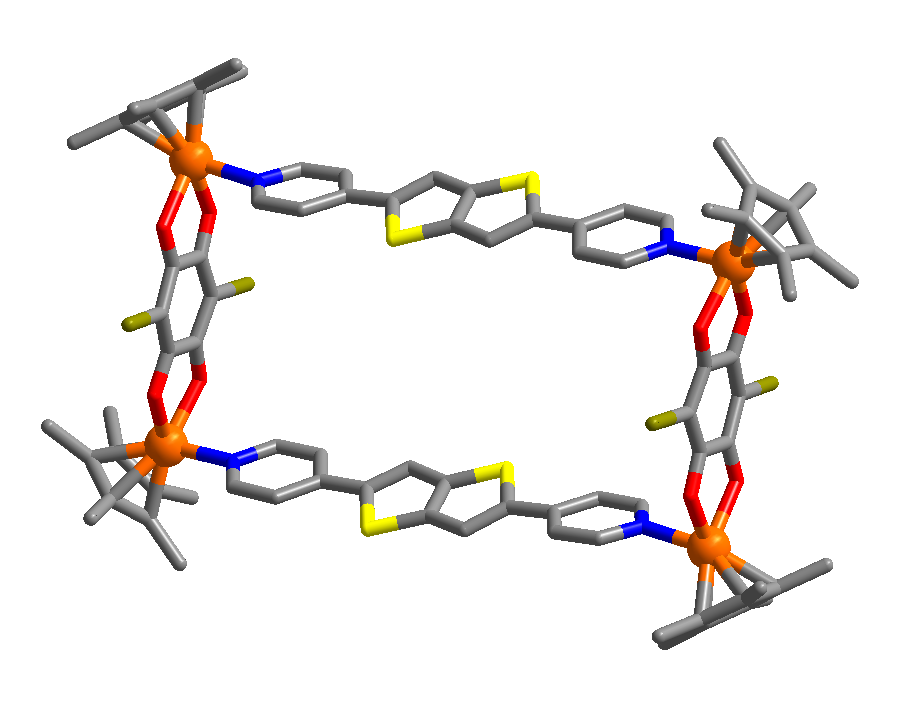


**Figure S53**. Single-crystal X-ray structures of cationic **3d** (N, blue; O, red; C, black; Rh, orange; S, yellow; Br, yellow-green), hydrogen atoms and counter anions are omitted.


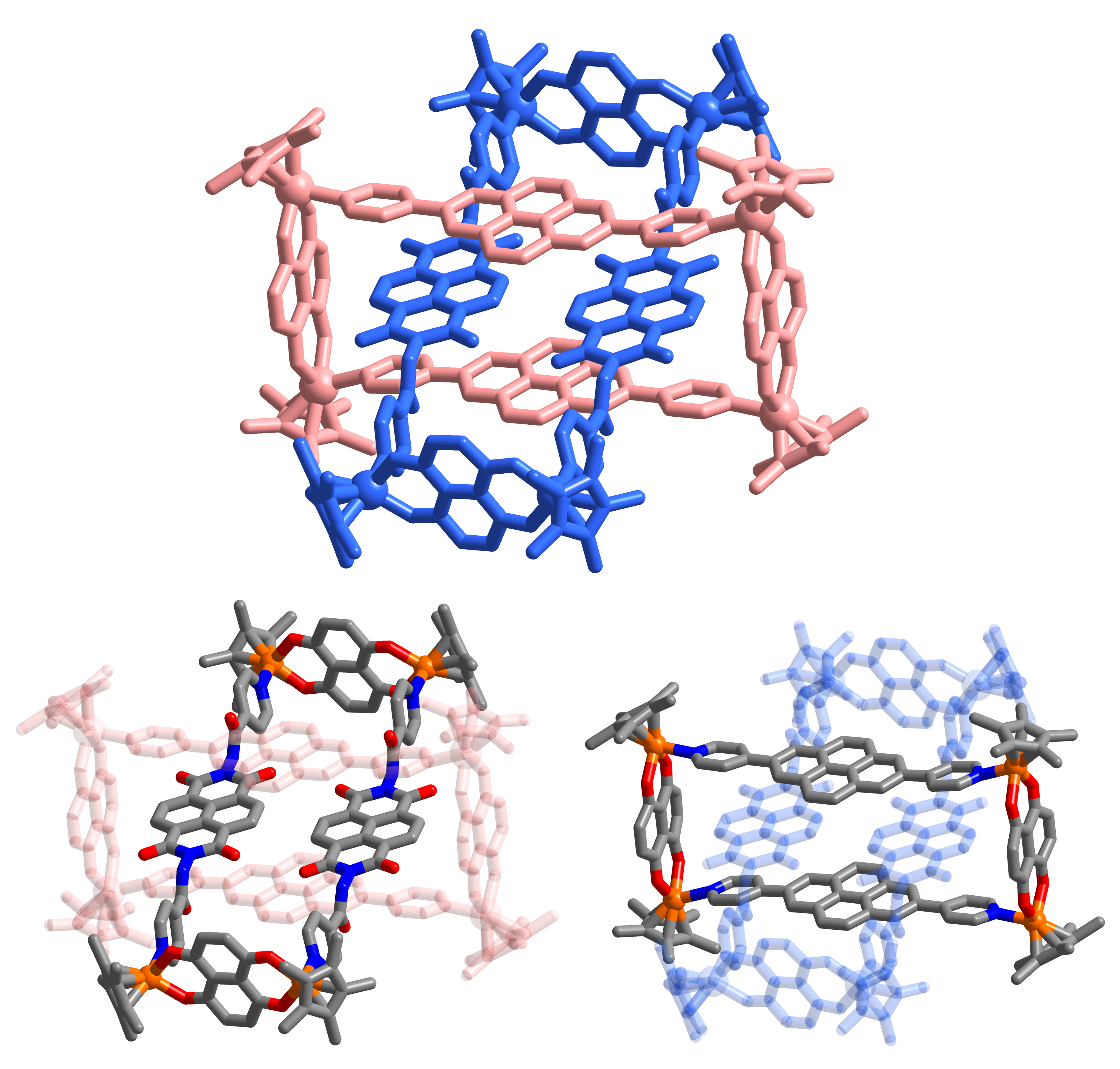


**Figure S54**. Single-crystal X-ray structures of cationic **11-IL** (N, blue; O, red; C, black; Rh, orange;), hydrogen atoms and counter anions are omitted.

# DFT Calculation

All density functional theory (DFT) calculations were carried out using the SIESTA package with numerical atomic orbital basis sets and Troullier-Martins norm-conserving pseudopotentials [S2, S3]. The DFT functional utilized is the PBE functional [S4], a generalized gradient approximation DFT method. To account for the long-range interaction, an empirical dispersion correction of Grimme was applied [S4]. A double-ζ plus polarization (DZP) basis set was employed. The orbital-confining cutoff radii were determined from an energy shift of 0.01 eV. The energy cutoff for the real space grid used to represent the density was set as 150 Ry. The molecule was placed in the center of a cubic supercell with a length of 50 Å for each edge. Only Γ-point was used to sample the Brillouin zone in our calculations due to the large lattice parameter of the supercell. All atoms were relaxed until all the Cartesian forces on the atoms were lower than 0.02 eV/Bohr.

According the results of DFT calculation, the binding energy of **10-IL** is obviously lower than heterogeneous [2]catenane.

**Table S1.** The binding energy (△H) and △G from monomers to dimers.

| Complex | Binding Energy (kcal/mol) |
| --- | --- |
| **3a-IL** | -59.5 |
| **10-IL** | -66.4 |
| Heterogeneous [2]catenane | -41.3 |

△G = △H - T·△S (T = 298 K, △S = –182.8 cal/mol/K)


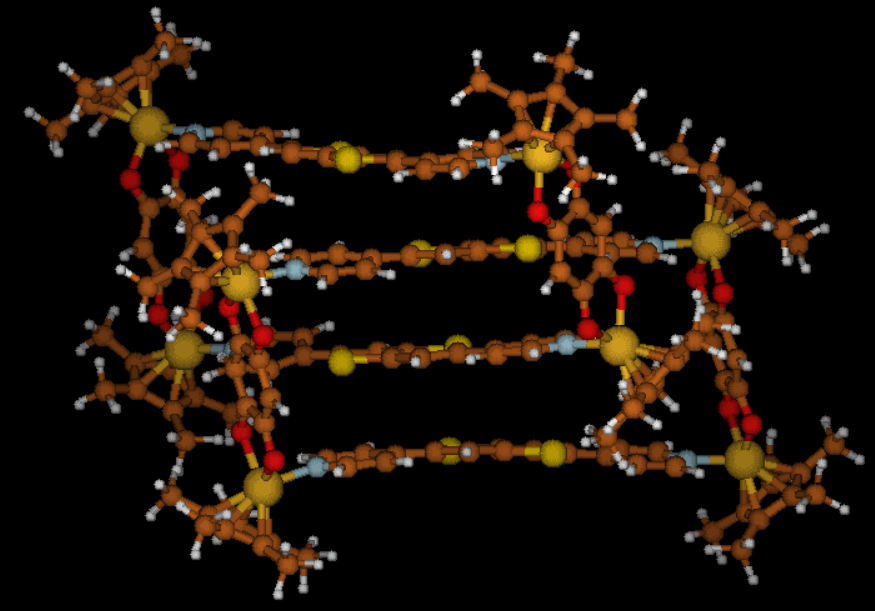

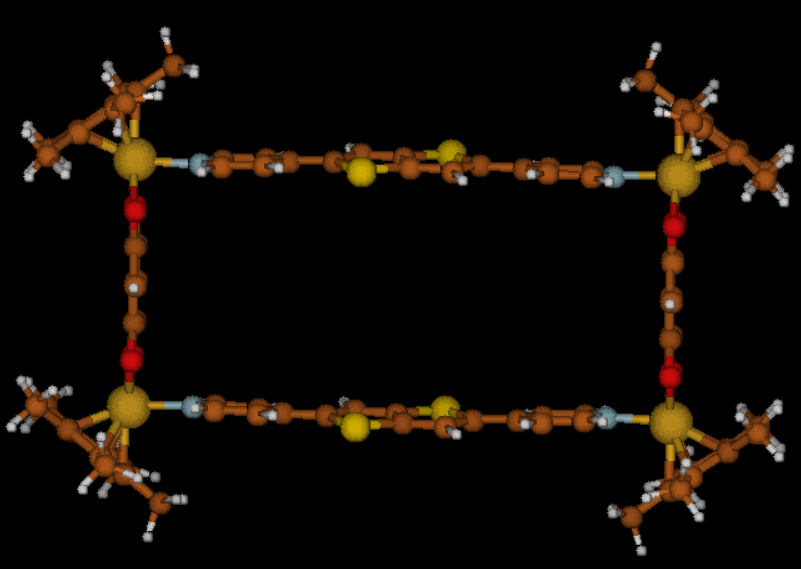


**Figure S55**.The total-energy density functional theory calculations of dimer (**3a-IL**) (left) and Monomer (**3a**) (right).


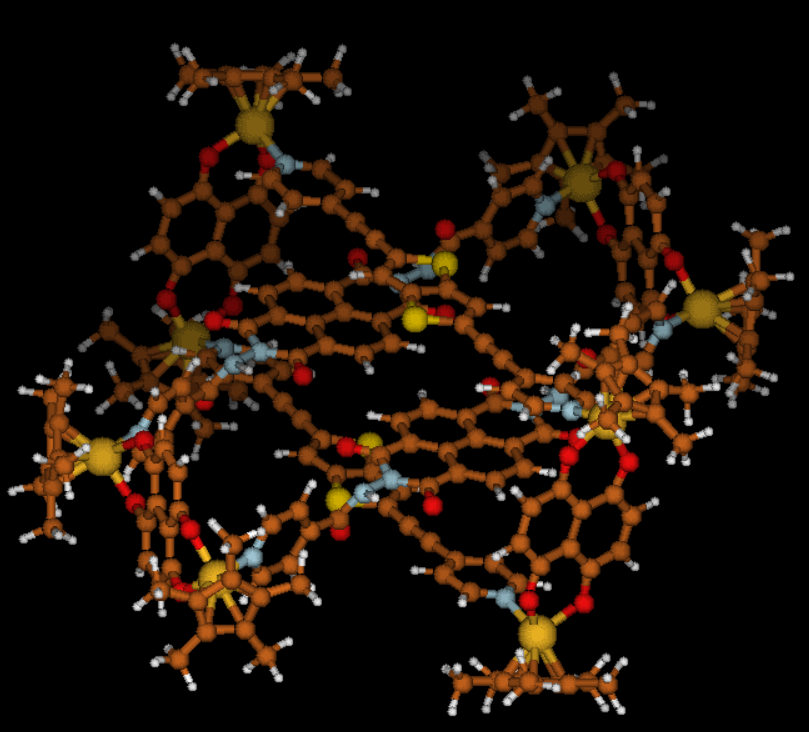


**Figure S56**.The total-energy density functional theory calculations of dimer (**10-IL**).


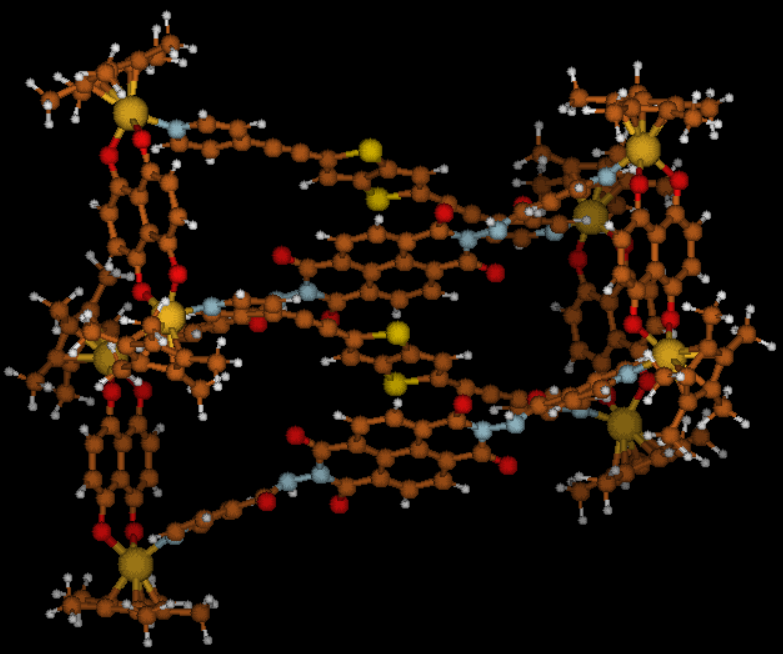


**Figure S57**.The total-energy density functional theory calculations of Heterogeneous [2]catenane.

# UV-VIS absorption spectra

**
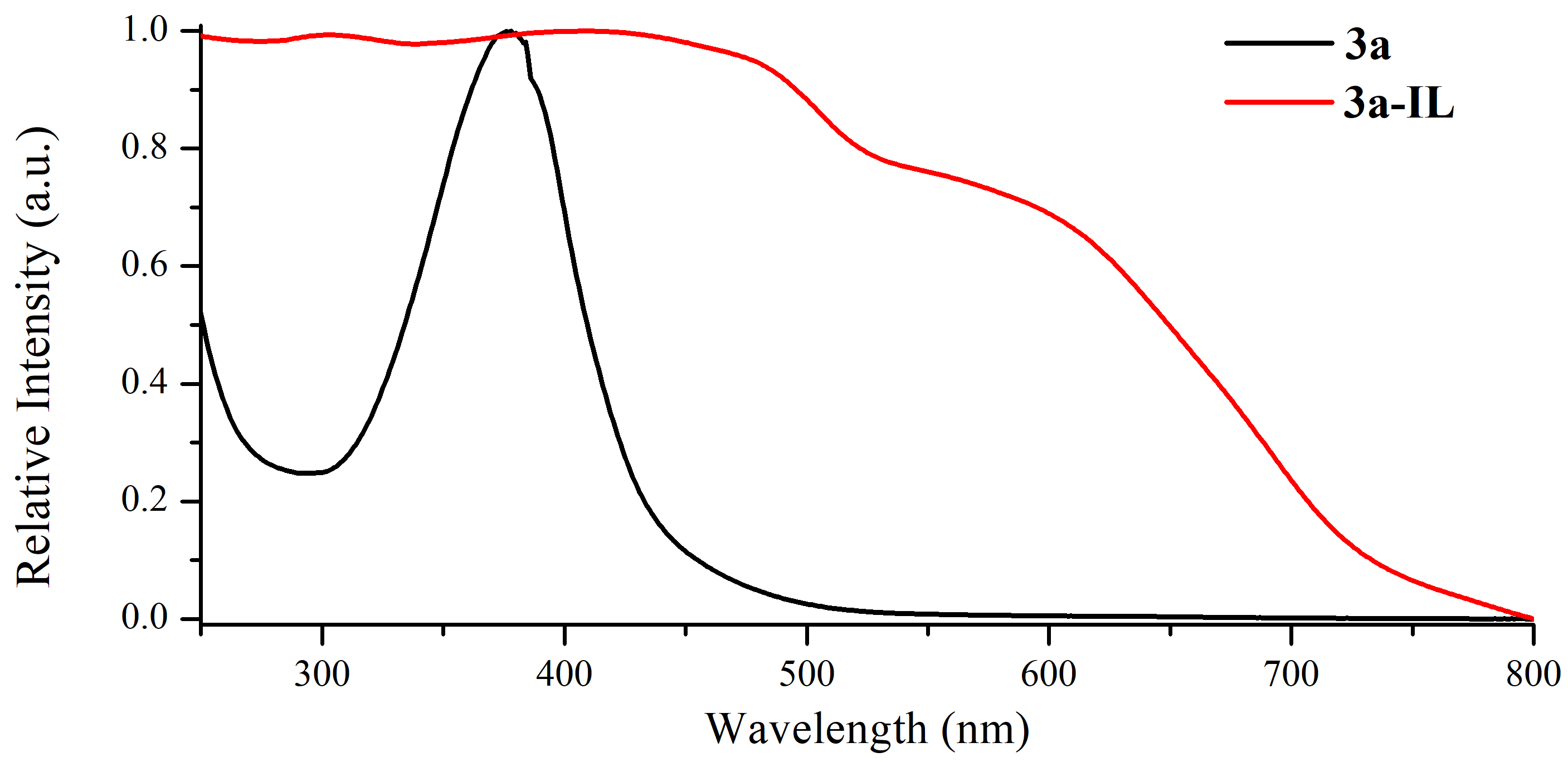
**

**Figure S58**. Normalized UV-Vis absorption spectra of **3a** (0.01 mM, methanol solution) (black) and **3a-IL** (Solid) (red). The absorption in visible area of **3a-IL** is obviously stronger than that of **3a**, which should be attributed to the π-π stacking among four bithiophenyl groups in the metalla[2]catenanes.


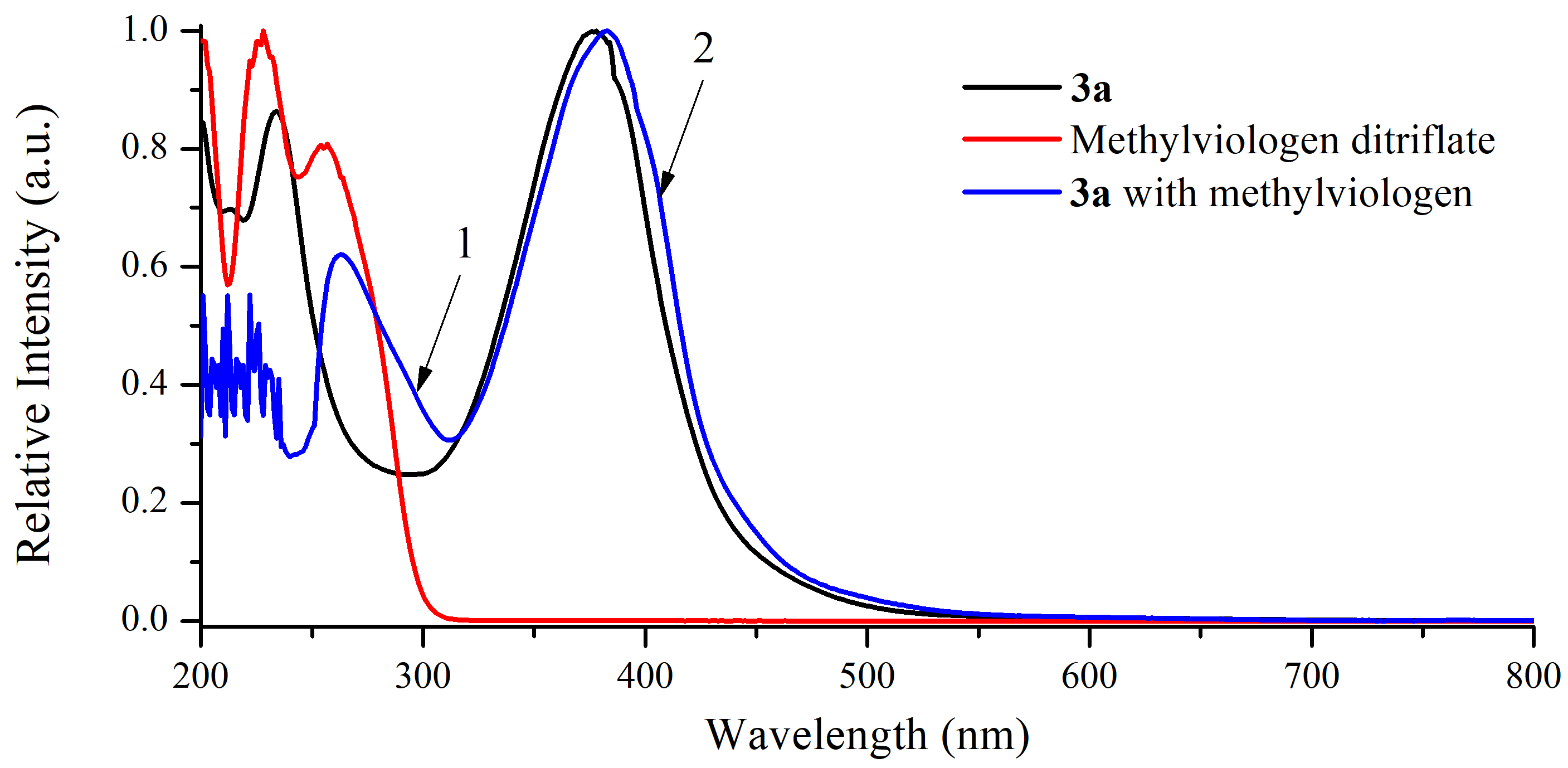


**Figure S59**. Normalized UV-Vis absorption spectra of **3a** (0.01 mM, methanol solution) (black), methylviologen ditriflate (0.01 mM, water solution) and **3a** with methylviologen ditriflate (0.01 mM, a mixed (3.0 mM) CHCl_3_ / CH_3_OH solution (1:1 v/v)). The absorption band of **3a** was red shifted after encapsulating methylviologen dication (1 and 2 area). This kind of red-shifted could be attributed to the charge transfer between metallarectangle **3a** and methylviologen guest.

**X-ray crystal data**

**Table S2.** Crystal data and structure refinement for **2**.

Empirical formula C112 H116 F12 N4 O22 Rh4 S4

Formula weight 2637.96

Temperature 173(2) K

Wavelength 0.71073 Å

Crystal system Monoclinic

Space group P2**_1_**/n

Unit cell dimensions a = 17.193(6) Å α = 90°.

b = 18.654(7) Å β = 106.808(5)°.

c = 19.175(7) Å γ = 90°.

Volume 5887(4) Å3

Z 2

Density (calculated) 1.488 Mg/m3

Absorption coefficient 0.709 mm-1

F(000) 2688

Crystal size 0.260 x 0.150 x 0.100 mm3

Theta range for data collection 1.403 to 25.250°.

Index ranges -20<=h<=20, -22<=k<=13, -23<=l<=23

Reflections collected 34996

Independent reflections 10602 [R(int) = 0.1209]

Completeness to theta = 25.242° 99.4 %

Absorption correction Semi-empirical from equivalents

Max. and min. transmission 0.647 and 0.374

Refinement method Full-matrix least-squares on F2

Data / restraints / parameters 10602 / 159 / 635

Goodness-of-fit on F2 1.071

Final R indices [I>2sigma(I)] R1 = 0.1057, wR2 = 0.2713

R indices (all data) R1 = 0.1877, wR2 = 0.3458

Extinction coefficient n/a

Largest diff. peak and hole 3.280 and -1.287 e.Å-3

[a] *R_1_* = Σ||*F0*|-|*Fc*|| (based on reflections with *F_0_^2^>2σF^2^*). *wR_2_* = [Σ[*w*(*F_0_*-*F_c_*^2^)^2^]/Σ[*w*(*F_0_*)^2^]]^1/2^; *w*=1/[*σ*^2^(*F_0_*^2^)+(0.095*P*)^2^]; *P* = [max(*F_0_*^2^,0)+2*F_c_*^2^ ]/3(also with *F_0_*^2^>2*σF*^2^)

**Table S3.** Crystal data and structure refinement for **3a-IL**.

Empirical formula C90 H111 F12 N4 O31.50 Rh4 S8

Formula weight 2648.94

Temperature 203(2) K

Wavelength 1.34139 Å

Crystal system Monoclinic

Space group P2**_1_**

Unit cell dimensions a = 23.7730(6) Å α = 90°.

b = 17.4234(4) Å β = 99.9480(10)°.

c = 28.7170(8) Å γ = 90°.

Volume 11715.9(5) Å3

Z 4

Density (calculated) 1.502 Mg/m3

Absorption coefficient 4.428 mm-1

F(000) 5388

Crystal size 0.250 x 0.220 x 0.180 mm3

Theta range for data collection 3.191 to 57.105°.

Index ranges -29<=h<=29, -21<=k<=21, -34<=l<=35

Reflections collected 123167

Independent reflections 47339 [R(int) = 0.0594]

Completeness to theta = 53.594° 99.6 %

Absorption correction Semi-empirical from equivalents

Refinement method Full-matrix least-squares on F2

Data / restraints / parameters 47339 / 177 / 2049

Goodness-of-fit on F2 0.947

Final R indices [I>2sigma(I)] R1 = 0.0542, wR2 = 0.1511

R indices (all data) R1 = 0.0679, wR2 = 0.1605

Absolute structure parameter 0.168(8)

Extinction coefficient 0.00029(6)

Largest diff. peak and hole 0.838 and -0.607 e.Å-3

[a] *R_1_* = Σ||*F0*|-|*Fc*|| (based on reflections with *F_0_^2^>2σF^2^*). *wR_2_* = [Σ[*w*(*F_0_*-*F_c_*^2^)^2^]/Σ[*w*(*F_0_*)^2^]]^1/2^; *w*=1/[*σ*^2^(*F_0_*^2^)+(0.095*P*)^2^]; *P* = [max(*F_0_*^2^,0)+2*F_c_*^2^ ]/3(also with *F_0_*^2^>2*σF*^2^)

**Table S4.** Crystal data and structure refinement for **5-BRs**.

Empirical formula C106 H126.67 F12 N4 O29.33 Rh4 S8.67

Formula weight 2843.60

Temperature 173.0 K

Wavelength 1.34138 Å

Crystal system Triclinic

Space group P-1

Unit cell dimensions a = 23.504(5) Å α = 88.722(8)°.

b = 23.695(6) Å β = 89.147(8)°.

c = 36.877(9) Å γ = 70.040(7)°.

Volume 19300(8) Å3

Z 6

Density (calculated) 1.468 Mg/m3

Absorption coefficient 4.060 mm-1

F(000) 8712

Crystal size 0.64 x 0.62 x 0.55 mm3

Theta range for data collection 2.915 to 54.085°.

Index ranges -28<=h<=28, -28<=k<=28, -44<=l<=44

Reflections collected 230412

Independent reflections 70210 [R(int) = 0.0519]

Completeness to theta = 53.594° 99.5 %

Absorption correction Semi-empirical from equivalents

Max. and min. transmission 0.3484 and 0.1702

Refinement method Full-matrix least-squares on F2

Data / restraints / parameters 70210 / 1027 / 3521

Goodness-of-fit on F2 1.029

Final R indices [I>2sigma(I)] R1 = 0.0597, wR2 = 0.1647

R indices (all data) R1 = 0.0705, wR2 = 0.1730

Extinction coefficient n/a

Largest diff. peak and hole 1.854 and -1.143 e.Å-3

[a] *R_1_* = Σ||*F0*|-|*Fc*|| (based on reflections with *F_0_^2^>2σF^2^*). *wR_2_* = [Σ[*w*(*F_0_*-*F_c_*^2^)^2^]/Σ[*w*(*F_0_*)^2^]]^1/2^; *w*=1/[*σ*^2^(*F_0_*^2^)+(0.095*P*)^2^]; *P* = [max(*F_0_*^2^,0)+2*F_c_*^2^ ]/3(also with *F_0_*^2^>2*σF*^2^)

**Table S5.** Crystal data and structure refinement for **6-IL**.

Empirical formula C106 H124 F12 N4 O30 Rh4 S10

Formula weight 2894.32

Temperature 173(2) K

Wavelength 1.3418 Å

Crystal system Triclinic

Space group P-1

Unit cell dimensions a = 17.9796(4) Å α = 76.7170(10)°.

b = 19.6889(4) Å β = 75.8650(10)°.

c = 31.2999(6) Å γ = 72.1960(10)°.

Volume 10084.7(4) Å3

Z 3

Density (calculated) 1.430 Mg/m3

Absorption coefficient 4.073 mm-1

F(000) 4428

Crystal size 0.150 x 0.120 x 0.060 mm3

Theta range for data collection 2.826 to 55.047°.

Index ranges -21<=h<=21, -22<=k<=23, -38<=l<=38

Reflections collected 105034

Independent reflections 37968 [R(int) = 0.1027]

Completeness to theta = 53.618° 99.3 %

Absorption correction Semi-empirical from equivalents

Max. and min. transmission 0.578 and 0.428

Refinement method Full-matrix least-squares on F2

Data / restraints / parameters 37968 / 573 / 1772

Goodness-of-fit on F2 0.981

Final R indices [I>2sigma(I)] R1 = 0.0897, wR2 = 0.2509

R indices (all data) R1 = 0.1905, wR2 = 0.3210

Extinction coefficient n/a

Largest diff. peak and hole 1.711 and -0.834 e.Å-3

[a] *R_1_* = Σ||*F0*|-|*Fc*|| (based on reflections with *F_0_^2^>2σF^2^*). *wR_2_* = [Σ[*w*(*F_0_*-*F_c_*^2^)^2^]/Σ[*w*(*F_0_*)^2^]]^1/2^; *w*=1/[*σ*^2^(*F_0_*^2^)+(0.095*P*)^2^]; *P* = [max(*F_0_*^2^,0)+2*F_c_*^2^ ]/3(also with *F_0_*^2^>2*σF*^2^)

**Table S6.** Crystal data and structure refinement for **3d**.

Empirical formula C106 H138 Br4 F12 N4 O31 Rh4 S17

Formula weight 3468.50

Temperature 173(2) K

Wavelength 1.34138 Å

Crystal system Monoclinic

Space group C2/c

Unit cell dimensions a = 44.770(4) Å α = 90°.

b = 15.0395(7) Å β = 119.444(6)°.

c = 22.3166(16) Å γ = 90°.

Volume 13085.2(17) Å3

Z 4

Density (calculated) 1.761 Mg/m3

Absorption coefficient 5.855 mm-1

F(000) 7000

Crystal size 0.150 x 0.130 x 0.030 mm3

Theta range for data collection 2.740 to 54.178°.

Index ranges -40<=h<=54, -17<=k<=18, -26<=l<=26

Reflections collected 44760

Independent reflections 11979 [R(int) = 0.0578]

Completeness to theta = 53.594° 99.4 %

Absorption correction Semi-empirical from equivalents

Max. and min. transmission 0.702 and 0.420

Refinement method Full-matrix least-squares on F2

Data / restraints / parameters 11979 / 55 / 644

Goodness-of-fit on F2 0.918

Final R indices [I>2sigma(I)] R1 = 0.0526, wR2 = 0.1537

R indices (all data) R1 = 0.0641, wR2 = 0.1678

Extinction coefficient n/a

Largest diff. peak and hole 1.563 and -0.783 e.Å-3

[a] *R_1_* = Σ||*F0*|-|*Fc*|| (based on reflections with *F_0_^2^>2σF^2^*). *wR_2_* = [Σ[*w*(*F_0_*-*F_c_*^2^)^2^]/Σ[*w*(*F_0_*)^2^]]^1/2^; *w*=1/[*σ*^2^(*F_0_*^2^)+(0.095*P*)^2^]; *P* = [max(*F_0_*^2^,0)+2*F_c_*^2^ ]/3(also with *F_0_*^2^>2*σF*^2^)

**Table S7.** Crystal data and structure refinement for **3b** with encapsulated methylviologen.

Empirical formula C110 H122 F22 N6 O32 Rh4 S12

Formula weight 3254.49

Temperature 173(2) K

Wavelength 1.34138 Å

Crystal system Monoclinic

Space group P2**_1_**/c

Unit cell dimensions a = 14.4765(11) Å α = 90°.

b = 34.431(2) Å β = 115.559(2)°.

c = 15.5889(10) Å γ = 90°.

Volume 7009.9(9) Å3

Z 2

Density (calculated) 1.542 Mg/m3

Absorption coefficient 4.209 mm-1

F(000) 3300

Crystal size 0.300 x 0.120 x 0.030 mm3

Theta range for data collection 2.944 to 53.500°.

Index ranges -17<=h<=17, -31<=k<=41, -16<=l<=18

Reflections collected 56139

Independent reflections 12634 [R(int) = 0.0459]

Completeness to theta = 53.500° 99.8 %

Absorption correction Semi-empirical from equivalents

Max. and min. transmission 0.751 and 0.368

Refinement method Full-matrix least-squares on F2

Data / restraints / parameters 12634 / 346 / 647

Goodness-of-fit on F2 1.043

Final R indices [I>2sigma(I)] R1 = 0.0745, wR2 = 0.2461

R indices (all data) R1 = 0.0901, wR2 = 0.2627

Extinction coefficient n/a

Largest diff. peak and hole 1.515 and -0.767 e.Å-3

[a] *R_1_* = Σ||*F0*|-|*Fc*|| (based on reflections with *F_0_^2^>2σF^2^*). *wR_2_* = [Σ[*w*(*F_0_*-*F_c_*^2^)^2^]/Σ[*w*(*F_0_*)^2^]]^1/2^; *w*=1/[*σ*^2^(*F_0_*^2^)+(0.095*P*)^2^]; *P* = [max(*F_0_*^2^,0)+2*F_c_*^2^ ]/3(also with *F_0_*^2^>2*σF*^2^)

**Table S8.** Crystal data and structure refinement for **10-IL**.

Empirical formula C228 H228 F24 N16 O66 Rh8 S12

Formula weight 5912.25

Temperature 173(2) K

Wavelength 1.3418 Å

Crystal system Monoclinic

Space group P2**_1_**/n

Unit cell dimensions a = 10.2759(4) Å α = 90°.

b = 45.0309(14) Å β = 93.240(2)°.

c = 28.7205(9) Å γ = 90°.

Volume 13268.7(8) Å3

Z 2

Density (calculated) 1.480 Mg/m3

Absorption coefficient 3.775 mm-1

F(000) 6008

Crystal size 0.550 x 0.080 x 0.080 mm3

Theta range for data collection 2.892 to 56.124°.

Index ranges -12<=h<=12, -55<=k<=55, -27<=l<=35

Reflections collected 99832

Independent reflections 26055 [R(int) = 0.1153]

Completeness to theta = 53.618° 99.8 %

Absorption correction Semi-empirical from equivalents

Max. and min. transmission 0.657 and 0.342

Refinement method Full-matrix least-squares on F2

Data / restraints / parameters 26055 / 663 / 1574

Goodness-of-fit on F2 0.984

Final R indices [I>2sigma(I)] R1 = 0.0880, wR2 = 0.2527

R indices (all data) R1 = 0.1826, wR2 = 0.3244

Extinction coefficient 0.00013(3)

Largest diff. peak and hole 0.681 and -0.832 e.Å-3

[a] *R_1_* = Σ||*F0*|-|*Fc*|| (based on reflections with *F_0_^2^>2σF^2^*). *wR_2_* = [Σ[*w*(*F_0_*-*F_c_*^2^)^2^]/Σ[*w*(*F_0_*)^2^]]^1/2^; *w*=1/[*σ*^2^(*F_0_*^2^)+(0.095*P*)^2^]; *P* = [max(*F_0_*^2^,0)+2*F_c_*^2^ ]/3(also with *F_0_*^2^>2*σF*^2^)

**Table S9.** Crystal data and structure refinement for **11-IL**.

Empirical formula C254 H346 F24 N18 O106 Rh8 S8

Formula weight 6883.23

Temperature 173(2) K

Wavelength 1.34138 Å

Crystal system Monoclinic

Space group C2/c

Unit cell dimensions a = 48.422(3) Å α = 90°.

b = 21.5406(13) Å β = 111.406(2)°.

c = 33.652(2) Å γ = 90°.

Volume 32679(3) Å3

Z 4

Density (calculated) 1.399 Mg/m3

Absorption coefficient 2.983 mm-1

F(000) 14192

Crystal size 0.250 x 0.220 x 0.180 mm3

Theta range for data collection 1.705 to 56.151°.

Index ranges -59<=h<=59, -26<=k<=26, -40<=l<=41

Reflections collected 239559

Independent reflections 32243 [R(int) = 0.0737]

Completeness to theta = 53.594° 99.9 %

Absorption correction Semi-empirical from equivalents

Max. and min. transmission 0.513 and 0.179

Refinement method Full-matrix least-squares on F2

Data / restraints / parameters 32243 / 481 / 1479

Goodness-of-fit on F2 1.020

Final R indices [I>2sigma(I)] R1 = 0.0853, wR2 = 0.2608

R indices (all data) R1 = 0.1066, wR2 = 0.2873

Extinction coefficient n/a

Largest diff. peak and hole 1.153 and -1.443 e.Å-3

[a] *R_1_* = Σ||*F0*|-|*Fc*|| (based on reflections with *F_0_^2^>2σF^2^*). *wR_2_* = [Σ[*w*(*F_0_*-*F_c_*^2^)^2^]/Σ[*w*(*F_0_*)^2^]]^1/2^; *w*=1/[*σ*^2^(*F_0_*^2^)+(0.095*P*)^2^]; *P* = [max(*F_0_*^2^,0)+2*F_c_*^2^ ]/3(also with *F_0_*^2^>2*σF*^2^)

**Reference**

S1. Lu Y, Lin YJ and Li ZH *et al.* Highly stable molecular Borromean rings. *Chin J Chem* 2018; **36**: 106-11.

S2. Junquera J, Paz O and Sanchez-Portal D. *et al.* Numerical atomic orbitals for linear-scaling calculations. *Phys Rev B* 2001; **23**: *64*.

S3. Troullier N and Martins, JL Efficient pseudopotentials for plane-wave calculations. *Phys Rev B* 1991; **43**: 1993-2006.

S4. Perdew JP, Burke K. and Ernzerhof M Generalized gradient approximation made simple. *Phys Rev Lett* 1996; **77**: 3865-8.

S5. Grimme S Semiempirical GGA-type density functional constructed with a long-range dispersion correction. *J Comput Chem* 2006; **27**: 1787-99.
